# SenescenceBioKG reveals full-text-supported biomaterial design, mechanisms, and regeneration patterns in senescence-oriented tissue engineering

**DOI:** 10.3389/fbioe.2026.1966523

**Published:** 2026-10-29

**Authors:** Shengyue Li, Xinxin Jing, Jin Tan, Qiang Zhang

**Affiliations:** 1 Department of Geriatrics, Tianjin Medical University General Hospital, Tianjin, China; 2 Ministry of Education Key Laboratory of Post-Neuroinjury Neuro-Repair and Regeneration in the Central Nervous System, Tianjin, China; 3 State Key Laboratory of Experimental Hematology, Tianjin, China; 4 Tianjin Key Laboratory of Elderly Health, Tianjin, China; 5 Tianjin Geriatrics Institute, Tianjin, China

**Keywords:** biomaterials, cellular senescence, full-text evidence, knowledge graph, regenerative medicine, smart materials, stimulus-responsive materials, tissue engineering

## Abstract

**Background:**

Senescence-oriented biomaterial studies provide evidence regarding material design, responsive behavior, cellular targets, mechanisms, regeneration, and validation across different full-text sections. However, publication-level labels cannot reliably answer questions that require several of these relations to occur in the same study.

**Methods:**

We constructed SenescenceBioKG based on 283 original experimental or translational full-text studies. Candidate relations were generated under a typed ontology, adjudicated against same-record evidence, linked to unique evidence identifiers, and canonicalized into a queryable graph. Fifteen competency questions were evaluated using graph traversal and an independently implemented dataframe method. A local read-only R Shiny Explorer was developed for evidence inspection.

**Results:**

The frozen graph contained 654 canonical nodes, 1,868 supported edges, and 1,868 linked evidence records. Within the curated corpus, strict material responsiveness comprised 64 RESPONDS TO edges from 55 studies and 55 material platforms, led by acidic pH and reactive oxygen species. Same-study analyses recovered responsive-design modules and 35 complete target-cell–mechanism–regeneration modules. Mesenchymal stem/stromal cells, senescent cells, and macrophage or innate immune populations were recurrent targets, whereas oxidative-stress control, mitochondrial homeostasis, and NF-κB/Senescence-associated secretory phenotype (SASP) signaling frequently accompanied bone, wound/skin, cartilage, and disc endpoints. Validation was concentrated in Cell and Small-Animal studies; Organoid/chip and Large-Animal evidence was sparse, and no Clinical edge was supported. All 15 queries achieved exact-set agreement between the two implementations, with macro precision, recall, and F1 values of 1.00.

**Conclusion:**

SenescenceBioKG provides a full-text-supported structure for examining smart and responsive biomaterial design, fabrication, cellular evidence, regenerative outcomes, and validation gaps. Its provenance model separates co-supported evidence from causal inference and supports reproducible, material-centered retrieval.

## 1 Introduction

Cellular senescence combines durable cell-cycle arrest with stress signaling and a modified secretory state (Rhinn et al., 2019; Di Micco et al., 2021). Its impact on repair depend on timing and context: transient senescence can support wound resolution, whereas persistent senescent cells can disturb progenitor, stromal, immune, vascular, and parenchymal compartments (Demaria et al., 2014; Shvedova et al., 2022; Wilkinson and Hardman, 2022). Biomaterials offer a means to localize interventions, control exposure, shape cell–material interactions, and support tissue reconstruction. Therefore, they offer engineering routes to act on senescent cells, protect repair-competent cells, or modify a senescence-associated microenvironment while retaining a regenerative function (Li et al., 2021; Zhang et al., 2025).

Senescence-oriented platforms include drug carriers, hydrogels, nanoparticles, scaffolds, extracellular-matrix constructs, surface-modified implants, and systems containing cells or extracellular vesicles (Zhao et al., 2025). They can provide local retention, controlled delivery, immune regulation, and redox or mitochondrial control (Dai et al., 2022; Zhang et al., 2023; Han et al., 2024). In explicitly responsive systems, a defined cue, such as acidic pH, reactive oxygen species, mechanical input, light, or an enzyme, changes material behavior (Badeau and DeForest, 2019; Wei et al., 2022; Liao et al., 2025). Surface coating, self-assembly, electrospinning, photocrosslinking, microfluidics, and 3D printing or bioprinting provide distinct fabrication routes (Gao et al., 2023; Chen et al., 2024; Rahimnejad et al., 2024). Neither an engineering label nor additive manufacturing alone establishes responsiveness, mechanism, or regenerative benefit.

The evidence needed to evaluate these systems is distributed within each article. Composition and fabrication are usually described in the Methods, whereas release profiles, target cells, molecular responses, and tissue endpoints appear in the Results, Figures, or Tables. Similar wording may conceal different claims. A material can lower cellular reactive oxygen species without responding to that stimulus, carry a therapeutic cargo without showing release, or improve tissue morphology without a direct functional measurement. Patient-derived cells or tissues provide human-derived evidence, not evidence of a clinical intervention.

Therefore, reliable synthesis requires more than publication-level labels. Mentions of a stimulus cannot identify materials that undergo a stimulus-dependent change, and pathway terms cannot separate measured mechanisms from cited explanations. Animal intervention, human-derived material, and treatment of human participants also represent different validation settings. The material platform, asserted relation, experimental setting, and supporting passage must remain linked. Plausible but unsupported relations should be retained for audit while remaining outside the analytical graph. These errors also have different analytical consequences. A false response edge inflates the apparent prevalence of material intelligence, an incorrect cargo edge changes the inferred delivery design, and an unsupported mechanism edge can create a biological path that the study never tested. Therefore, treating each relation separately is necessary for interpretable counts and multi-relation queries.

A full-text relation graph can address this problem through explicit entity and relation definitions, domain and range constraints, canonical identifiers, and same-record provenance (Humayun et al., 2020; Chandak et al., 2023). It differs from keyword co-occurrence, publication-trend analysis, and citation networks as each accepted edge requires direct support from the source study. Other biomedical knowledge-graph frameworks have used semantic retrieval and automated literature-derived relation extraction for structured biomedical information access (Ebeid, 2022; Li et al., 2022). Such a graph can distinguish RESPONDS TO from antioxidant activity, DELIVERS from RELEASES, and Human-derived from Clinical evidence. Furthermore, it requires stimulus response, target cells, mechanisms, endpoints, and validation to coexist within one Record ID and MaterialPlatform without treating their co-occurrence as a causal chain.

Materials studies are well suited to this relation-level representation as one platform can support several experimentally distinct claims. Its constituents and fabrication process may be documented separately from stimulus-dependent behavior, cellular testing, molecular responses, or regenerative outcomes. Separate evidence links retain that modular structure and render complete and interrupted evidence chains reproducible. This organization also allows a query to return the study and material that support a path, rather than only a global connection between frequently shared entities.

We developed SenescenceBioKG as a full-text-supported knowledge graph of senescence-oriented biomaterials in tissue repair and regeneration. The study reconstructed and validated material-centered relations from a fixed corpus; quantified responsive design, fabrication, and cell–mechanism–regeneration patterns; characterized validation coverage and defined gaps in the supported graph; and tested whether the frozen graph enabled reproducible competency queries and evidence retrieval using an interactive Explorer. Graph connectivity was interpreted as corpus representation, not as biological importance or therapeutic superiority.

## 2 Materials and methods

### 2.1 Study design and full-text corpus

This evidence-based computational biomaterials study used a literature-derived full-text corpus. Searches of Web of Science Core Collection, Scopus, and PubMed/MEDLINE until 21 July 2026 retrieved 2,256 records: 685, 1,023, and 548 from the respective databases (Supplementary Table S1). Identifier and title normalization removed 1,195 duplicates, leaving 1,061 records for title and abstract screening. Initial screening excluded 731 records and retained 330. Enhanced abstract adjudication excluded another 19 records, generating a 311-record contextual corpus comprising 283 original experimental or translational studies, 16 reviews, and 12 boundary records. Only the 283 original studies entered the core graph.

Eligibility required an engineered material, formulation, carrier, surface, scaffold, construct, or related platform; direct assessment or intervention involving cellular senescence or a senescence-associated state; and a tissue-repair, regenerative, or function-oriented endpoint. Original studies were eligible across cell, *ex vivo*, organoid/chip, animal, and human-derived settings. Reviews provided context but were not encoded as direct evidence. Full-text PDFs were available for all included studies and matched to fixed Record ID values. The complete bibliographic roster of the 283 core studies, including their fixed Record IDs, is provided in Supplementary Table S2. Background statements, reference lists, and descriptions of other studies in the Introduction or Discussion were not accepted as core relation evidence.

### 2.2 Ontology construction and candidate relation generation

The ontology defined 14 entity types: Study, MaterialPlatform, MaterialComposition, FabricationStrategy, DesignMode, Stimulus, ResponsiveBehavior, TherapeuticCargo, TargetCell, SenescenceEvidence, Mechanism, DiseaseModel, RegenerativeEndpoint, and ValidationStage. Ten types were instantiated in the frozen graph (Supplementary Table S3). Study was retained as an ontology-defined entity type but was not instantiated as a canonical graph node; publication-level provenance was represented by the fixed Record ID. MaterialPlatform was the principal source entity. Permitted relations were USES, COMPOSED OF, FABRICATED BY, RESPONDS TO, RELEASES, DELIVERS, TARGETS, MODULATES, ACTIVATES, SUPPRESSES, PROMOTES, and VALIDATED AT. Domain and range constraints prevented methods text, assay reagents, downstream proteins, or result sentences from being represented as material constituents or cargo.

Structured full-text fields generated candidate edges under these constraints. Entity names were normalized for Unicode, Greek letters, capitalization, spacing, hyphenation, singular and plural forms, and confirmed synonyms. Canonical Node ID values were assigned without changing Record ID. Merges were allowed only for the same scientific entity; uncertain equivalence was flagged. Automated scripts supported parsing, identifier checks, aggregation, and duplicate detection, whereas scientific classification was determined through manual review at the edge level. Ambiguous entity assignments, cargo classifications, mechanism annotations, validation-stage assignments, and suspected semantic contamination were rechecked against the corresponding full text. Relations that could not be resolved using direct same-record evidence were retained as UNCLEAR rather than forced into a binary decision. Formal interrater agreement was not assessed; instead, reliability was supported by discrepancy review, semantic audits, evidence-linkage checks, and deterministic quality-control procedures.

Study and material identities were kept separate. Record ID represented the publication, whereas MaterialPlatform nodes represented the engineered system evaluated within that record. A study could therefore support several relation types for one platform without those relations being transferred to another study. Composition nodes were restricted to constituents of the platform; fabrication nodes to manufacturing or processing operations; cargo nodes to agents actually loaded or carried; and biological nodes to cells, mechanisms, disease models, endpoints, or validation settings tested by the study. Long methods sentences, assay components, culture reagents, antibodies, downstream expression products, and reference-list content were excluded from entity names. This typing rendered relation objects suitable for graph traversal instead of treating the extracted prose as nodes. The operational definitions and principal semantic boundaries of the core relations are summarized in Table 1.

**TABLE 1:** Operational definitions and semantic criteria for core SenescenceBioKG relations.

Relation	Source entity	Target entity	Criterion for Supported	Key exclusion or semantic boundary
Uses	MaterialPlatform	DesignMode	An explicit engineering design mode or organizing design concept is used for the material platform	General material descriptions lacking a defined platform-level engineering design mode are excluded
Composed of	MaterialPlatform	MaterialComposition	A constituent is explicitly incorporated into the composition or structure of the engineered material platform	Culture media, assay reagents, antibodies, downstream molecules, and unrelated method components are excluded
Fabricated by	MaterialPlatform	FabricationStrategy	A manufacturing, processing, assembly, or surface-engineering operation is directly used to produce the platform	Analytical or characterization procedures that do not manufacture or modify the platform are excluded
Responds to	MaterialPlatform	Stimulus	A defined stimulus directly changes material behavior, including cleavage, degradation, swelling, phase, mechanics, permeability, signal, or release	Cellular ROS reduction, antioxidant activity, ordinary testing, untriggered sustained release, and terminology alone are excluded
Delivers	MaterialPlatform	TherapeuticCargo	The platform explicitly carries or delivers an incorporated therapeutic cargo in the study system	Downstream proteins, assay molecules, endogenous readouts, and compounds not incorporated as cargo are excluded
Releases	MaterialPlatform	TherapeuticCargo	Study-specific experimental or descriptive evidence shows cargo release from the material platform	Cargo loading, presence, or carriage without direct release evidence does not qualify
Targets	MaterialPlatform	TargetCell	A cell population is directly tested or experimentally engaged by the material platform in the study	Hypothesized target cells without study-specific experimental evidence are excluded
Modulates	MaterialPlatform	Mechanism	Study-specific experimental evidence supports modulation of the defined biological mechanism, pathway, or molecular node	Background discussion, cited mechanisms, correlations alone, and unsupported pathway interpretations are excluded
Promotes	MaterialPlatform	RegenerativeEndpoint	Study-specific evidence supports the defined tissue-repair or regenerative endpoint associated with the material platform	An isolated molecular-marker change without a qualifying repair or regenerative endpoint is excluded
Validated at	MaterialPlatform	ValidationStage	The reported experimental setting satisfies the operational definition of the assigned validation category	Material source species cannot establish animal validation, and human-derived samples cannot establish clinical validation

ACTIVATES and SUPPRESSES were ontology-defined relations but had no supported instances in the frozen graph. Human-derived evidence is distinct from Clinical validation.

### 2.3 Full-text adjudication and evidence provenance

Each candidate edge was classified as SUPPORTED, REJECTED, or UNCLEAR. SUPPORTED required direct evidence from the same Record ID in the Abstract, Methods, Results, Figure legend, Table, or accessible Supplement. REJECTED indicated conflict with full text, violation of relation semantics, or reliance on an invalid source. UNCLEAR was retained when the available text did not resolve the relation. Only SUPPORTED edges entered the core graph.

The initial extraction produced 3,351 candidate relations: 1,669 SUPPORTED, 1,234 REJECTED, and 448 UNCLEAR. PDF discrepancy correction added 44 supported relations, yielding 1,713 pre-readjudication core edges. A subsequent semantic review revised the candidate universe through the addition of previously omitted relations and the removal or correction of invalid or misassigned candidates. Therefore, the final adjudicated universe comprised 3,403 relations, including 1,868 SUPPORTED, 1,202 REJECTED, and 333 UNCLEAR relations (Supplementary Table S4). Accordingly, the increase from 1,713 to 1,868 supported edges (+155) represents the net result of these revisions rather than 155 simple promotions.

Each core edge received one Evidence ID linked to its Edge ID, Record ID, DOI, source category, and PDF location and an excerpt of no more than 25 words. Evidence strength was coded at the edge level as High, Medium, or Low using relation-specific criteria for the directness of supporting evidence. As evidentiary requirements differed across relation types, these categories were interpreted within each relation family rather than as a common cross-relation scale and did not represent overall study quality. Relation-specific criteria and representative examples are provided in Supplementary Table S10. The final graph and evidence table has a one-to-one Edge ID–Evidence ID correspondence.

Adjudication proceeded at the edge level, rather than through acceptance or rejection of an entire article. Each relation was checked against the corresponding full text and the allowed subject and object types. Suspected cross-paper contamination, methods text in entity fields, implausible cargo assignments, and disagreements between structured fields and evidence were returned to their relevant PDF location. When direct evidence established a corrected target, the derived graph and discrepancy record retained the correction. When the study did not resolve the claim, the edge remained UNCLEAR. Final hygiene checks required unique node, edge, and evidence identifiers; valid relation labels; resolvable source and target nodes; one evidence record per supported edge; and no replacement characters or unreviewed long prose objects.

### 2.4 Relation-specific semantic criteria

RESPONDS TO required a defined stimulus that directly altered material or platform behavior, such as bond or crosslink cleavage, degradation, swelling, phase transition, shape or mechanical change, signal output, permeability, or release. In contrast, cellular reactive-oxygen-species reduction, antioxidant activity, residence in a high-reactive-oxygen-species environment, ordinary mechanical testing, sustained release without a trigger, and responsive naming alone were insufficient.

VALIDATED AT used Cell, *Ex vivo*, Organoid/chip, Small Animal, Large Animal, Human-derived, and Clinical categories. Small Animal and Large Animal denoted animal *in vivo* experiments. Human-derived denoted patient-derived cells, tissues, or samples and was independent from Clinical. Clinical required administration of the material intervention to human participants with reported outcomes. Material source species did not determine validation category (Supplementary Table S5).

### 2.5 Graph, module, and validation-gap analyses

The final directed graph contained canonical typed nodes and evidence-linked edges. Degree, supporting-study count, and relation-type count were calculated as descriptive connectivity measures. Network summaries included typed edge counts, unique directed source–target pairs, weakly connected components, isolated nodes, and relation distributions.

Multi-relation modules required the same Record ID and MaterialPlatform Source ID. Four modules were evaluated: response–release, response–target–regeneration, response–mechanism–regeneration, and target–mechanism–regeneration. They required RESPONDS TO plus RELEASES; RESPONDS TO, TARGETS, and PROMOTES; RESPONDS TO, MODULATES, and PROMOTES; or TARGETS, MODULATES, and PROMOTES, respectively. The same-record co-support did not establish causality.

Three classes of validated-core gap were defined. Type A contained RESPONDS TO and TARGETS without a SUPPORTED PROMOTES edge. Type B contained TARGETS and MODULATES without PROMOTES. Type C contained DELIVERS and PROMOTES without MODULATES. These classes denote absence from the supported graph, not proof that the source study omitted an experiment.

### 2.6 Competency queries, explorer, and reproducibility

Fifteen competency questions covered material–stimulus, material–cargo, target-cell, mechanism, endpoint, validation, relation-diversity, and multi-relation retrieval (Supplementary Table S9). Method A used adjacency-list graph traversal, whereas method B independently used dataframe joins, set operations, and grouped filters without calling graph-traversal functions. The results were compared at the Record ID plus canonical MaterialPlatform Source ID level using precision, recall, F1, and exact-set match. These metrics evaluated implementation agreement, rather than biomedical accuracy.

A read-only R Shiny Explorer loaded canonical nodes, supported edges, public or local evidence fields, and final query results into memory. It provided material, relation, entity, validation, and Record ID filters; material-centered graph views; edge details; Evidence ID retrieval; and the 15 competency queries. Quality control used frozen-input hashes, duplicate- and missing-ID checks, evidence-linkage checks, semantic-edge checks, Unicode scans, and two-run determinism tests. Figure generation and the Explorer used R 4.5.3. Release-level quality control was performed using Python 3.12.13 and Pandas 3.0.1.

The canonical graph data, public evidence metadata, competency-query definitions and results, analysis and figure-generation scripts, and source code of the SenescenceBioKG Explorer are publicly available in the SenescenceBioKG GitHub repository (https://github.com/123lloo11/SenescenceBioKG).

## 3 Results

### 3.1 Full-text curation produced a traceable SenescenceBioKG

Full-text curation transformed 283 original studies into a material-centered evidence graph. The 3,351 initial candidates comprised 1,669 supported, 1,234 rejected, and 448 unclear relations. PDF discrepancy correction added 44 supported relations and yielded 1,713 pre-readjudication edges. Targeted relation review and semantic correction produced the final 1,868 supported edges (Figure 1A). The +155 net change incorporates additions and removals across later audits.

**FIGURE 1:**
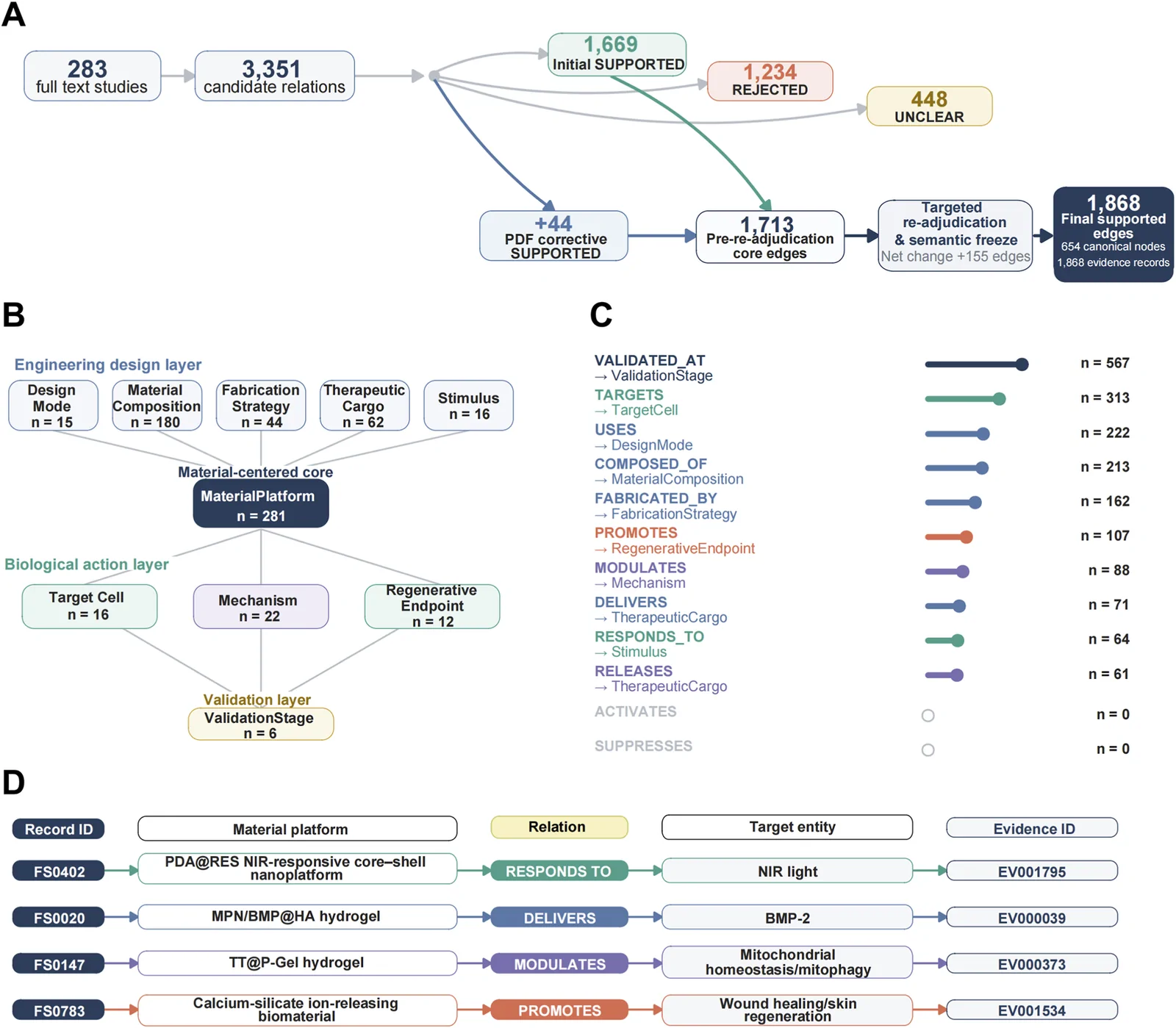
Construction, ontology structure, and evidence provenance of SenescenceBioKG. **(A)** Relation-level validation began with 3,351 candidates from 283 studies. The initial partition was 1,669 SUPPORTED, 1,234 REJECTED, and 448 UNCLEAR; REJECTED and UNCLEAR candidates did not enter the core. The PDF discrepancy review added 44 supported relations, yielding 1,713 pre-re-adjudication edges. Targeted re-adjudication and semantic freeze produced 1,868 final supported edges, 654 canonical nodes, and 1,868 evidence records. The +155 net change is arithmetic and is not a promoted-edge count. **(B)** Fourteen entity types were defined, of which 10 were instantiated in the frozen graph. Clinical was defined within ValidationStage but had zero instances. **(C)** Domain–relation–range structure and supported-edge composition. ACTIVATES and SUPPRESSES were defined but had zero edges. **(D)** Four same-record chains link Record ID, MaterialPlatform, relation, canonical target, and Evidence ID. Every core edge has linked same-record evidence; verbatim excerpts and locations are retained outside the main figure.

The graph contained 654 canonical nodes and 1,868 evidence records. MaterialPlatform was the largest class with 281 nodes, followed by MaterialComposition and TherapeuticCargo with 180 and 62 nodes, respectively. Fourteen entity types were defined and 10 were instantiated (Figures 1B,C). Every core edge retained a chain from Record ID through material, relation, target entity, and Evidence ID. Figure 1D presents four representative same-record provenance chains; unsupported and unresolved candidates remained outside the core.

The final relation composition covered both engineering and biological evidence. VALIDATED AT contributed 567 edges; TARGETS, 313; USES, 222; COMPOSED OF, 213; FABRICATED BY, 162; PROMOTES, 107; MODULATES, 88; DELIVERS, 71; RESPONDS TO, 64; and RELEASES, 61. The distribution shows that the graph does not equate the presence of a material with a complete biological evidence chain. A platform could have detailed construction and validation information yet lack a supported mechanism or endpoint, whereas another could contribute several cell, mechanism, and regeneration relations. The evidence-linked representation preserved both patterns without imputing missing relations.

### 3.2 Relation-level adjudication separated supported and unsupported claims

The closed final adjudicated universe contained 3,403 relation instances: 1,868 SUPPORTED, 1,202 REJECTED, and 333 UNCLEAR. Support rates differed by relation. RESPONDS TO and VALIDATED AT reached 90.1% and 82.1%, respectively, whereas RELEASES, DELIVERS, MODULATES, and PROMOTES ranged from 29.5% to 37.7% (Figures 2A,B). These rates reflect relation-specific evidence requirements and the targeted audits that defined the final universes.

**FIGURE 2:**
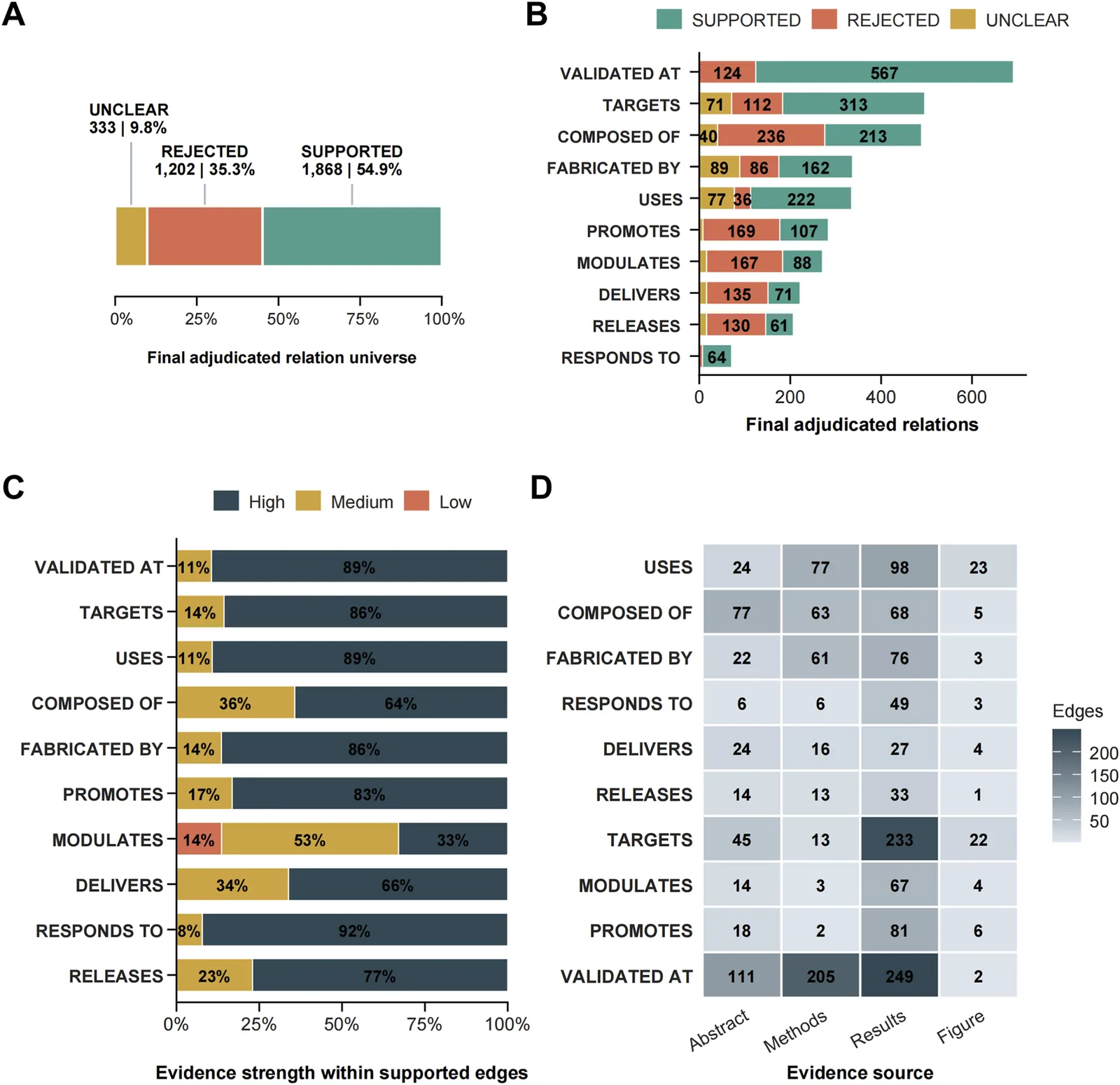
Full-text relation validation across the frozen SenescenceBioKG. **(A)** Composition of the final adjudicated universe, with percentages calculated across final SUPPORTED, REJECTED, and UNCLEAR states. **(B)** Final relation-wise adjudication after targeted full-text semantic audit; relations with zero instances are omitted. **(C)** Evidence-strength distribution among 1,868 supported edges. High, Medium, and Low are edge-level evidence assignments, not study-quality grades. **(D)** Source sections of same-record full-text evidence. Counts are unique Edge ID values.

Evidence strength was High for 1,521 edges (81.4%), Medium for 335 (17.9%), and Low for 12 (0.6%). The Results sections supplied 981 evidence records; Methods, 459; Abstracts, 355; and Figures, 73 (Figures 2C,D). High denotes direct relation-level support, not overall study quality. The final edge set contained no duplicate Node ID, Edge ID, Evidence ID, or semantic edge and no missing source, target, or evidence identifier.

Relation-wise counts clarified where semantic screening had the greatest effect. The final graph retained 213 COMPOSED OF, 162 FABRICATED BY, and 222 USES edges describing platform identity and construction. In contrast, only 71 DELIVERS and 61 RELEASES edges met the distinct cargo and release criteria. Biological support comprised 313 TARGETS, 88 MODULATES, and 107 PROMOTES edges. No ACTIVATES or SUPPRESSES edge remained because supported mechanistic statements were represented under the more conservative MODULATES relation. Therefore, the closed adjudication table documents not only graph size but the relation-specific distance between candidate extraction and directly supported evidence.

### 3.3 Recurrent engineering and biological entities organized the global graph

All 654 nodes formed one weakly connected component containing 1,868 typed evidence edges and 1,821 unique directed source–target pairs (Figure 3A). Material degree varied across studies. The node for the all-in-one smart hydrogel dressing (ASD) had 16 edges spanning eight relation types, whereas the nodes for CCM@CS-HG and Q16 OGPQ/G-NZ each had a degree of 14. These graph-based metrics describe how the materials are represented in the graph, not their therapeutic performance (Figure 3B).

**FIGURE 3:**
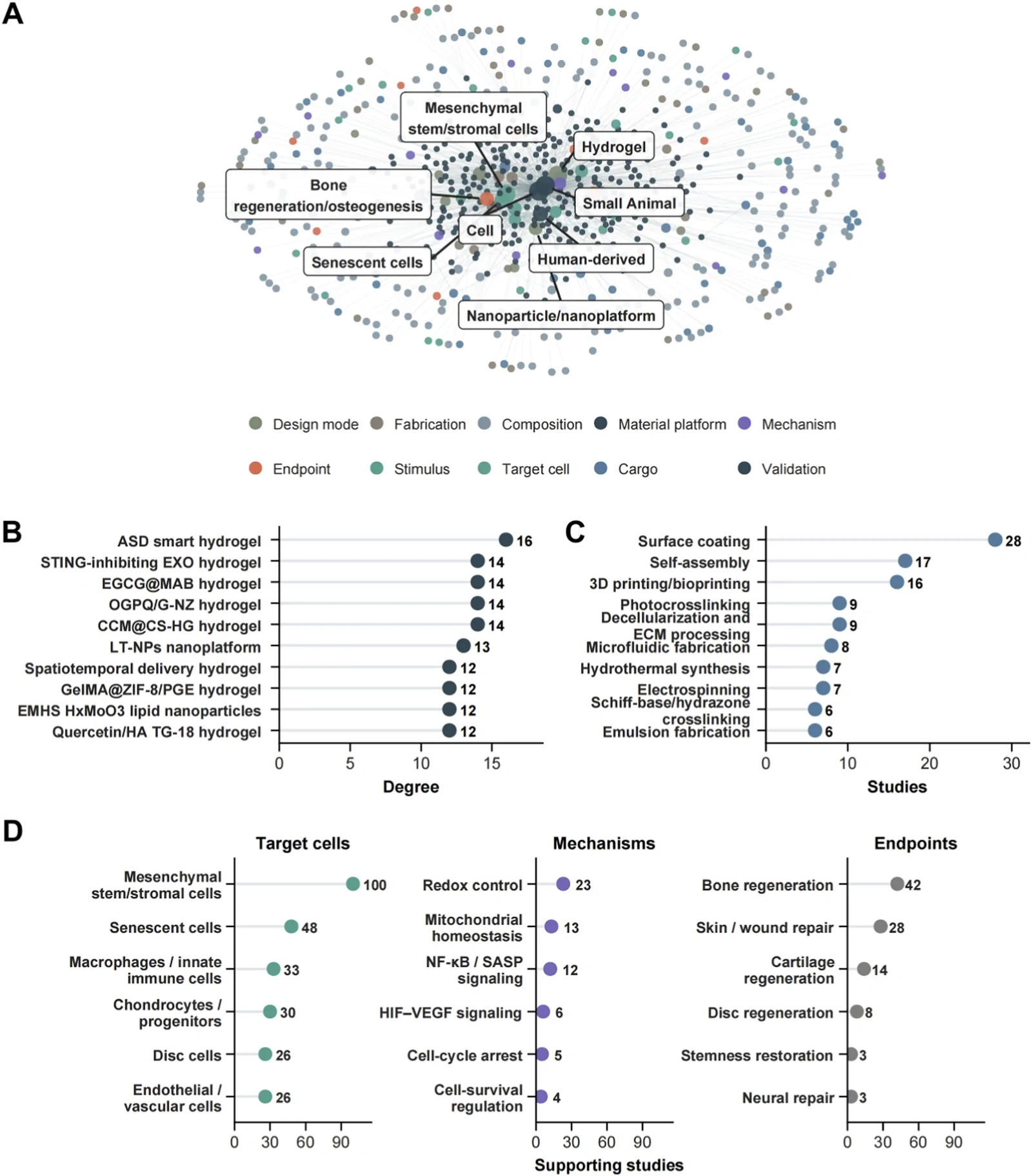
Global structure of the validated SenescenceBioKG. **(A)** Complete typed network of 654 canonical nodes and 1,868 supported edges. Node color denotes entity type, and edge color denotes relation. Hub labels reflect descriptive degree, supporting-study count, and relation-type coverage without implying biological importance. **(B)** MaterialPlatform nodes ranked by degree. **(C)** FabricationStrategy nodes ranked by supporting studies. **(D)** TargetCell, Mechanism, and RegenerativeEndpoint nodes ranked independently by supporting studies.

Fabrication showed a distributed engineering profile. Surface coating occurred in 28 studies, self-assembly in 17, and 3D printing or bioprinting in 16. Decellularization or extracellular-matrix processing and photocrosslinking each occurred in nine studies, followed by microfluidic fabrication in eight and electrospinning in seven (Figure 3C). Therefore, additive manufacturing was well represented but was not the predominant fabrication route.

Mesenchymal stem/stromal cells were targeted in 100 studies, followed by senescent cells in 48, macrophage or innate immune populations in 33, and chondrocytes or cartilage progenitors in 30. Oxidative-stress control, mitochondrial homeostasis or mitophagy, and NF-κB/SASP signaling were the most represented mechanisms. Bone regeneration or osteogenesis led the endpoint categories, followed by wound/skin, cartilage, and intervertebral-disc regeneration (Figure 3D).

The architecture was material-centered rather than dominated by a single biological node. MaterialPlatform nodes linked engineering descriptions to several biological layers, whereas composition, fabrication, cargo, stimulus, cell, mechanism, endpoint, and validation nodes provided typed destinations. The single weakly connected component emerged from shared canonical entities across studies, not from cross-study mechanistic edges. Thus, a common node, such as mesenchymal stem/stromal cells, connected records at the global level, whereas multi-hop study modules were calculated only after reimposing Record ID and MaterialPlatform constraints. This prevented the network layout from creating synthetic paths assembled across unrelated publications.

### 3.4 Explicit responsiveness formed a restricted set of engineering modules

Within the curated corpus, strict responsiveness comprised 64 edges from 55 studies and 55 material platforms. Acidic pH accounted for 22 edges, whereas reactive oxygen species accounted for 18, followed by mechanical force with seven and near-infrared light with three. Ultrasound and mechanical strain contributed two edges each, whereas the remaining stimulus classes contributed one edge each (Figure 4A). Some materials responded to more than one stimulus; thus, edge count exceeded the study and material counts (Supplementary Table S6).

**FIGURE 4:**
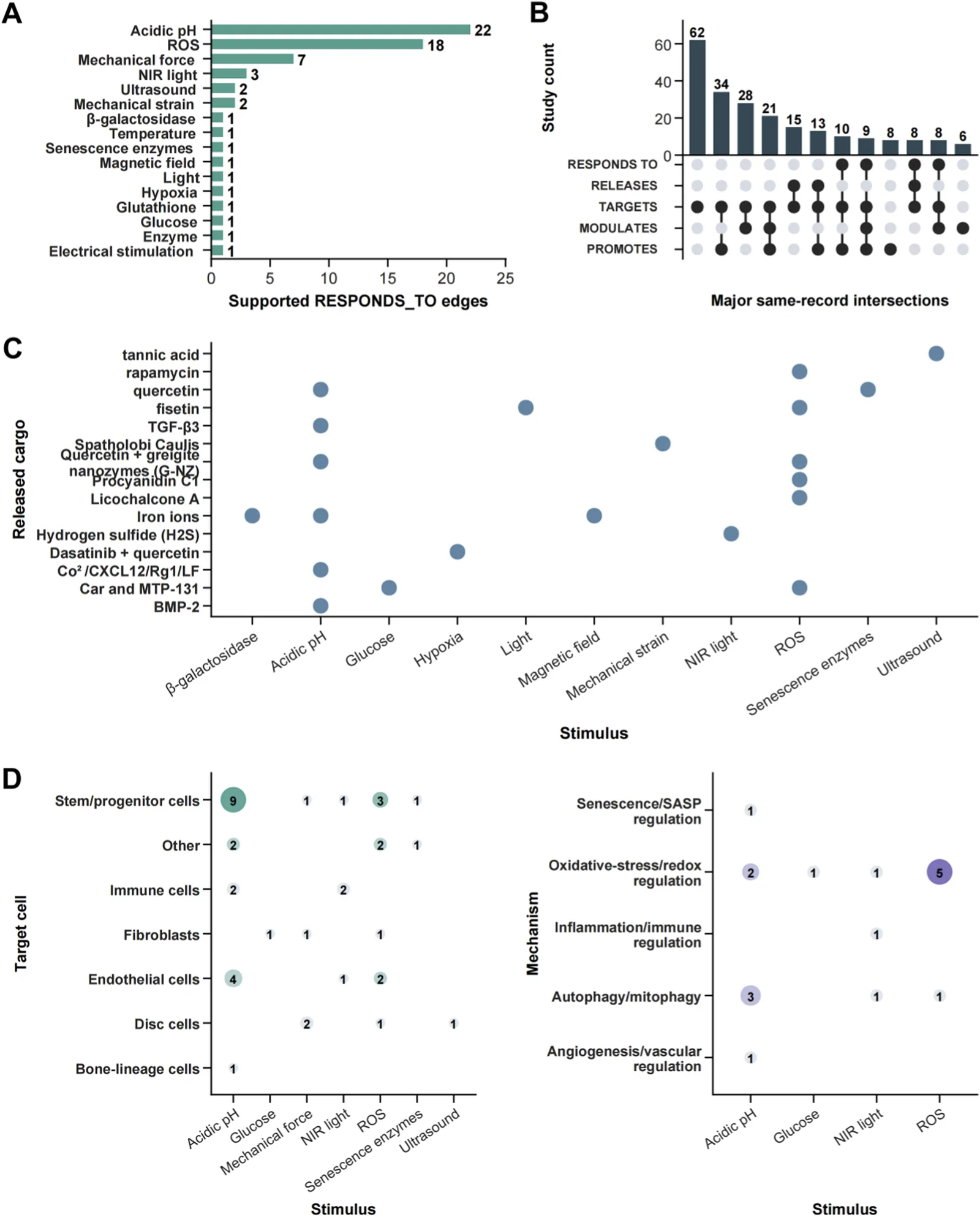
Strict explicit stimulus responsiveness in SenescenceBioKG. **(A)** Supported RESPONDS TO edges by canonical stimulus; edge, study, and material counts are distinct. **(B)** Same-Record ID and same-MaterialPlatform intersections among RESPONDS TO, RELEASES, TARGETS, MODULATES, and PROMOTES. **(C)** Stimulus and released-cargo combinations in systems with co-supported RESPONDS TO and RELEASES edges. Co-support does not establish stimulus-triggered release unless that dependency is directly demonstrated. **(D)** Same-record response–target–regeneration and response–mechanism–regeneration modules. Modules are co-supported evidence configurations, not causal chains.

Seventeen study/material pairs had co-supported RESPONDS TO and RELEASES edges. Twenty-four combined RESPONDS TO, TARGETS, and PROMOTES, whereas 12 combined RESPONDS TO, MODULATES, and PROMOTES (Figures 4B–D). These intersections link material behavior to release, tested cells, mechanisms, or regenerative endpoints within one record. Response and release co-support was not labeled stimulus-triggered release unless the dependency was directly demonstrated.

The difference between 64 response edges and 55 responsive studies reflected multi-stimulus systems, rather than duplicate counting. Each response edge paired a canonical MaterialPlatform with a distinct canonical stimulus and retained its own evidence record. Therefore, counts were reported separately as edges, studies, and materials. The 24 response–target–regeneration modules outnumbered the 12 response–mechanism–regeneration modules, indicating that responsive platforms more often had co-supported cell and endpoint relations than an explicit mechanism relation in the validated core. This is an evidence-coverage comparison; therefore, it does not imply that the remaining studies lacked a biological mechanism.

### 3.5 Cell–mechanism–regeneration patterns recurred within studies

The alluvial analysis contained 51 target-cell–mechanism–endpoint paths from 35 Record IDs. Thirty-five study/material pairs contained the complete TARGETS, MODULATES, and PROMOTES module (Figures 5A,B; Supplementary Table S7). Multiple paths occurred when one record supported several cells, mechanisms, or endpoints.

**FIGURE 5:**
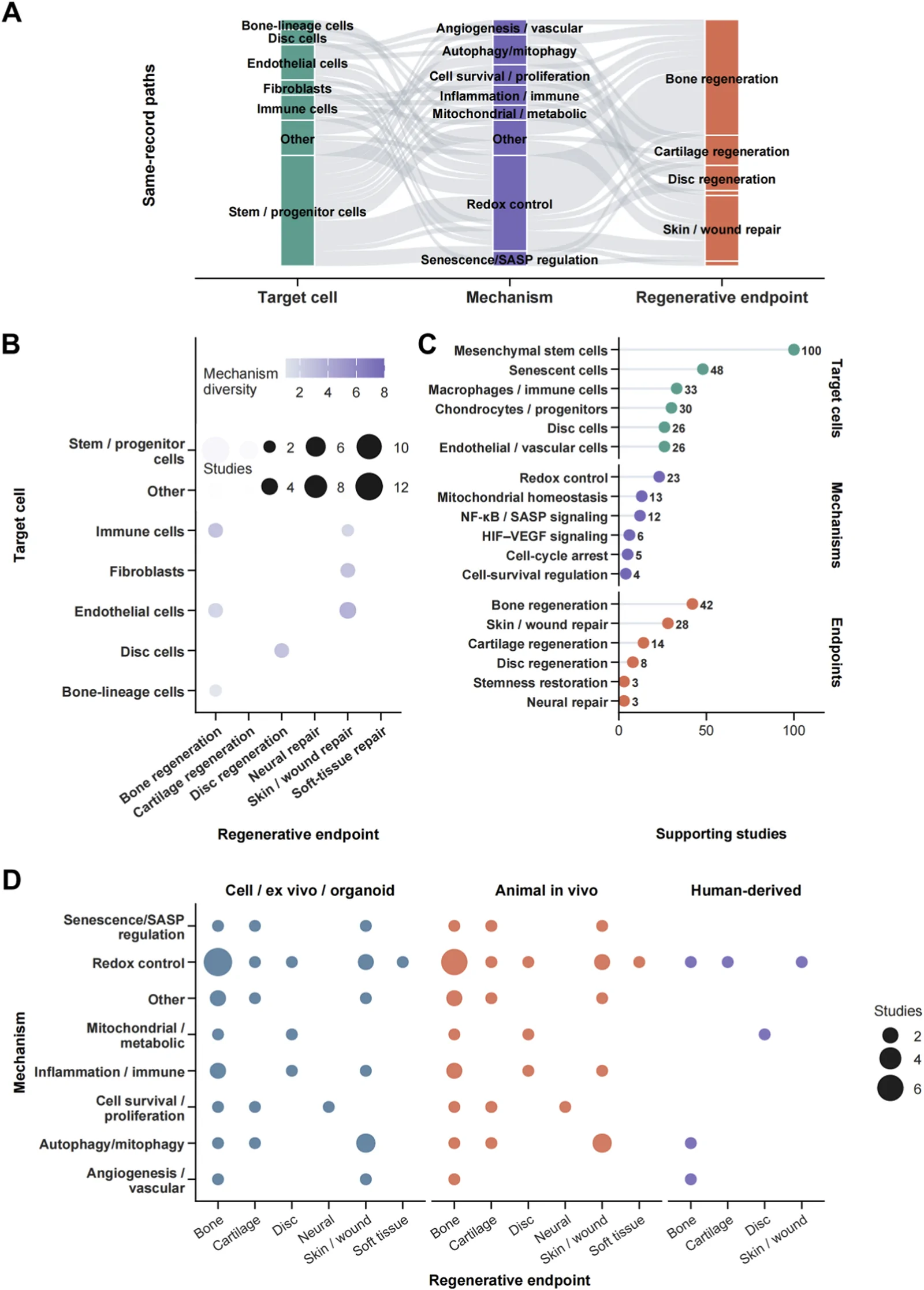
Within-study cell–mechanism–regeneration evidence structure. **(A)** TargetCell–Mechanism–RegenerativeEndpoint paths supported within the same Record ID and MaterialPlatform; display grouping reduces label fragmentation. **(B)** Target cell by regenerative-endpoint modules, with point size indicating supporting studies and color indicating mechanism-group diversity. **(C)** Leading target cells, mechanisms, and regenerative endpoints ranked independently by supporting studies. **(D)** Mechanism–endpoint modules with same-record validation evidence. Animal *in vivo* denotes Small Animal or Large Animal; Human-derived is separate from Clinical. Co-supported paths do not establish causal mediation.

Mesenchymal stem/stromal cells, senescent cells, macrophage or innate immune populations, cartilage-lineage cells, vascular cells, and disc cells were the leading targets. Oxidative-stress control appeared in 23 studies, mitochondrial homeostasis or mitophagy in 13, and NF-κB/SASP signaling in 12. Bone regeneration or osteogenesis was supported in 42 studies, wound/skin regeneration in 28, cartilage regeneration or chondroprotection in 14, and disc regeneration in 8 (Figure 5C). Mechanism–endpoint combinations occurred across cell, animal *in vivo*, and human-derived settings (Figure 5D); however, their same-record occurrence did not demonstrate mediation.

Path construction preserved Evidence ID attached to every constituent relation. A displayed target-cell–mechanism–endpoint path therefore required the TARGETS, MODULATES, and PROMOTES edges from the same record and material, rather than proximity in the global network. The 51 paths exceeded the 35 records because platforms could target more than one cell class, modulate more than one mechanism, or support more than one endpoint. These combinations expose records with comparatively complete cross-layer representation and allow the evidence for each layer to be inspected independently. They do not collapse distinct experiments into one mechanistic claim.

### 3.6 Validation was concentrated in cell and small-animal studies

Cell validation was present in 280 studies and Small Animal validation in 217. Human-derived evidence occurred in 54 studies. *Ex vivo* and Organoid/chip validation each occurred in six studies, Large Animal validation in four, and Clinical validation in none (Figure 6A). One record could support multiple categories, and Human-derived was not treated as a clinical intervention.

**FIGURE 6:**
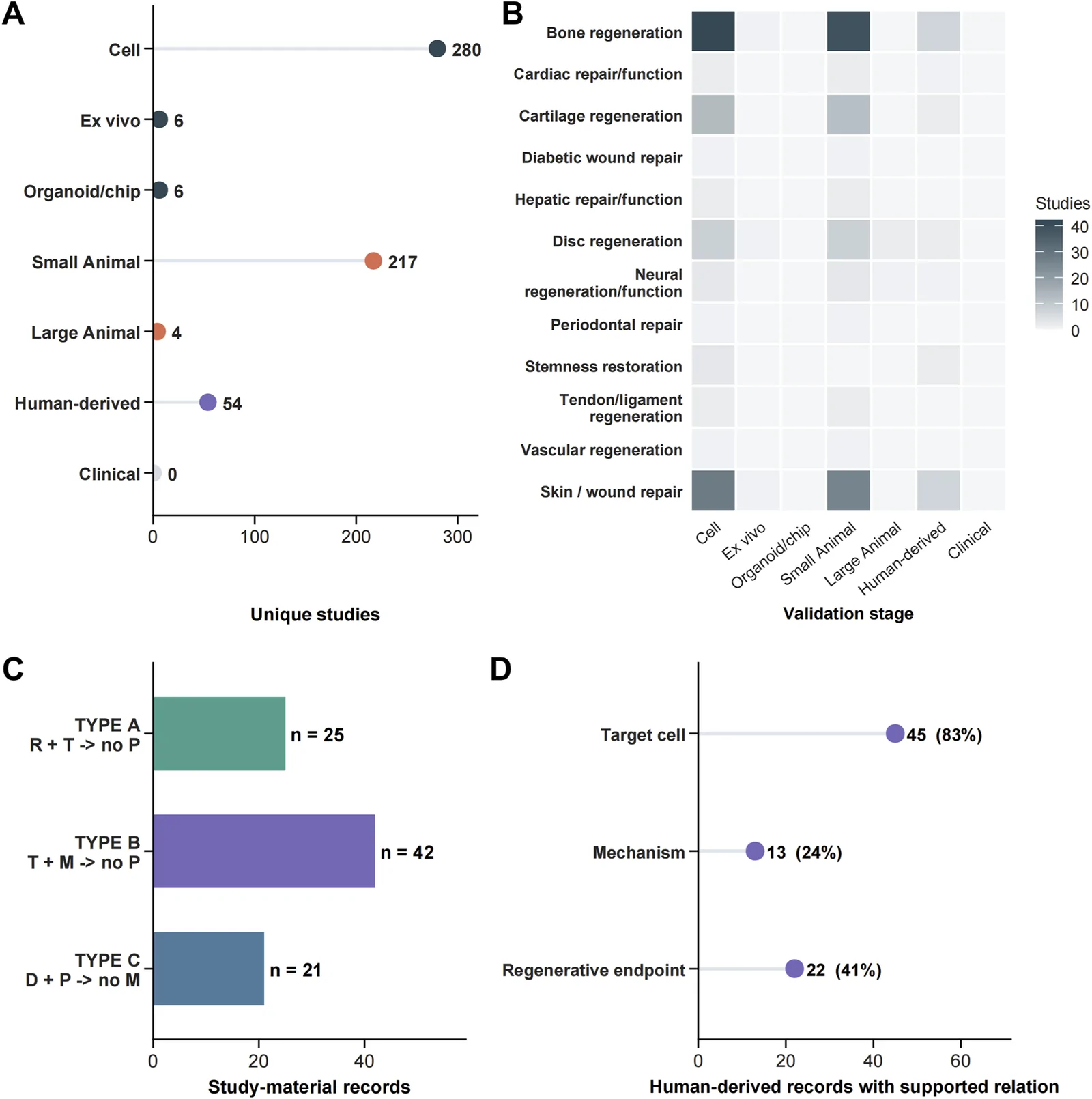
Validation coverage and discontinuities in the validated core. **(A)** Unique-study counts across Cell, *Ex vivo*, Organoid/chip, Small Animal, Large Animal, Human-derived, and Clinical validation. Clinical remains zero. **(B)** RegenerativeEndpoint by ValidationStage matrix. **(C)** Three validated-core gap classes: RESPONDS TO plus TARGETS without SUPPORTED PROMOTES, TARGETS plus MODULATES without SUPPORTED PROMOTES, and DELIVERS plus PROMOTES without SUPPORTED MODULATES. A missing supported edge does not prove that the study omitted an experiment. **(D)** Supported target-cell, mechanism, and endpoint coverage among 54 Human-derived records; Human-derived is not clinical intervention.

Endpoint-specific coverage followed this distribution. Animal *in vivo* validation was present in 39 of the 42 bone-regeneration records, 26 of the 28 wound/skin records, and 12 of the 14 cartilage records (Figure 6B). Nevertheless, the graph nevertheless contained few Organoid/chip and Large-Animal records and no PROMOTES-linked Clinical evidence.

Validated-core gaps included 25 Type A, 42 Type B, and 21 Type C memberships (Supplementary Table S8). The 88 memberships represented 77 unique records because 11 records occurred in two classes (Figure 6C). Human-derived records retained their cell, mechanism, and endpoint relations for separate inspection (Figure 6D). A gap indicates an absent SUPPORTED edge, not the confirmed absence of an experiment from the publication.

Validation edges were counted independently of endpoint edges, and studies were deduplicated within each category. A record with both Cell and Small Animal experiments contributed once to each stage, whereas a patient-derived cell experiment contributed to Human-derived evidence without being promoted to Clinical. The four Large Animal records represented intervention in living large-animal models rather than the use of porcine, bovine, or ovine material sources. These rules produced a map in which extensive cell and small-animal coverage coexisted with specific translational discontinuities. The absence of Clinical edges is a property of this corpus under the operational definition and not a statement regarding all senescence-oriented biomaterial research.

### 3.7 Competency queries reproduced graph retrieval and enabled interactive inspection

All 15 competency questions returned identical Record ID and MaterialPlatform sets under graph traversal and the independent dataframe implementation. Cross-implementation comparison yielded macro precision, recall, and F1 values of 1.00, with exact-set agreement for all 15 queries (Figures 7A,B). These results confirm consistent implementation over the same frozen graph.

**FIGURE 7:**
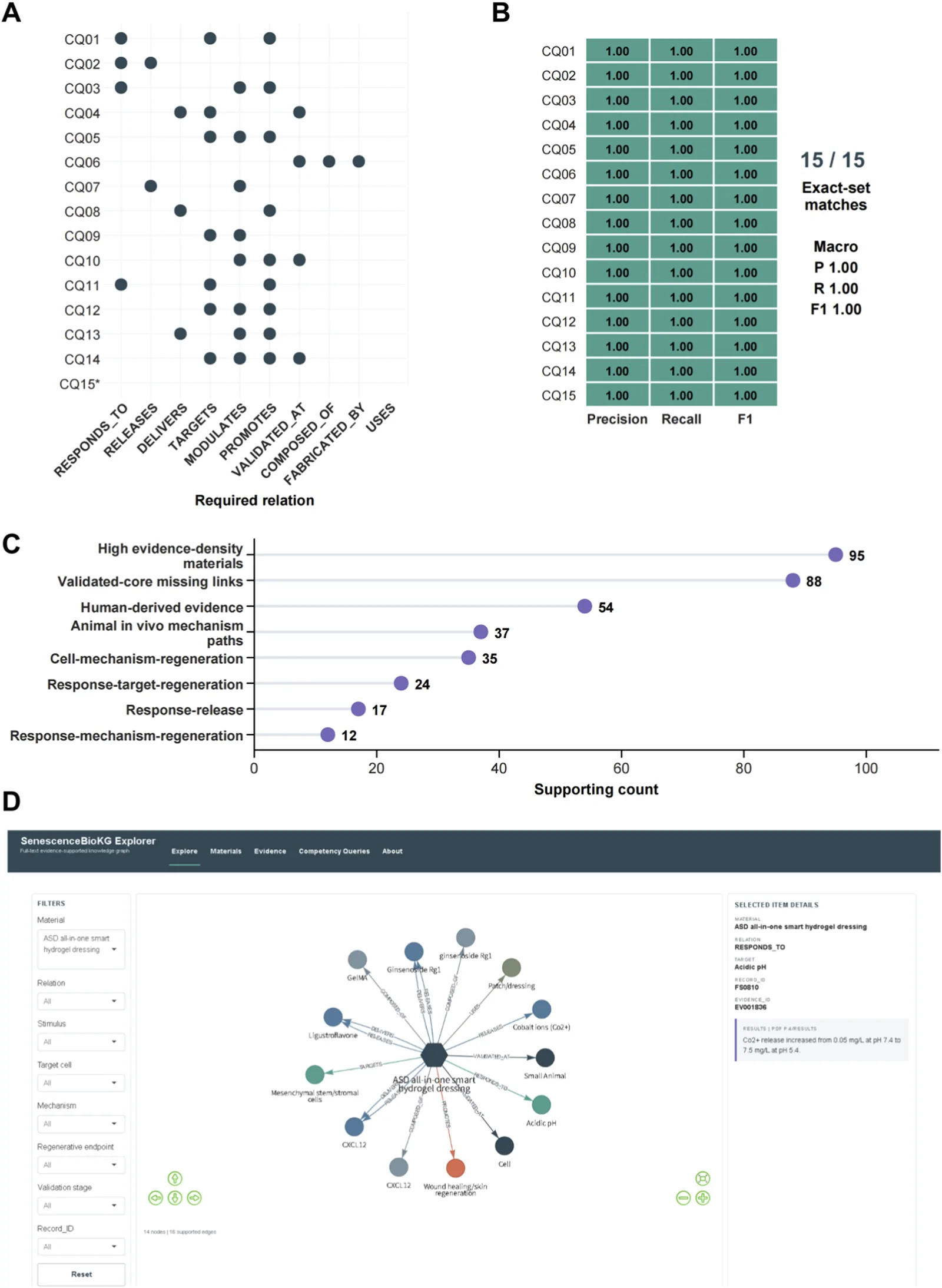
Competency queries and interactive access to SenescenceBioKG.** (A)** Required-relation matrix for the 15 competency questions (CQ01–CQ15); CQ15 uses a relation diversity threshold **(B)** Agreement between graph traversal and an independent dataframe computation. Precision, recall, and F1 quantify implementation agreement, not biomedical truth. **(C)** Selected graph-derived result categories reported as supporting study/material counts. Missing-link counts describe absence from the validated core, not absence of experiments. Animal *in vivo* denotes Small Animal or Large Animal. **(D)** Interface of the locally running, read-only Explorer, showing a material-centered subgraph and linked same-record evidence.

Queries recovered 24 response–target–regeneration pairs, 17 response–release pairs, 12 response–mechanism–regeneration pairs, and 35 target–mechanism–regeneration pairs. Thirty-seven mechanism–regeneration pairs also had Small Animal or Large Animal validation. Four records had Large Animal edges, 54 had Human-derived edges, and 95 study/material pairs had at least eight supported edges across at least five relation types (Figure 7C). Evidence density measures relation coverage rather than material quality.

The local Explorer displayed material-centered subgraphs and returned Record ID, Edge ID, Evidence ID, source, location, and locally available evidence details for selected edges (Figure 7D). Furthermore, it ran the 15 frozen competency queries. The application provides research access to the graph and is not a clinical decision-support system.

The query set tested more than frequency retrieval. It required intersections such as responsive materials with target cells and endpoints, cargo-delivering materials with target cells and animal *in vivo* validation, and mechanism–regeneration paths stratified by validation setting. As both implementations operated on canonical identifiers, their agreement also checked that display synonyms did not split result sets. The benchmark did not adopt an external gold standard; it verified that graph traversal and independently written relational operations implemented the same formal questions over the frozen edge table. The Explorer exposed results as evidence-addressable study/material records rather than untraceable aggregate counts.

## 4 Discussion

SenescenceBioKG changes the unit of analysis from a paper-level label to a full-text-supported relation. This distinction allowed material composition, fabrication, stimulus response, delivery, target cells, mechanisms, regeneration, and validation to be queried separately and then combined within the same study. The frozen graph captured 1,868 supported edges from 283 studies with one Evidence ID per edge. Its main scientific results were a restricted responsive-material subset, recurring cell–mechanism–regeneration configurations, diverse fabrication routes, and uneven validation coverage. This unit of analysis also changes what can be audited. A paper-level label can indicate that an article concerns a hydrogel, senescence, and bone repair; however, it cannot determine whether the hydrogel carried the reported agent, which cells were directly tested, or whether a pathway was measured in the same experiment. Typed biomedical knowledge graphs have previously enabled structured integration and query of heterogeneous biomedical evidence (Humayun et al., 2020; Chandak et al., 2023). Edge-level provenance exposes those distinctions without requiring every downstream user to reconstruct the extraction. It also permits conservative reuse: an analysis may be restricted to selected relations, evidence strengths, source sections, or validation stages while leaving the frozen graph unchanged. For an interdisciplinary field in which material engineering and biological interpretation are often reported in different sections, this granularity is a practical advantage.

Relation-level review is necessary because material claims require different forms of support. Composition and fabrication are commonly documented in the Methods, whereas delivery, release, targeting, mechanism, and regeneration require experimental evidence. Pathway discussion is not pathway testing, cargo loading is not evidence of release, and lower senescence-marker expression is not evidence of selective senescent-cell elimination (Di Micco et al., 2021; Ogrodnik et al., 2024). Full-text adjudication kept such mismatches outside the supported graph. Canonical entities and same-record provenance localize any disagreement to an edge, its source, and its PDF location. Therefore, relation-specific support rates reflect semantic stringency, not study quality. Lower rates for release, delivery, mechanism, and regeneration partly arose from passive release labeled as responsiveness, downstream proteins labeled as cargo, or cited pathway language labeled as measured mechanism. Rejected and unclear candidates remain auditable outside the core; evidence location is part of that distinction. A Methods passage can establish composition or fabrication, whereas a Results statement, Figure, or Table is usually needed for release, targeting, mechanism, or regeneration. The source annotation enables rapid return to the relevant experiment and reduces reliance on an isolated extracted sentence. It also permits later users to apply stricter source or evidence-strength filters without changing the accepted edge set.

Smart-material literature defines responsiveness as a measurable material change produced by a specified input (Badeau and DeForest, 2019; Neumann et al., 2023; Liao et al., 2025). Applying that criterion within the curated corpus yielded 64 RESPONDS TO edges across 55 studies and 55 material platforms. The predominance of acidic pH and reactive oxygen species suggests that responsive designs have preferentially exploited biochemical cues associated with stressed tissue microenvironments. These stimuli are also readily coupled to degradable, cleavable, or release-regulating material chemistries, which may partly explain their greater representation than mechanical, optical, or other triggers. Acidic pH and reactive oxygen species supplied most edges, with smaller groups for mechanical, optical, enzymatic, magnetic, metabolic, and thermal cues. Same-record analysis identified 17 response–release, 24 response–target–regeneration, and 12 response–mechanism–regeneration combinations. Direct comparison of stimulated and unstimulated material behaviors remains necessary for a triggered-release claim (Wei et al., 2022). This boundary separates smart-material characterization from antioxidant activity, ordinary controlled release, or responsive terminology without a measured material change. The restricted definition retains systems with an observable change in material behavior, excluding studies in which a material merely operates under a stressful environment or reduces a biological stimulus. The resulting 55-study set is smaller than a keyword-defined responsive corpus; however, its edges are directly inspectable. The multi-relation modules show where engineering-response evidence is accompanied by cellular or regenerative observations and where a supported mechanism layer is less common. The cell–mechanism–regeneration results indicate how senescence-oriented biomaterials were tested across distinct intervention logics. Mesenchymal stem/stromal cells were the most frequent, whereas senescent cells, macrophages, vascular cells, chondrocytes, disc cells, and skin-associated cells represented different direct or microenvironmental targets. The recurrence of mesenchymal stem/stromal cells, senescent cells, and macrophages reflects three recurrent experimental roles in this literature, namely, regenerative effector cells, direct targets of senescence-oriented intervention, and immune regulators linking material responses to tissue repair, respectively. Senescent-cell–immune interactions, particularly those involving macrophages, are also closely associated with tissue repair and regenerative regulation (Pajarinen et al., 2019; Elder and Emmerson, 2020; Majewska and Krizhanovsky, 2025). Oxidative-stress control, mitochondrial homeostasis, and NF-κB/SASP signaling frequently accompanied bone, wound/skin, cartilage, and disc endpoints. The lower representation of MODULATES than TARGETS also suggests that cellular engagement was more frequently documented than mechanistic closure, which required more specific pathway-level experimental support. Reduced p16, p21, senescence-associated β-galactosidase, or inflammatory markers cannot establish senolysis without selective elimination evidence (Ogrodnik et al., 2024). A platform centered on senescent-cell elimination, one protecting progenitor cells from stress and one remodeling an inflammatory microenvironment, can contribute valid but different patterns. Canonical TargetCell and Mechanism nodes make them comparable without forcing an identical mode of action. The one-edge–one-evidence model also distinguishes a mechanism measured in cells from an endpoint observed in animals. Fabrication connects the resource to smart materials and additive manufacturing while remaining a separate evidence layer. Surface coating and self-assembly were the most frequent, whereas 3D printing or bioprinting appeared in 16 studies. Photocrosslinking, extracellular-matrix processing, microfluidics, and electrospinning provided other routes to control interfaces, geometry, porosity, and carrier structure. Several processes can coexist in one platform, including printed constructs that also undergo responsive biofabrication or contain processed extracellular matrix. Printing fidelity, crosslinking, and structural characterization remain separate from responsiveness and functional recovery. A fabrication edge establishes how a platform was made; however, it cannot by itself establish cargo release, cellular targeting, or tissue function. The distributed fabrication profile also indicates that additive manufacturing is one route among several, not the organizing principle of the corpus. Three- and four-dimensional bioprinting can combine geometry, multiple materials, and time-dependent behavior, whereas decellularized extracellular matrix provides tissue-derived biochemical and structural cues (Gao et al., 2023; Liu et al., 2025). These approaches remain comparable in the graph as FABRICATED BY records construction, whereas other relations record response, delivery, mechanism, and endpoint evidence separately.

Validation was concentrated in Cell and Small-Animal experiments. Human-derived evidence was more frequent than *Ex vivo*, Organoid/chip, or Large-Animal evidence but comprised cells, tissues, and samples rather than intervention in participants. Clinical remained zero under the defined criterion. The sparse Organoid/chip and Large-Animal categories identify specific evidence needs rather than positions on a linear maturity scale. Organoid and chip systems can examine multicellular interactions under controlled conditions, whereas Large-Animal studies address scale, implantation, mechanics, and delivery (Gonzalez Vazquez et al., 2021; Li et al., 2024). The gap classes provide a practical way to identify records with incomplete supported evidence chains, rather than indicating that the corresponding experiments were absent from the source studies. The validation profile also cautions against a single maturity score: categories address different questions, and a record may support several categories. Stage-specific counts and multiple VALIDATED AT edges reveal the strong Small-Animal base while preserving the scarcity of Organoid/chip and Large-Animal studies and preventing Human-derived evidence from being presented as clinical testing.

Several limitations constrain the interpretation of the findings. SenescenceBioKG reflects a predefined search, fixed 283-study corpus, and predefined ontology, whereas full-text adjudication involved scientific judgment without formal interrater agreement. Same-record co-support does not establish causal mediation, and missing edges, node degree, or edge counts should not be interpreted as absent experiments, biological importance, effect estimates, or intervention rankings. Human-derived and Clinical evidence were operationally distinct, whereas competency-query metrics assessed implementation agreement rather than biomedical truth. The graph captures evidence presence rather than effect size, cross-laboratory reproducibility, or risk of bias, and future updates should preserve versioning, adjudication history, and provenance.

The competency questions and Explorer add reusable access to the frozen evidence without changing these boundaries. Queries may require responsiveness, fabrication, cargo, cells, mechanisms, endpoints, or animal *in vivo* validation to coexist for one material and Record ID, whereas Evidence IDs support targeted source inspection. Therefore, SenescenceBioKG provides a compact, auditable representation of smart and responsive biomaterial design, cell–mechanism–regeneration evidence, manufacturing strategies, and validation gaps in senescence-oriented tissue engineering. Its value lies in reproducible relation retrieval and explicit provenance. Public release separates reusable graph metadata from copyrighted source passages. Canonical nodes, supported edges, evidence identifiers and locations, query definitions, analytical code, and the Explorer can be distributed, whereas source PDFs and bulk verbatim excerpts remain local. This approach preserves the computational reproducibility of the frozen analyses while directing users to the cited studies for source interpretation.

## Data Availability

The datasets presented in this study can be found in online repositories. The names of the repository/repositories and accession number(s) can be found in the article/Supplementary Material.

## References

[B1] Badeau B. A. DeForest C. A. (2019). Programming stimuli-responsive behavior into biomaterials. Annu. Rev. Biomed. Eng. 21, 241–265. 10.1146/annurev-bioeng-060418-052324 30857392

[B2] Chandak P. Huang K. Zitnik M. (2023). Building a knowledge graph to enable precision medicine. Sci. Data 10 (1), 67. 10.1038/s41597-023-01960-3 36732524 PMC9893183

[B3] Chen A. Wang W. Mao Z. He Y. Chen S. Liu G. (2024). Multimaterial 3D and 4D bioprinting of heterogenous constructs for tissue engineering. Adv. Mater 36 (34), e2307686. 10.1002/adma.202307686 37737521

[B4] Dai H. Fan Q. Wang C. (2022). Recent applications of immunomodulatory biomaterials for disease immunotherapy. Explor. (Beijing) 2 (6), 20210157. 10.1002/EXP.20210157 37324799 PMC10191059

[B5] Demaria M. Ohtani N. Youssef S. A. Rodier F. Toussaint W. Mitchell J. R. (2014). An essential role for senescent cells in optimal wound healing through secretion of PDGF-AA. Dev. Cell 31 (6), 722–733. 10.1016/j.devcel.2014.11.012 25499914 PMC4349629

[B6] Di Micco R. Krizhanovsky V. Baker D. d'Adda di Fagagna F. (2021). Cellular senescence in ageing: from mechanisms to therapeutic opportunities. Nat. Rev. Mol. Cell Biol. 22 (2), 75–95. 10.1038/s41580-020-00314-w 33328614 PMC8344376

[B7] Ebeid I. A. (2022). MedGraph: a semantic biomedical information retrieval framework using knowledge graph embedding for PubMed. Front. Big Data 5, 965619. 10.3389/fdata.2022.965619 36338335 PMC9627348

[B8] Elder S. S. Emmerson E. (2020). Senescent cells and macrophages: key players for regeneration? Open Biol. 10 (12), 200309. 10.1098/rsob.200309 33352064 PMC7776574

[B9] Gao Q. Lee J. S. Kim B. S. Gao G. (2023). Three-dimensional printing of smart constructs using stimuli-responsive biomaterials: a future direction of precision medicine. Int. J. Bioprint 9 (1), 638. 10.18063/ijb.v9i1.638 36636137 PMC9830998

[B10] Gonzalez Vazquez A. G. Blokpoel Ferreras L. A. Bennett K. E. Casey S. M. Brama P. A. O'Brien F. J. (2021). Systematic comparison of biomaterials-based strategies for osteochondral and chondral repair in large animal models. Adv. Healthc. Mater 10 (20), e2100878. 10.1002/adhm.202100878 34405587 PMC11468758

[B11] Han J. Rindone A. N. Elisseeff J. H. (2024). Immunoengineering biomaterials for musculoskeletal tissue repair across lifespan. Adv. Mater 36 (28), e2311646. 10.1002/adma.202311646 38416061 PMC11239302

[B12] Humayun F. Domingo-Fernandez D. Paul George A. A. Hopp M. T. Syllwasschy B. F. Detzel M. S. (2020). A computational approach for mapping heme biology in the context of hemolytic disorders. Front. Bioeng. Biotechnol. 8, 74. 10.3389/fbioe.2020.00074 32211383 PMC7069124

[B13] Li J. Liu Y. Zhang Y. Yao B. Song W. Li Z. (2021). Biophysical and biochemical cues of biomaterials guide mesenchymal stem cell behaviors. Front. Cell Dev. Biol. 9, 640388. 10.3389/fcell.2021.640388 33842464 PMC8027358

[B14] Li Z. Zhong Q. Yang J. Duan Y. Wang W. Wu C. (2022). DeepKG: an end-to-end deep learning-based workflow for biomedical knowledge graph extraction, optimization and applications. Bioinformatics 38 (5), 1477–1479. 10.1093/bioinformatics/btab767 34788369 PMC8689937

[B15] Li X. Sheng S. Li G. Hu Y. Zhou F. Geng Z. (2024). Research progress in hydrogels for cartilage organoids. Adv. Healthc. Mater 13 (22), e2400431. 10.1002/adhm.202400431 38768997

[B16] Liao Z. Liu T. Yao Z. Hu T. Ji X. Yao B. (2025). Harnessing stimuli-responsive biomaterials for advanced biomedical applications. Explor. (Beijing) 5 (1), 20230133. 10.1002/EXP.20230133 40040822 PMC11875454

[B17] Liu J. Song Q. Yin W. Li C. An N. Le Y. (2025). Bioactive scaffolds for tissue engineering: a review of decellularized extracellular matrix applications and innovations. Explor. (Beijing) 5 (1), 20230078. 10.1002/EXP.20230078 40040827 PMC11875452

[B18] Majewska J. Krizhanovsky V. (2025). Immune surveillance of senescent cells in aging and disease. Nat. Aging 5 (8), 1415–1424. 10.1038/s43587-025-00910-5 40813810

[B19] Neumann M. di Marco G. Iudin D. Viola M. van Nostrum C. F. van Ravensteijn B. G. P. (2023). Stimuli-responsive hydrogels: the dynamic smart biomaterials of tomorrow. Macromolecules 56 (21), 8377–8392. 10.1021/acs.macromol.3c00967 38024154 PMC10653276

[B20] Ogrodnik M. Carlos Acosta J. Adams P. D. d'Adda di Fagagna F. Baker D. J. Bishop C. L. (2024). Guidelines for minimal information on cellular senescence experimentation *in vivo* . Cell 187 (16), 4150–4175. 10.1016/j.cell.2024.05.059 39121846 PMC11790242

[B21] Pajarinen J. Lin T. Gibon E. Kohno Y. Maruyama M. Nathan K. (2019). Mesenchymal stem cell-macrophage crosstalk and bone healing. Biomaterials 196, 80–89. 10.1016/j.biomaterials.2017.12.025 29329642 PMC6028312

[B22] Rahimnejad M. Jahangiri S. Zirak Hassan Kiadeh S. Rezvaninejad S. Ahmadi Z. Ahmadi S. (2024). Stimuli-responsive biomaterials: smart avenue toward 4D bioprinting. Crit. Rev. Biotechnol. 44 (5), 860–891. 10.1080/07388551.2023.2213398 37442771

[B23] Rhinn M. Ritschka B. Keyes W. M. (2019). Cellular senescence in development, regeneration and disease. Development 146 (20), dev151837. 10.1242/dev.151837 31575608

[B24] Shvedova M. Samdavid Thanapaul R. J. R. Thompson E. L. Niedernhofer L. J. Roh D. S. (2022). Cellular senescence in aging, tissue repair, and regeneration. Plast. Reconstr. Surg. 150, 4S–11S. 10.1097/PRS.0000000000009667 36170430 PMC9529244

[B25] Wei H. Cui J. Lin K. Xie J. Wang X. (2022). Recent advances in smart stimuli-responsive biomaterials for bone therapeutics and regeneration. Bone Res. 10 (1), 17. 10.1038/s41413-021-00180-y 35197462 PMC8866424

[B26] Wilkinson H. N. Hardman M. J. (2022). Cellular senescence in acute and chronic wound repair. Cold Spring Harb. Perspect. Biol. 14 (11), a041221. 10.1101/cshperspect.a041221 35817510 PMC9620855

[B27] Zhang Y. Xu Y. Kong H. Zhang J. Chan H. F. Wang J. (2023). Microneedle system for tissue engineering and regenerative medicine. Explor. (Beijing) 3 (1), 20210170. 10.1002/EXP.20210170 37323624 PMC10190997

[B28] Zhang Y. Judeh R. Aravind S. Zreiqat H. Lu Z. (2025). Mesenchymal stem cell senescence and biomaterial-based next-generation rejuvenation strategy. Tissue Eng. Part B Rev., 19373341251372962. 10.1177/19373341251372962 40956681

[B29] Zhao Y. Luo Y. Yang J. Wu J. Wang X. (2025). Biomaterial-assisted senescence-targeting treatment strategy for central nervous system injury. Tissue Eng. Part B Rev., 19373341251359272. 10.1177/19373341251359272 40711913

