# Supplementary material for: SenescenceBioKG reveals full-text-supported biomaterial design, mechanisms, and regeneration patterns in senescence-oriented tissue engineering
Source: Front Bioeng Biotechnol. 2026 Oct 29;14:1966523. doi: 10.3389/fbioe.2026.1966523 (PMC13754808; doi:10.3389/fbioe.2026.1966523)
Supplement: Supplementary file 1 [file Table1.docx]

**Supplementary Material**

**SenescenceBioKG reveals full-text-supported biomaterial design, mechanism, and regeneration patterns in senescence-oriented tissue engineering**

**Supplementary Figure S1**

**Supplementary Tables S1-S10**

**Supplementary Figure S1. Study identification and corpus selection workflow**

The vector figure is provided as the accompanying file Supplementary Figure S1.tiff.

**Table S1. Complete database search strategies used for corpus identification**

| **Database** | **Search field** | **Complete search strategy** | **Search date** | **Records retrieved** |
| --- | --- | --- | --- | --- |
| Web of Science Core Collection | Title, abstract, and author keywords (TI, AB, AK) | #1 (TI=(senescen* OR senolytic* OR senomorphic* OR senotherap* OR "senescence-associated secretory phenotype") OR AB=(senescen* OR senolytic* OR senomorphic* OR senotherap* OR "senescence-associated secretory phenotype") OR AK=(senescen* OR senolytic* OR senomorphic* OR senotherap* OR "senescence-associated secretory phenotype"))  #2 (TI=(biomaterial* OR "biocompatible material*" OR "smart material*" OR "responsive material*" OR "stimuli-responsive material*" OR "stimulus-responsive material*" OR "smart polymer*" OR hydrogel* OR cryogel* OR scaffold* OR bioink* OR nanomaterial* OR nanoparticle* OR nanostructure* OR nanocarrier* OR nanoplatform* OR nanoformulation* OR nanozyme* OR nanogel* OR nanocomposite* OR nanocluster* OR nanofiber* OR nanocage* OR nanovesicle* OR nanosphere* OR "carbon dot*" OR dendrimer* OR liposome* OR micelle* OR microgel* OR microsphere* OR microparticle* OR microcarrier* OR microneedle* OR "drug delivery system*" OR "drug carrier*" OR "delivery platform*" OR "delivery vehicle*" OR "controlled release" OR "sustained release" OR bioprint* OR "3D print*" OR "three-dimensional print*" OR "additive manufactur*" OR "implant surface*" OR "implant coating*" OR "engineered extracellular vesicle*" OR "engineered exosome*" OR "exosome-loaded" OR "exosome-laden" OR "extracellular vesicle-loaded" OR "artificial cell*" OR "DNA tetrahedron*" OR microtopograph* OR "surface stiffness" OR bioceramic* OR "acellular membrane*" OR "decellularized matrix*" OR "metal-organic framework*" OR "wound dressing*" OR "tissue patch*" OR "short fiber*") OR AB=(biomaterial* OR "biocompatible material*" OR "smart material*" OR "responsive material*" OR "stimuli-responsive material*" OR "stimulus-responsive material*" OR "smart polymer*" OR hydrogel* OR cryogel* OR scaffold* OR bioink* OR nanomaterial* OR nanoparticle* OR nanostructure* OR nanocarrier* OR nanoplatform* OR nanoformulation* OR nanozyme* OR nanogel* OR nanocomposite* OR nanocluster* OR nanofiber* OR nanocage* OR nanovesicle* OR nanosphere* OR "carbon dot*" OR dendrimer* OR liposome* OR micelle* OR microgel* OR microsphere* OR microparticle* OR microcarrier* OR microneedle* OR "drug delivery system*" OR "drug carrier*" OR "delivery platform*" OR "delivery vehicle*" OR "controlled release" OR "sustained release" OR bioprint* OR "3D print*" OR "three-dimensional print*" OR "additive manufactur*" OR "implant surface*" OR "implant coating*" OR "engineered extracellular vesicle*" OR "engineered exosome*" OR "exosome-loaded" OR "exosome-laden" OR "extracellular vesicle-loaded" OR "artificial cell*" OR "DNA tetrahedron*" OR microtopograph* OR "surface stiffness" OR bioceramic* OR "acellular membrane*" OR "decellularized matrix*" OR "metal-organic framework*" OR "wound dressing*" OR "tissue patch*" OR "short fiber*") OR AK=(biomaterial* OR "biocompatible material*" OR "smart material*" OR "responsive material*" OR "stimuli-responsive material*" OR "stimulus-responsive material*" OR "smart polymer*" OR hydrogel* OR cryogel* OR scaffold* OR bioink* OR nanomaterial* OR nanoparticle* OR nanostructure* OR nanocarrier* OR nanoplatform* OR nanoformulation* OR nanozyme* OR nanogel* OR nanocomposite* OR nanocluster* OR nanofiber* OR nanocage* OR nanovesicle* OR nanosphere* OR "carbon dot*" OR dendrimer* OR liposome* OR micelle* OR microgel* OR microsphere* OR microparticle* OR microcarrier* OR microneedle* OR "drug delivery system*" OR "drug carrier*" OR "delivery platform*" OR "delivery vehicle*" OR "controlled release" OR "sustained release" OR bioprint* OR "3D print*" OR "three-dimensional print*" OR "additive manufactur*" OR "implant surface*" OR "implant coating*" OR "engineered extracellular vesicle*" OR "engineered exosome*" OR "exosome-loaded" OR "exosome-laden" OR "extracellular vesicle-loaded" OR "artificial cell*" OR "DNA tetrahedron*" OR microtopograph* OR "surface stiffness" OR bioceramic* OR "acellular membrane*" OR "decellularized matrix*" OR "metal-organic framework*" OR "wound dressing*" OR "tissue patch*" OR "short fiber*"))  #3 (TI=("tissue engineer*" OR regenerat* OR "regenerative medicine" OR "tissue repair" OR "bone repair" OR "cartilage repair" OR "wound repair" OR "disc repair" OR "tendon repair" OR "nerve repair" OR "muscle repair" OR "skin repair" OR "organ repair" OR "wound heal*" OR "fracture heal*" OR rejuvenat* OR "functional recovery" OR "functional restoration" OR "tissue reconstruction" OR osteogen* OR chondrogen* OR angiogen* OR neurogen* OR stemness OR "stem cell expansion") OR AB=("tissue engineer*" OR regenerat* OR "regenerative medicine" OR "tissue repair" OR "bone repair" OR "cartilage repair" OR "wound repair" OR "disc repair" OR "tendon repair" OR "nerve repair" OR "muscle repair" OR "skin repair" OR "organ repair" OR "wound heal*" OR "fracture heal*" OR rejuvenat* OR "functional recovery" OR "functional restoration" OR "tissue reconstruction" OR osteogen* OR chondrogen* OR angiogen* OR neurogen* OR stemness OR "stem cell expansion") OR AK=("tissue engineer*" OR regenerat* OR "regenerative medicine" OR "tissue repair" OR "bone repair" OR "cartilage repair" OR "wound repair" OR "disc repair" OR "tendon repair" OR "nerve repair" OR "muscle repair" OR "skin repair" OR "organ repair" OR "wound heal*" OR "fracture heal*" OR rejuvenat* OR "functional recovery" OR "functional restoration" OR "tissue reconstruction" OR osteogen* OR chondrogen* OR angiogen* OR neurogen* OR stemness OR "stem cell expansion"))  #4 #1 AND #2 AND #3 | 21 July 2026 | 685 |
| Scopus | Title, abstract, and keywords (TITLE-ABS-KEY) | TITLE-ABS-KEY( (senescen* OR senolytic* OR senomorphic* OR senotherap* OR "senescence-associated secretory phenotype") AND (biomaterial* OR "biocompatible material*" OR "smart material*" OR "responsive material*" OR "stimuli-responsive material*" OR "stimulus-responsive material*" OR "smart polymer*" OR hydrogel* OR cryogel* OR scaffold* OR bioink* OR nanomaterial* OR nanoparticle* OR nanostructure* OR nanocarrier* OR nanoplatform* OR nanoformulation* OR nanozyme* OR nanogel* OR nanocomposite* OR nanocluster* OR nanofiber* OR nanocage* OR nanovesicle* OR nanosphere* OR "carbon dot*" OR dendrimer* OR liposome* OR micelle* OR microgel* OR microsphere* OR microparticle* OR microcarrier* OR microneedle* OR "drug delivery system*" OR "drug carrier*" OR "delivery platform*" OR "delivery vehicle*" OR "controlled release" OR "sustained release" OR bioprint* OR "3D print*" OR "three-dimensional print*" OR "additive manufactur*" OR "implant surface*" OR "implant coating*" OR "engineered extracellular vesicle*" OR "engineered exosome*" OR "exosome-loaded" OR "exosome-laden" OR "extracellular vesicle-loaded" OR "artificial cell*" OR "DNA tetrahedron*" OR microtopograph* OR "surface stiffness" OR bioceramic* OR "acellular membrane*" OR "decellularized matrix*" OR "metal-organic framework*" OR "wound dressing*" OR "tissue patch*" OR "short fiber*") AND ("tissue engineer*" OR regenerat* OR "regenerative medicine" OR "tissue repair" OR "bone repair" OR "cartilage repair" OR "wound repair" OR "disc repair" OR "tendon repair" OR "nerve repair" OR "muscle repair" OR "skin repair" OR "organ repair" OR "wound heal*" OR "fracture heal*" OR rejuvenat* OR "functional recovery" OR "functional restoration" OR "tissue reconstruction" OR osteogen* OR chondrogen* OR angiogen* OR neurogen* OR stemness OR "stem cell expansion") ) | 21 July 2026 | 1,023 |
| PubMed/MEDLINE | MeSH terms and title/abstract fields | ( "Cellular Senescence"[Mesh] OR "Senescence-Associated Secretory Phenotype"[Mesh] OR senescen*[tiab] OR senolytic*[tiab] OR senomorphic*[tiab] OR senotherap*[tiab] OR "senescence-associated secretory phenotype"[tiab] ) AND ( "Biocompatible Materials"[Mesh] OR "Stimuli Responsive Polymers"[Mesh] OR "Tissue Scaffolds"[Mesh] OR "Hydrogels"[Mesh] OR "Drug Delivery Systems"[Mesh] OR "Drug Carriers"[Mesh] OR "Nanoparticles"[Mesh] OR "Microspheres"[Mesh] OR "Printing, Three-Dimensional"[Mesh] OR "Bioprinting"[Mesh] OR biomaterial*[tiab] OR "biocompatible material*"[tiab] OR "smart material*"[tiab] OR "responsive material*"[tiab] OR "stimuli-responsive material*"[tiab] OR "stimulus-responsive material*"[tiab] OR "smart polymer*"[tiab] OR hydrogel*[tiab] OR cryogel*[tiab] OR scaffold*[tiab] OR bioink*[tiab] OR nanomaterial*[tiab] OR nanoparticle*[tiab] OR nanostructure*[tiab] OR nanocarrier*[tiab] OR nanoplatform*[tiab] OR nanoformulation*[tiab] OR nanozyme*[tiab] OR nanogel*[tiab] OR nanocomposite*[tiab] OR nanocluster*[tiab] OR nanofiber*[tiab] OR nanocage*[tiab] OR nanovesicle*[tiab] OR nanosphere*[tiab] OR "carbon dot*"[tiab] OR dendrimer*[tiab] OR liposome*[tiab] OR micelle*[tiab] OR microgel*[tiab] OR microsphere*[tiab] OR microparticle*[tiab] OR microcarrier*[tiab] OR microneedle*[tiab] OR "drug delivery system*"[tiab] OR "drug carrier*"[tiab] OR "delivery platform*"[tiab] OR "delivery vehicle*"[tiab] OR "controlled release"[tiab] OR "sustained release"[tiab] OR bioprint*[tiab] OR "3D print*"[tiab] OR "three-dimensional print*"[tiab] OR "additive manufactur*"[tiab] OR "implant surface*"[tiab] OR "implant coating*"[tiab] OR "engineered extracellular vesicle*"[tiab] OR "engineered exosome*"[tiab] OR "exosome-loaded"[tiab] OR "exosome-laden"[tiab] OR "extracellular vesicle-loaded"[tiab] OR "artificial cell*"[tiab] OR "DNA tetrahedron*"[tiab] OR microtopograph*[tiab] OR "surface stiffness"[tiab] OR bioceramic*[tiab] OR "acellular membrane*"[tiab] OR "decellularized matrix*"[tiab] OR "metal-organic framework*"[tiab] OR "wound dressing*"[tiab] OR "tissue patch*"[tiab] OR "short fiber*"[tiab] ) AND ( "Tissue Engineering"[Mesh] OR "Regenerative Medicine"[Mesh] OR "Guided Tissue Regeneration"[Mesh] OR "Wound Healing"[Mesh] OR "tissue engineer*"[tiab] OR regenerat*[tiab] OR "regenerative medicine"[tiab] OR "tissue repair"[tiab] OR "bone repair"[tiab] OR "cartilage repair"[tiab] OR "wound repair"[tiab] OR "disc repair"[tiab] OR "tendon repair"[tiab] OR "nerve repair"[tiab] OR "muscle repair"[tiab] OR "skin repair"[tiab] OR "organ repair"[tiab] OR "wound heal*"[tiab] OR "fracture heal*"[tiab] OR rejuvenat*[tiab] OR "functional recovery"[tiab] OR "functional restoration"[tiab] OR "tissue reconstruction"[tiab] OR osteogen*[tiab] OR chondrogen*[tiab] OR angiogen*[tiab] OR neurogen*[tiab] OR stemness[tiab] OR "stem cell expansion"[tiab] ) | 21 July 2026 | 548 |

Note: Search strategies and counts were transcribed from the original project search record; searches were run on 21 July 2026.

**Table S2. Full-text studies included in the core SenescenceBioKG**

| **Record ID** | **First author** | **Year** | **Article title** | **Journal** | **DOI** | **MaterialPlatform** | **Main tissue or disease context** |
| --- | --- | --- | --- | --- | --- | --- | --- |
| FS0001 | Miao X. | 2026 | Construction of biomimetic gradient-structured cartilage organoids and mechanistic study of their application for cartilage rejuvenation | Bioactive Materials | 10.1016/j.bioactmat.2025.12.052 | BGSC-organoid (dCSEV hydrogel-based biomimetic gradient-structured cartilage organoid) | Tissue: Articular cartilage; intervertebral disc; model: DMM-induced knee osteoarthritis; puncture-induced intervertebral disc degeneration |
| FS0005 | Ma X. | 2026 | Ceramic-based biomaterials: Combining regeneration with anti-senescence | Biomaterials | 10.1016/j.biomaterials.2026.124195 | Hardystonite (ZnCS; Ca2ZnSi2O7) | Tissue: Bone; model: OVX osteoporosis; rabbit femoral-condyle defect; OVX mouse systemic osteoporosis |
| FS0007 | Liao B. | 2026 | Denosumab-functionalized nanoparticles target macrophages to mitigate age-related osteoarthritis and osteoporosis | Journal of Materials Chemistry B | 10.1039/d5tb01165e | DS@Lip-FA (folate-modified denosumab-loaded liposomes) | Tissue: Articular cartilage; bone; model: Age-related osteoarthritis and osteoporosis in aged humanized mice |
| FS0009 | Li J. | 2024 | A multilayer hydrogel incorporating urolithin B promotes diabetic wound healing via ROS scavenging and angiogenesis | Chemical Engineering Journal | 10.1016/j.cej.2024.153661 | MRRH@UB multilayer ROS-responsive hydrogel | Tissue: Skin and cutaneous vasculature; model: Full-thickness diabetic skin wound in Sprague–Dawley rats |
| FS0011 | Fukuda N. | 2025 | Accelerated Regeneration of Senescent Bone Injury through Age- and Sex-Independent Macrophage Polarization | ACS Applied Materials and Interfaces | 10.1021/acsami.5c15449 | Phosphatidylserine liposomes (PSLs) used with carbonate-apatite granules | Tissue: Calvarial bone; model: Chronologically aged critical-size calvarial defect |
| FS0013 | Lu M. | 2026 | Bio-inspired artificial antioxidases with synergetic Cu/Zn redox-centers for mitochondrial dynamics regulation and senescent bone regeneration | Nano Today | 10.1016/j.nantod.2026.103043 | PAZ hydrogel containing CZpz-MOF artificial antioxidase | Tissue: Bone; model: DOX-accelerated senile osteoporosis with femoral bone defect in male SD rats |
| FS0016 | Wang D. | 2026 | Intercellular Communication-Driven Mitochondrial Transfer via CXCR4-Engineered Macrophages Reprograms Bone Marrow Metabolism for Osteoporosis Therapy | Advanced Materials | 10.1002/adma.202521645 | CM-MTBM (CXCR4-engineered macrophages loaded with nanozyme-functionalized mitochondria) | Tissue: Bone and bone marrow; model: OVX-induced postmenopausal osteoporosis in female mice |
| FS0018 | He Z. | 2024 | Rejuvenating Aged Bone Repair through Multihierarchy Reactive Oxygen Species-Regulated Hydrogel | Advanced Materials | 10.1002/adma.202306552 | PSeR multihierarchy ROS-regulated hydrogel | Tissue: Bone; model: Naturally aged 20-month-old male mouse distal-femur defect |
| FS0020 | Zhang S. | 2025 | Promoting ROS scavenging and osteogenic differentiation by metal-phenolic network nanoparticle-embedded microenvironment-responsive hydrogel for the repair of senile osteoporotic bone defects | Journal of Materials Chemistry B | 10.1039/d5tb00965k | MPN/BMP@HA hydrogel | Tissue: Bone; model: Aged ovariectomized C57BL/6 mouse femoral-defect model |
| FS0021 | Sun G. | 2026 | A Biologically Lubricating Allicin-Based Nanoplatform for Remodeling the Inflammation-Senescence Axis in the Treatment of Periodontitis | Advanced Materials | 10.1002/adma.202523197 | PPCG allicin-based biolubricating nanoplatform | Tissue: Periodontal soft tissue and alveolar bone; model: Maxillary second-molar ligature-induced periodontitis in C57BL/6 mice |
| FS0022 | Sun Y. | 2024 | Senescence-targeted MicroRNA/Organoid composite hydrogel repair cartilage defect and prevention joint degeneration via improved chondrocyte homeostasis | Bioactive Materials | 10.1016/j.bioactmat.2024.05.036 | MSOH hydrogel | Tissue: Articular cartilage; model: Cartilage/osteochondral defect; Joint degeneration |
| FS0024 | Ding Y. | 2025 | Ceria Centre to Enhance Multi-Active and Photothermal Response Janus Patch for Chronic Wound Senescence Inhibition and Repair | Advanced Healthcare Materials | 10.1002/adhm.202501042 | CeO2@HA-loaded photothermal Janus patch | Tissue: Skin/wound; model: Chronic wound |
| FS0026 | Wang S. | 2025 | Hierarchical ROS-scavenging platform breaks vicious cycle of stem cell senescence, angiogenesis arrest, and immune dysregulation in diabetic wounds | Journal of Controlled Release | 10.1016/j.jconrel.2025.114247 | CaH2-MN hierarchical calcium-hydride microneedle platform | Tissue: Skin/wound; model: Diabetic wound |
| FS0033 | Su W. | 2026 | Targeted delivery of senolytic drugs using macrophage membrane-camouflaged magnetic nanoparticles inhibits endplate sclerosis | Journal of Advanced Research | 10.1016/j.jare.2026.01.054 | MM@MS@ABT macrophage-membrane-coated magnetic nanospheres | Tissue: Liver; model: Age-related Liver regenerative impairment |
| FS0035 | Zhao C. | 2025 |  | Small | 10.1002/smll.202508752 | Mn4 tetranuclear manganese nanocluster | Bone |
| FS0036 | Du Z.-B. | 2025 | A carboxymethyl chitosan/oxidized alginate hydrogel with controlled fullerenol release accelerates diabetic wound healing through cellular senescence reprogramming | Chemical Engineering Journal | 10.1016/j.cej.2025.170469 | CCM@CS-HG hydrogel | Tissue: Skin/wound; model: Diabetic wound |
| FS0038 | Sun X. | 2026 | Iron oxide nanoparticles-driven mitochondrial renewal rejuvenates the aged bone marrow niche | Biomaterials | 10.1016/j.biomaterials.2026.123989 | Iron oxide nanoparticles-driven mitochondrial renewal | Tissue: Bone; model: Age-related Bone regenerative impairment |
| FS0041 | Ma L. | 2026 | From plant to prosthesis: An engineered biomimetic disc repairs senescence-associated intervertebral disc degeneration in goats | Bioactive Materials | 10.1016/j.bioactmat.2026.04.024 | IHP@Yam engineered biomimetic intervertebral disc | Tissue: Intervertebral disc; model: Intervertebral disc degeneration |
| FS0042 | Li Z. | 2025 | MicroSphere 3D Structures Delay Tissue Senescence through Mechanotransduction | ACS Nano | 10.1021/acsnano.4c14874 | MicroSphere and MicroRod anisotropic GelMA microgels |  |
| FS0043 | Yang Z. | 2026 |  | Biomaterials Advances | 10.1016/j.bioadv.2026.214718 | PAA–EDA-grafted porous PEEK scaffold | Tissue: Bone; model: Rabbit femoral-condyle implant model |
| FS0044 | Feng G. | 2025 | Lubricated hydrogel with STING-inhibiting EXOs protect the osteoarthritis by suppressing the senescent microenvironment | Nano Today | 10.1016/j.nantod.2025.102688 | Lubricated hydrogel with STING-inhibiting EXOs | Tissue: Articular cartilage; model: Osteoarthritis |
| FS0048 | Yang F. | 2024 | H2Se-evolving bio-heterojunctions promote cutaneous regeneration in infected wounds by inhibiting excessive cellular senescence | Biomaterials | 10.1016/j.biomaterials.2024.122659 | GO/FeSe2 bio-heterojunction | Tissue: Skin/wound; model: Skin wound/injury |
| FS0049 | Liu Z. | 2026 | Antioxidant Proline Carbon Dot-Composited Poly(L-methionine) Hydrogel Restores Mitophagy to Alleviate Cellular Senescence in Intervertebral Disc Degeneration | Advanced Materials | 10.1002/adma.72951 | Antioxidant Proline Carbon Dot-Composited Poly(L-methionine) Hydrogel | Tissue: Intervertebral disc; model: Intervertebral disc degeneration |
| FS0050 | Qi L. | 2024 | Mesoporous bioactive glass scaffolds for the delivery of bone marrow stem cell-derived osteoinductive extracellular vesicles lncRNA promote senescent bone defect repair by targeting the miR-1843a-5p/Mob3a/YAP axis | Acta Biomaterialia | 10.1016/j.actbio.2024.01.044 | OI-EV-loaded mesoporous bioactive-glass scaffold | Tissue: Bone; model: Bone defect/injury |
| FS0054 | Yu C. | 2025 | Dual-Functional Nanoparticles to Reverse Osteoblast Senescence and Enhance Calcium Supplementation for Alleviating Senile Osteoporosis | Advanced Healthcare Materials | 10.1002/adhm.202501164 | Lipid-coated NMN-loaded amorphous-calcium-carbonate nanoparticles | Tissue: Bone; model: Osteoporosis/osteoporotic defect |
| FS0056 | Yan L. | 2026 | Bioadhesive Hydrogel With Polyphenol-Armored Nanogene Rejuvenates Chondrocyte Senescence for Aged Osteoarthritis Therapy | Advanced Science | 10.1002/advs.202600014 | Polyphenol-armored miR-140-5p nanogene-loaded bioadhesive hydrogel | Tissue: Articular cartilage; model: Osteoarthritis |
| FS0058 | Gao H. | 2025 |  | ACS Biomaterials Science and Engineering | 10.1021/acsbiomaterials.4c02043 | DPMSC-loaded DAT–ALG hydrogel | Tissue: Periodontal tissue; model: Perianal fistula |
| FS0059 | Ye C. | 2026 | Polyphenol mediated zinc-oxygen synergistic hydrogel remodels senescent microenvironment for periodontal tissue regeneration | Nature Communications | 10.1038/s41467-026-70176-x | hZIF8/Mn-hCaO2 composite hydrogel | Tissue: Periodontal tissue; model: Periodontitis |
| FS0062 | Xiong W. | 2024 | In Situ Remodeling of Efferocytosis via Lesion-Localized Microspheres to Reverse Cartilage Senescence | Advanced Science | 10.1002/advs.202400345 | A-Lipo/PAHM lesion-localized hydrogel microspheres | Tissue: Articular cartilage; model: No disease/injury model reported |
| FS0067 | Fan Y. | 2026 | Supramolecular delivery of senolytics enables targeted anti-senescence therapy and accelerated fracture healing | Journal of Nanobiotechnology | 10.1186/s12951-026-04138-2 | Asp10SAC4A supramolecular senolytic nanoplatform | Tissue: Bone; model: Bone fracture |
| FS0071 | Lin W. | 2025 |  | Nature Communications | 10.1038/s41467-025-63376-4 | Remodeling adipocytes’ lipid metabolism with a polycation loaded enzyme-active framework | Tissue: Bone; model: Osteoporosis/osteoporotic defect |
| FS0073 | Zhang W. | 2024 | Self-powered triboelectric-responsive microneedles with controllable release of optogenetically engineered extracellular vesicles for intervertebral disc degeneration repair | Nature Communications | 10.1038/s41467-024-50045-1 | Self-powered triboelectric-responsive EV-releasing microneedles | Tissue: Intervertebral disc; model: Intervertebral disc degeneration |
| FS0077 | Cai X. | 2026 | Nanozyme-Reinforced miR-197-3p Delivery Resets Metabolic and Senescence Pathways to Rejuvenate Osteoarthritic Cartilage | Advanced Science | 10.1002/advs.76364 | miR/PBNP@Gel nanozyme-reinforced miR-197-3p delivery hydrogel | Articular cartilage |
| FS0079 | Qi Y. | 2024 | Enhancement of the function of mesenchymal stem cells by using a GMP-grade three-dimensional hypoxic large-scale production system | Heliyon | 10.1016/j.heliyon.2024.e30968 | GMP-grade TDHLSP glass-microcarrier bioreactor system | No disease/injury model reported |
| FS0080 | Chen J. | 2025 | A multifunctional metformin loaded carboxymethyl chitosan/tannic acid/manganese composite hydrogel with promising capabilities for age-related bone defect repair | Carbohydrate Polymers | 10.1016/j.carbpol.2025.123526 | MCTM metformin-loaded CMCS/tannic-acid/manganese hydrogel | Tissue: Bone; model: Bone defect/injury; Age-related tissue degeneration |
| FS0081 | Cheng Z. | 2025 |  | Langmuir | 10.1021/acs.langmuir.5c04365 | Cu–Fe3O4 nanozyme | Tissue: Skin/wound; model: Age-related tissue degeneration |
| FS0082 | Yin L. | 2026 | 3D-printed PCL/nHA/SA/COL loaded with M2 macrophage-derived exosomes alleviates the senescence of bone marrow mesenchymal stem cells | International Immunopharmacology | 10.1016/j.intimp.2026.116950 | 3D-printed PCL/nHA/SA/COL loaded with M2 macrophage-derived exosomes | Tissue: Bone; model: Bone defect/injury |
| FS0087 | Zhang Y. | 2025 | Recombinant Humanized Collagen: A Promising Treatment for Pelvic Organ Prolapse via Enhanced Fibroblast Function and Angiogenesis | International Urogynecology Journal | 10.1007/s00192-025-06117-x | Recombinant humanized type III collagen (rhCol III) | Pelvic organ prolapse |
| FS0090 | Huang J. | 2026 | Removal of aging microenvironment of bone defects can effectively promote bone defect repair | Journal of Translational Medicine | 10.1186/s12967-026-08500-8 | TSH-Q quercetin-loaded temperature-sensitive hydrogel | Tissue: Bone; model: Bone defect/injury |
| FS0093 | Chen Y. | 2025 | Rescuing inflammatory microenvironment induced senescence via MgO immune mediated asymmetric short fibers for accelerating diabetic wound repair | Materials Today Bio | 10.1016/j.mtbio.2025.102079 | GP@MgO asymmetric short-fiber aerogel | Tissue: Skin/wound; model: Diabetic wound |
| FS0097 | Zhang W. | 2026 | Development-based In Vivo Bioreactor Strategy for Challenging Senescent Bone Reconstruction | Advanced Science | 10.1002/advs.202522408 | BMP-2-loaded in vivo bioreactor scaffold system | Tissue: Bone; model: Bone defect/injury |
| FS0098 | Wang Y. | 2026 |  | Journal of Controlled Release | 10.1016/j.jconrel.2025.114500 | SP@V–C–gel engineered-microalgae DNA hydrogel | Tissue: Intervertebral disc; model: Intervertebral disc degeneration |
| FS0102 |  | 2024 |  | Advanced Materials | 10.1002/adma.202310476 | Porcine small-intestinal-submucosa extracellular-matrix scaffold | Tissue: Skin/wound; model: Skin wound/injury |
| FS0105 | Mao J. | 2026 | Immunoregulatory Nanofiber Patches with Locally Activated Delivery of ECM-Producing Drugs Improve Repair in an Intervertebral Disc Herniation Model | Advanced Fiber Materials | 10.1007/s42765-025-00672-0 | Locally activated TGF-β3/MgSi immunoregulatory nanofiber patch | Tissue: Intervertebral disc; Liver; model: Intervertebral disc herniation |
| FS0107 | Xiong H. | 2024 | Melatonin-loaded bioactive microspheres accelerate aged bone regeneration by formation of tunneling nanotubes to enhance mitochondrial transfer | Materials Today Bio | 10.1016/j.mtbio.2024.101175 | MTBG melatonin-loaded bioactive-glass microspheres | Tissue: Bone; model: Age-related Bone regenerative impairment |
| FS0109 | Pang L. | 2024 | In-situ hydrogen-generating injectable short fibers for osteoarthritis treatment by alleviating oxidative stress | Acta Biomaterialia | 10.1016/j.actbio.2024.09.008 | Hydrogen-generating injectable short-fiber hydrogel | Tissue: Articular cartilage; model: Osteoarthritis |
| FS0111 | Xi L. | 2025 | Retinoic Acid-Loaded Cartilage Organoids Attenuate Chondrocyte Senescence in Osteoarthritis | Journal of Inflammation Research | 10.2147/jir.s545622 | Retinoic-acid-loaded cartilage organoids | Tissue: Articular cartilage; model: Osteoarthritis |
| FS0112 | Wang W. | 2026 | A Pseudo-Mytilus Edulis Foot Protein-Based Hydrogel Adhesive with Osteo-Vascular-Immune Coupling Effects for Osteoporotic Bone-Implant Integration | Advanced Materials | 10.1002/adma.202511840 | YDC-Gel-Zn pseudo-mussel-foot-protein hydrogel adhesive | Tissue: Bone; model: Osteoporosis/osteoporotic defect |
| FS0113 | Xie F. | 2026 | A sustained NAD+supplementation-biosynthesis nanoplatform for metabolic restoration in aged bone regeneration | Bioactive Materials | 10.1016/j.bioactmat.2026.05.016 | N/S@M@P NAD+/SBI nanocomposite hydrogel | Tissue: Bone; model: Age-related Bone regenerative impairment |
| FS0114 | He S.-H. | 2025 | Kartogenin-Loaded Aminated Hollow Mesoporous Prussian Blue Nanozyme-Reinforced Hydrogel Remodels the Senescent Microenvironment via Reactive Oxygen Species Scavenging and Stimulator of Interferon Genes Inhibition to Promote Cartilage Regeneration | Small Structures | 10.1002/sstr.202400592 | NH@K nanozyme-reinforced hydrogel | Articular cartilage |
| FS0117 | Kong L. | 2026 |  | Advanced Science | 10.1002/advs.202524366 | Senomorphic Small Extracellular Vesicles Delivered by a Tissue-Adhesive α-Lipoic-Acid Hydrogel | Tissue: Bone; model: Tendon/ligament injury |
| FS0122 | Wang R. | 2026 | Glycyrrhizic acid-containing Gastrodia elata polysaccharide/chitosan hydrogel promotes macrophage M2 polarization and accelerates aging skin wound healing | Biomaterials Advances | 10.1016/j.bioadv.2025.214571 | GM-Gel glycyrrhizic-acid-microsphere-loaded GEP/chitosan hydrogel | Tissue: Skin/wound; model: Age-related Skin/wound regenerative impairment |
| FS0124 | Li W. | 2024 | Curcumin liposomes alleviate senescence of bone marrow mesenchymal stem cells by activating mitophagy | Scientific Reports | 10.1038/s41598-024-82614-1 | Curcumin-loaded liposomes | Bone |
| FS0125 | Sun Y. | 2024 | Nanotube topography rejuvenates the senescence of mesenchymal stem cells by activating YAP signalling | Journal of Materials Chemistry B | 10.1039/d3tb02828c | Nanotube-topography substrate | Tissue: Periodontal tissue; model: Tendon/ligament injury |
| FS0129 | Zhang W. | 2026 |  | Biomedicine and Pharmacotherapy | 10.1016/j.biopha.2025.118940 | Hypoxic-exosome-loaded PLGA microspheres | Tissue: Intervertebral disc; model: Intervertebral disc degeneration |
| FS0130 | Wang P. | 2024 | Attenuation of osteoarthritis progression via locoregional delivery of Klotho-expressing plasmid DNA and Tanshinon IIA through a stem cell-homing hydrogel | Journal of Nanobiotechnology | 10.1186/s12951-024-02608-z | Plasmid-DNA/Tanshinone-IIA stem-cell-homing hydrogel | Tissue: Articular cartilage; model: Osteoarthritis |
| FS0133 | Peng C. | 2026 | Engineering a quercetin-greigite nanozyme-integrated UV-crosslinked hydrogel with antioxidant and anti-inflammatory properties for treating oxidative stress-driven intervertebral disc degeneration | Chemical Engineering Journal | 10.1016/j.cej.2026.175804 | OGPQ/G-NZ quercetin–greigite nanozyme hydrogel | Tissue: Intervertebral disc; model: Intervertebral disc degeneration |
| FS0134 | Xiao L. | 2026 | Electrospun Scaffold-Enabled Bioreactor for Scalable Manufacturing of Mechanically Programmed Macrophage-Derived Extracellular Vesicles Targeting Aged Bone Defects | Advanced Fiber Materials | 10.1007/s42765-026-00733-y | Electrospun scaffold–bioreactor system | Tissue: Bone; model: Bone defect/injury |
| FS0135 | Zhang X. | 2026 | An electrostatic co-assembled extracellular vesicles infused hydrogel for bone regeneration in aged defects | Biomaterials | 10.1016/j.biomaterials.2026.124254 | Electrostatically co-assembled extracellular-vesicle-infused hydrogel | Tissue: Bone; model: Bone defect/injury |
| FS0136 | Chen J. | 2026 | Injectable CRISPRa-microspheres for targeted A20 activation rescue age-related osteogenic impairment via senescence mitigation | Biomaterials | 10.1016/j.biomaterials.2025.123830 | A20-CRISPRa injectable microspheres | Tissue: Bone; model: Age-related tissue degeneration |
| FS0142 | Gan X. | 2025 | Integrating superlubricative nanomaterials with precision drug delivery for advanced osteoarthritis therapy | Materials Today Bio | 10.1016/j.mtbio.2025.102359 | HPQ@K superlubricative drug-delivery nanoparticles | Tissue: Articular cartilage; model: Osteoarthritis |
| FS0147 | Wu W. | 2026 | Ultrasound-responsive piezoelectric hydrogel attenuates oxidative stress-induced nucleus pulposus senescence via AMPK-FOXO1a-mediated mitophagy for intervertebral disc regeneration | Biomaterials | 10.1016/j.biomaterials.2025.123828 | TT@P-Gel hydrogel | Intervertebral disc |
| FS0148 | Gao W. | 2025 |  | Journal of Colloid and Interface Science | 10.1016/j.jcis.2025.137612 | Se/PRP-OGel hydrogel | Tissue: Articular cartilage; model: Osteoarthritis |
| FS0150 | Wang X. | 2026 |  | Bioactive Materials | 10.1016/j.bioactmat.2026.05.024 | BAI1-PSTB nanocomposite hydrogel | Tissue: Skin/wound; model: Diabetic wound |
| FS0151 | Jiang J. | 2026 | A senescent metabolism-modulating hierarchical scaffold restores NAD+ homeostasis and redox balance for aged bone repair | Bioactive Materials | 10.1016/j.bioactmat.2026.06.036 | senescent metabolism-modulating hierarchical scaffold | Tissue: Bone; model: Age-related Bone regenerative impairment |
| FS0153 | Yuan F. | 2026 | Neonatal muscle-derived extracellular vesicles containing miR-542-3p rejuvenate aged skeletal muscle via a functional microneedle patch | Bioactive Materials | 10.1016/j.bioactmat.2026.06.011 | miR-542-3p neonatal-muscle-EV-loaded microneedle patch | Tissue: Skeletal muscle; model: Age-related Skeletal muscle regenerative impairment |
| FS0157 | Zheng J. | 2026 | Epigenetic reprogramming periosteum promotes aging critical segmental bone defect repair via methylation remodeling | Bioactive Materials | 10.1016/j.bioactmat.2026.03.036 | BFV-loaded engineered periosteum on a 3D-printed scaffold | Tissue: Bone; model: Bone defect/injury |
| FS0164 | Zhang F. | 2025 | In Situ Engineering Mesenchymal Stem Cells for Osteogenic Differentiation to Reverse Inflammaging and Restore Periodontal Bone Homeostasis | Advanced Functional Materials | 10.1002/adfm.202514199 | CTB nanoparticle-engineered MSC construct | Tissue: Periodontal tissue; model: Age-related Periodontal tissue regenerative impairment |
| FS0166 | Li S. | 2025 | On-Demand Collaborative Delivery for Remodeling the Senescent Microenvironment in Intervertebral Disc Degeneration | ACS Nano | 10.1021/acsnano.5c11287 | IL37OI@MS responsive hydrogel microspheres | Tissue: Intervertebral disc; Liver; model: Intervertebral disc degeneration |
| FS0167 | Li Y. | 2024 | Tetrahedral framework nucleic acids promote the proliferation and differentiation potential of diabetic bone marrow mesenchymal stem cell | Chinese Chemical Letters | 10.1016/j.cclet.2023.109414 | Tetrahedral framework nucleic acids | Bone |
| FS0168 | Lu X. | 2025 | Exosome-loaded methacrylated silk fibroin hydrogel delays intervertebral disc degeneration by DKK2-mediated mitochondrial unfolded protein response | Chemical Engineering Journal | 10.1016/j.cej.2025.162191 | SM@ME exosome-loaded methacrylated-silk-fibroin hydrogel | Tissue: Intervertebral disc; model: Intervertebral disc degeneration |
| FS0173 | Li X. | 2026 | Enhanced osteointegration of implants in aged rats via a stem cell pool aging reversion strategy | Biomaterials | 10.1016/j.biomaterials.2025.123604 | Hydrogel-coated micro/nanostructured titanium implant | Tissue: Skin/wound; model: Age-related Skin/wound regenerative impairment |
| FS0178 | Wu W. | 2026 | Microenvironment-educated MSC-EVs loaded injectable smart hydrogel for targeting senescent nucleus pulposus cells and inhibiting ferroptosis against intervertebral disc degeneration | Bioactive Materials | 10.1016/j.bioactmat.2026.02.030 | D-EV-loaded thermosensitive injectable hydrogel | Tissue: Intervertebral disc; model: Intervertebral disc degeneration |
| FS0183 | Lin S. | 2026 | A multifunctional ICAM1-directed MnO?@ZIF8 nanoplatform with Tanshinone IIA synergistically reverses senescence and restores alveolar regeneration in septic lung injury | Journal of Nanobiotechnology | 10.1186/s12951-026-04362-w | ICAM1-directed MnO2@ZIF8/Tanshinone-IIA nanoplatform | Tissue: Lung; model: Septic lung injury |
| FS0184 | Zheng Z. | 2025 | A senolysis-targeting bioenergetic nanoplatform for diabetic wound healing | Advanced Composites and Hybrid Materials | 10.1007/s42114-025-01509-5 | GEA/NGel senolysis-targeting bioenergetic nanoplatform | Tissue: Skin/wound; model: Diabetic wound |
| FS0186 | Zhang X. | 2025 | Cascade Nanozyme-Loaded Sprayable Hydrogels for Fibroblast Rejuvenation and Diabetic Wound Regeneration | ACS Applied Materials and Interfaces | 10.1021/acsami.5c02168 | (ZIF-8@CeO2)@GOx-loaded sprayable GelMA hydrogel | Tissue: Skin/wound; model: Diabetic wound |
| FS0187 | Chen Y. | 2025 | Photothermal Hydrogel with Mn3O4 Nanoparticles Alleviates Intervertebral Disc Degeneration by Scavenging ROS and Regulating Extracellular Matrix Metabolism | Advanced Functional Materials | 10.1002/adfm.202422817 | Mn3O4-nanoparticle photothermal hydrogel | Tissue: Intervertebral disc; model: Intervertebral disc degeneration |
| FS0188 | Zhang P. | 2026 | 3D-Printed StrontiumSilicate Hydrogel Scaffold Promotes Sensory Innervation and Tissue Regeneration Through Modulating Macrophage Senescence | Advanced Functional Materials | 10.1002/adfm.202529224 | GG-5SS strontium-silicate hydrogel scaffold | Tissue: Nervous tissue; model: Skin wound/injury |
| FS0196 | Wang X. | 2025 | Matrix viscoelasticity drives cell cluster formation to counteract cellular senescence | Journal of Materials Chemistry B | 10.1039/d5tb00174a | Polyacrylamide/alginate viscoelastic hydrogel |  |
| FS0199 | Liu P.-L. | 2025 |  | Advanced Healthcare Materials | 10.1002/adhm.202402329 | CK2.1/Lac-Phe bilayer osteochondral scaffold | Tissue: Articular cartilage; model: Cartilage/osteochondral defect |
| FS0203 | Xu F. | 2025 | High-mobility network hydrogel microsphere system to combat chondrocyte senescence for enhanced cartilage repair and regeneration | Materials Today Bio | 10.1016/j.mtbio.2025.102138 | Res@Lipo@HMs hydrogel | Articular cartilage |
| FS0205 | Wang Y. | 2024 | 3D-bioprinted anti-senescence organoid scaffolds repair cartilage defect and prevent joint degeneration via miR-23b/ELOVL5-mediated metabolic rewiring | Chemical Engineering Journal | 10.1016/j.cej.2024.152049 | P16-siRNA-incorporated organoid hydrogel/3D-bioprinted scaffold | Tissue: Articular cartilage; model: Cartilage/osteochondral defect; Joint degeneration |
| FS0207 | Zhang M. | 2026 | Senolytic-loaded asymmetric wound dressing for targeted senescent cell clearance in diabetic wound healing | Materials Today Bio | 10.1016/j.mtbio.2025.102741 | ABT-263-loaded asymmetric composite wound dressing | Tissue: Skin/wound; model: Diabetic wound |
| FS0208 | Dadashpour M. | 2024 | Preparation and in vitro evaluation of cell adhesion and long-term proliferation of stem cells cultured on silibinin co-embedded PLGA/Collagen electrospun composite nanofibers | Experimental Cell Research | 10.1016/j.yexcr.2024.113926 | Silibinin/PLGA/collagen electrospun nanofibers | No disease/injury model reported |
| FS0212 | Gu A. | 2026 | Prussian Blue Nanoparticles Promoting Diabetic Bone Regeneration via Mitochondrial Recovery | BME Frontiers | 10.34133/bmef.0204 | Prussian Blue nanoparticles | Bone |
| FS0213 | Liao B. | 2026 | A cationic thermosensitive polymer scaffold combined with fibroblast growth factor- 4/insulin-like growth factor-1 (FGF-4/IGF-1) promotes liver regeneration in acute liver failure | Colloids and Surfaces B: Biointerfaces | 10.1016/j.colsurfb.2025.115171 | FGF-4/IGF-1-loaded cationic thermosensitive polymer scaffold | Tissue: Liver; model: Acute liver failure |
| FS0219 | Zhu Y. | 2025 | An "inside-out"-guided genetically engineered hydrogel for augmenting aged bone regeneration | Bioactive Materials | 10.1016/j.bioactmat.2025.05.003 | MSN@SIRT3/PEGS genetically engineered hydrogel | Tissue: Bone; model: Age-related Bone regenerative impairment |
| FS0222 | Ma L. | 2026 | Dual-polyphenol ECM-mimetic hydrogel platform attenuates senescence-associated intervertebral disc degeneration through nature-based nanozyme in goats | Chemical Engineering Journal | 10.1016/j.cej.2025.171462 | Zn@MOF-gel dual-polyphenol ECM-mimetic hydrogel | Tissue: Intervertebral disc; model: Intervertebral disc degeneration |
| FS0223 | Yue Z. | 2026 | A photo-responsive, fisetin-functionalized DNA nanocage for programmed therapy to accelerate early-stage wound healing in aged skin | Biomaterials | 10.1016/j.biomaterials.2026.124330 | Fisetin-functionalized photoresponsive DNA nanocage | Tissue: Skin/wound; model: Age-related Skin/wound regenerative impairment |
| FS0226 | Wu Y. | 2025 | Youthful Brain-Derived Extracellular Vesicle-Loaded GelMA Hydrogel Promotes Scarless Wound Healing in Aged Skin by Modulating Senescence and Mitochondrial Function | Research | 10.34133/research.0644 | YBEV-loaded GelMA hydrogel | Tissue: Skin/wound; model: Age-related Skin/wound regenerative impairment |
| FS0227 | Ren B. | 2025 | A multi-effect synergistic photo-therapy PAS/ICG-Cu@ZOL hydrogel dressing with enhancing adaptive immunomodulatory and antibiofilm properties for improved healing of diabetic infected wounds | Chemical Engineering Journal | 10.1016/j.cej.2025.165266 | PAS/ICG-Cu@ZOL phototherapeutic hydrogel dressing | Tissue: Skin/wound; model: Diabetic wound |
| FS0230 | LV D. | 2025 | A biomimetic senotherapy replenishing MAT2A promotes wound regeneration in preclinical models | Nature Communications | 10.1038/s41467-025-65659-2 | Pericyte-membrane-coated saMAT2A lipid nanoparticles | Tissue: Skin/wound; model: Skin wound/injury |
| FS0232 | Wang W. | 2024 | An anti-senescence hydrogel with pH-responsive drug release for mitigating intervertebral disc degeneration and low back pain | Bioactive Materials | 10.1016/j.bioactmat.2024.07.031 | GPQ pH-responsive anti-senescence hydrogel | Tissue: Intervertebral disc; model: Intervertebral disc degeneration |
| FS0234 | Kuboki T. | 2025 | Surface-elastic hydrogels delay senescence via the modulation of redox homeostasis and cytoskeletal tension | Scientific Reports | 10.1038/s41598-025-04779-7 | Surface-elastic StG hydrogels | Tissue: Bone; model: No disease/injury model reported |
| FS0238 | Shen T. | 2026 | Bioactive Nanocomposite-Mediated Macrophage Metabolic Shift Orchestrates Pro-Regenerative Healing in the Diabetic Wound Microenvironment | International Journal of Nanomedicine | 10.2147/ijn.s617254 | PEG-HS/TiO2 nanocomposite | Tissue: Skin/wound; model: Diabetic wound |
| FS0239 | Jang S.-H. | 2025 | FN9-10ELP, an ECM-Mimetic Fusion Protein, Protects Human Mesenchymal Stem Cells from Etoposide-Induced Senescence | International Journal of Molecular Sciences | 10.3390/ijms26189218 | FN9-10ELP extracellular-matrix-mimetic fusion protein | No disease/injury model reported |
| FS0240 | He Z. | 2025 | Selenium-Containing Nano-Micelles Delay the Cellular Senescence of BMSCs Under Oxidative Environment and Maintain Their Regenerative Capacity | Bioengineering | 10.3390/bioengineering12090920 | Selenium-Containing Nano-Micelles | Bone |
| FS0242 | Ding Y. | 2025 |  | Advanced Science | 10.1002/advs.202505009 | EVsTβ4-loaded separable microneedle patch | Tissue: Skin/wound; model: Diabetic wound |
| FS0243 | Hang R. | 2026 | Targeting Inflammaging: A Metformin-Releasing Titanium Implant Reprograms Senescent Macrophages to Orchestrate Angiogenesis and Osteogenesis | Rare Metals | 10.1002/rar2.70447 | Metformin-releasing polydopamine-coated TiO2-nanotube titanium implant | Tissue: Bone; model: Bone defect/injury |
| FS0244 | Tong H. | 2025 | Quercetin prevents the loss of chondrogenic capacity in expansion cultured human auricular chondrocytes by alleviating mitochondrial dysfunction | Regenerative Therapy | 10.1016/j.reth.2025.01.005 | Quercetin-treated human-auricular-chondrocyte/GelMA construct | Tissue: Articular cartilage; model: Cartilage/osteochondral defect |
| FS0247 | Zhou Y. | 2026 | Dual-Target ROS-Driven Spatiotemporal Senolysis for Vascular Repair and Immune Microenvironment Reprogramming in the Treatment of Ocular Fundus Neovascularization | Advanced Science | 10.1002/advs.202523495 | PCC1/PHCF-Gel senolytic hydrogel | Ocular fundus neovascularization |
| FS0248 | Shi G. | 2026 | Versatile smart hydrogels for spatiotemporal drug delivery to orchestrate diabetic bone regeneration | Bioactive Materials | 10.1016/j.bioactmat.2025.09.049 | Smart spatiotemporal drug-delivery hydrogels | Bone |
| FS0250 | Lei H. | 2025 |  | Advanced Materials | 10.1002/adma.202413238 | Functionalized 3D-printed Ti6Al4V cell-climbing-frame scaffold | Tissue: Bone; model: Osteoporosis/osteoporotic defect |
| FS0252 | Wang W. | 2025 | An Immunomodulating Regenerating Hydrogel That Rescues the Oxidative Microenvironment and Reverses Cell Senescence for Osteoporotic Bone Defects | ACS Nano | 10.1021/acsnano.5c06549 | Immunomodulating regenerating hydrogel | Tissue: Bone; model: Osteoporosis/osteoporotic defect; Bone defect/injury |
| FS0261 | Yang L. | 2026 | Oral Delivery of R-spondin1-Loaded Small Extracellular Vesicles Activates WNT Signalling Pathway to Accelerate Intestinal Injury Repair and Reverse Ageing | Journal of Extracellular Vesicles | 10.1002/jev2.70226 | R-spondin1-loaded small extracellular vesicles | Tissue: Liver; model: Liver injury model |
| FS0262 | Zhu D. | 2026 | In vivo circRNA-engineered macrophages mediate localized MMP9 neutralization to rejuvenate aged bone | Bioactive Materials | 10.1016/j.bioactmat.2026.05.030 | PtdSer-modified circRNA-LNP-engineered macrophages | Tissue: Bone; model: Age-related Bone regenerative impairment |
| FS0270 | Zhu M. | 2025 | A multifunctional parathyroid hormone-related supramolecular peptide-loaded dual network hydrogel for radiation-induced wound repair | Materials Today Bio | 10.1016/j.mtbio.2025.101807 | PTHrP supramolecular-peptide-loaded dual-network hydrogel | Skin/wound |
| FS0272 |  | 2025 | Antioxidant and Anti-Senescence Polyvinyl Alcohol-Gallic Acid Supramolecular Hydrogels for Stem Cell Culture | Advanced Healthcare Materials | 10.1002/adhm.202402882 | PVA–gallic-acid supramolecular hydrogel | Tissue: Skin/wound; model: No disease/injury model reported |
| FS0278 | Zhang J. | 2026 |  | ACS Nano | 10.1021/acsnano.5c15543 | Synovium-targeting senolytic liposomes plus M2-macrophage exosomes | Tissue: Articular cartilage; model: Age-related Articular cartilage regenerative impairment |
| FS0279 | Pang Q. | 2024 | Lesion-localized hydrogels functionalized with engineered extracellular matrix restore cellular homeostasis by enhancing mitochondrial energy metabolism for osteoarthritis therapy | Chemical Engineering Journal | 10.1016/j.cej.2024.155662 | Lesion-localized hydrogels functionalized with engineered extracellular matrix | Tissue: Articular cartilage; model: Osteoarthritis |
| FS0282 | Nilawar S. | 2024 | Protective Role of Nanoceria-Infused Nanofibrous Scaffold toward Bone Tissue Regeneration with Senescent Cells | Biomacromolecules | 10.1021/acs.biomac.4c00184 | Nanoceria-infused PCL nanofibrous scaffold | Bone |
| FS0283 | Wang Y. | 2026 | Orbital hybridization-mediated nanozymes reverse cellular senescence for aged bone regeneration | Biomaterials | 10.1016/j.biomaterials.2026.124352 | Orbital hybridization-mediated nanozymes | Tissue: Bone; model: Age-related Bone regenerative impairment |
| FS0284 | Tian S. | 2024 | Multifunctional Hydrogel Microneedle Patches Modulating Oxi-inflamm-aging for Diabetic Wound Healing | Small | 10.1002/smll.202407340 | CeO2@Tau-loaded GelMA microneedle patch | Tissue: Skin/wound; model: Diabetic wound |
| FS0290 | Chen Y. | 2025 | Automated and Enclosed Three-Dimensional Biofabrication System for Mesenchymal Stem Cell Culture to Enhance Diabetic Wound Healing | Biomaterials Research | 10.34133/bmr.0205 | DASEA Regenbio bioreactor–microcarrier culture system | Tissue: Skin/wound; model: Diabetic wound |
| FS0292 | Gao Y. | 2026 | ROS-responsive Ganoderma lucidum polysaccharide nanocomposite targeting prosenescent oxidative stress niche for structural and functional diabetic tendon regeneration | Journal of Nanobiotechnology | 10.1186/s12951-026-04156-0 | Ganoderma-lucidum-polysaccharide nanocomposite | Tissue: Tendon/ligament; model: Tendon/ligament injury |
| FS0295 | Yue G. | 2026 |  | International Journal of Biological Macromolecules | 10.1016/j.ijbiomac.2026.152514 | Sequential release of Zn2+ and rapamycin-loaded EVs from a microsphere–hydrogel platform | Tissue: Skin/wound; model: Diabetic wound |
| FS0296 | Lao A. | 2026 | Senescence-regulating agents remodel mesenchymal stem cell-schwann cell circuitry for diabetic bone regeneration | Biomaterials | 10.1016/j.biomaterials.2025.123656 | MMZ@Si–P53 hydrogel | Tissue: Bone; model: Bone defect/injury |
| FS0300 | Wang P. | 2026 | Injectable microgels carrying engineered biomimetic nanoparticles for osteoarthritis therapy via dual-targeted senescent chondrocyte clearance and endogenous repair promotion | Bioactive Materials | 10.1016/j.bioactmat.2025.11.038 | Injectable microgels carrying engineered biomimetic nanoparticles | Tissue: Articular cartilage; model: Osteoarthritis |
| FS0303 | Fu E. | 2025 | Delivering LINE1 antisense oligonucleotides via endothelial targeting extracellular vesicles to ameliorate myocardial infarction-induced cardiac senescence | Bioactive Materials | 10.1016/j.bioactmat.2025.07.008 | LINE1-ASO-loaded selectin-binding-peptide-engineered extracellular vesicles | Tissue: Cardiac tissue; model: Myocardial infarction |
| FS0306 | Yun Y.-J. | 2025 | UC MSCs Educated Tenon (METn) Stimulates Tendon Regeneration Through Rejuvenation of the Complex and Tendon-Derived Cells (TDCs) | Stem Cells International | 10.1155/sci/8681205 | MSC-educated tendon (METn) construct | Tissue: Tendon/ligament; model: Tendon/ligament injury |
| FS0307 | Zhang X. | 2026 |  | Chinese Journal of Burns and Wounds | 10.3760/cma.j.cn501225-20250106-00009 | Prussian-Blue-nanoparticle/ADSC composite treatment | Tissue: Skin/wound; model: Diabetic wound |
| FS0308 | Zhao K. | 2026 | Nucleus pulposus functional restoration via OSK protein delivery and inflammatory niche remodeling for intervertebral disc repair | Chemical Engineering Journal | 10.1016/j.cej.2026.177276 | OSK-protein-delivery and inflammatory-niche-remodeling platform | Intervertebral disc; Liver |
| FS0310 | Lou Y. | 2025 | Biomimetic rhCOL17-P334 Conjugate for Enhanced Wound Healing | Advanced Materials | 10.1002/adma.202417980 | rhCOL17-P334 conjugate | Tissue: Skin/wound; model: Skin wound/injury |
| FS0311 | Qi L. | 2024 | Magnesium-containing bioceramics stimulate exosomal miR-196a-5p secretion to promote senescent osteogenesis through targeting Hoxa7/MAPK signaling axis | Bioactive Materials | 10.1016/j.bioactmat.2023.10.024 | Magnesium-containing bioceramics stimulate exosomal miR-196a-5p secretion | Bone |
| FS0312 | Luo H. | 2025 | Nanocomposite hydrogel orchestrating multiple modulation of degenerative microenvironment for potential application in intervertebral disc regeneration | Journal of Materials Science and Technology | 10.1016/j.jmst.2024.12.042 | HG-QNT nanocomposite hydrogel | Tissue: Intervertebral disc; model: No disease/injury model reported |
| FS0313 | Fu L. | 2025 | Breaking the vicious cycle of cellular senescence and ROS via a mitochondrial-targeted hydrogel for aged bone regeneration | Chemical Engineering Journal | 10.1016/j.cej.2024.158540 | TPG@ChSMA mitochondrial-targeted hydrogel | Tissue: Bone; model: Age-related Bone regenerative impairment |
| FS0322 | Xie Y. | 2026 | CAP-CD56+CD271+ BMSCs exos-loaded PVA/SA sustained-release hydrogel attenuates chondrocyte senescence and ameliorates lumbar facet joint osteoarthritis | Bioactive Materials | 10.1016/j.bioactmat.2025.10.027 | CAP-CD56+CD271+ BMSC-exosome-loaded PVA/SA hydrogel | Tissue: Articular cartilage; model: Osteoarthritis |
| FS0323 | Li F. | 2024 | Mesoporous MOFs with ROS scavenging capacity for the alleviation of inflammation through inhibiting stimulator of interferon genes to promote diabetic wound healing | Journal of Nanobiotechnology | 10.1186/s12951-024-02423-6 | ROS-scavenging mesoporous MOF nanoparticles | Tissue: Skin/wound; model: Diabetic wound |
| FS0324 | Wang X. | 2024 | Senolytics ameliorate the failure of bone regeneration through the cell senescence-related inflammatory signalling pathway | Biomedicine and Pharmacotherapy | 10.1016/j.biopha.2024.116606 | Senolytic intervention without an engineered carrier | Tissue: Bone; model: Bone defect/injury |
| FS0326 | He S. | 2025 |  | Journal of Nanobiotechnology | 10.1186/s12951-025-03261-w | AAV-loaded extracellular-vesicle biomimetic gene-delivery system | Tissue: Skin/wound; model: Diabetic wound |
| FS0327 | Zhu W. | 2024 | Photothermal Microneedle Hydrogel Patch for Refractory Soft Tissue Injuries through Thermosensitized Anti-Inflammaging Modulation | Small Structures | 10.1002/sstr.202400014 | Taurine@PB hydrogel | Tissue: Skin/wound; model: Age-related Skin/wound regenerative impairment |
| FS0328 | Yang J. | 2026 | Mitochondria-targeted MET-MA carbon dots attenuate radiation-induced bone injury by preventing senescence via miMOMP | Chemical Engineering Journal | 10.1016/j.cej.2026.177769 | Mitochondria-targeted MET-MA carbon dots | Tissue: Bone; model: Bone defect/injury |
| FS0329 | Li F. | 2025 | Microenvironment-responsive MOF nanozymes armored cryogels promoted wound healing via rapid hemostasis, infection elimination and angiogenesis | Journal of Controlled Release | 10.1016/j.jconrel.2025.113838 | MOF-nanozyme-armored cryogel | Tissue: Skin/wound; model: Skin wound/injury |
| FS0338 | Lin Y. | 2025 | MOTS-c-modified functional self-assembly peptide hydrogels enhance the activity of nucleus pulposus-derived mesenchymal stem cells of intervertebral disc degeneration | Materials Today Bio | 10.1016/j.mtbio.2025.101872 | MOTS-c-modified functional self-assembly peptide hydrogels | Tissue: Intervertebral disc; model: Intervertebral disc degeneration |
| FS0341 | Hong Y. | 2025 | Injectable Glycyrrhizic Acid Hydrogel Microspheres With Synergistic Anti-Senescence and Osteogenic Effects for Osteoporosis Therapy | Journal of Biomedical Materials Research - Part A | 10.1002/jbm.a.38016 | Injectable glycyrrhizic-acid hydrogel microspheres | Tissue: Bone; model: Osteoporosis/osteoporotic defect |
| FS0345 | Zhu T. | 2026 | Bone marrow mesenchymal stem cells-laden salidroside-collagen hybrid scaffold (BMSCs-SCOL) accelerates full-thickness skin wound healing by reducing oxidative stress and upregulating transforming growth factor beta | Journal of Materials Science | 10.1007/s10853-025-11867-6 | BMSC-laden salidroside–collagen hybrid scaffold (BMSCs-SCOL) | Tissue: Skin/wound; model: Skin wound/injury |
| FS0346 | Bai F. | 2026 |  | ACS Applied Materials and Interfaces | 10.1021/acsami.5c20823 | Multifunctional pH-responsive microneedles | Bone |
| FS0348 | Sun X. | 2025 | Three-Dimensional Bioprinted Scaffolds Loaded with Multifunctional Magnesium-Based Metal-Organic Frameworks Improve the Senescence Microenvironment Prompting Aged Bone Defect Repair | ACS Nano | 10.1021/acsnano.5c03023 | Three-Dimensional Bioprinted Scaffolds Loaded with Multifunctional Magnesium-Based Metal-Organic Frameworks | Tissue: Bone; model: Bone defect/injury |
| FS0350 | Wang Y. | 2024 | Senescence-Targeted and NAD+-Dependent SIRT1-Activated Nanoplatform to Counteract Stem Cell Senescence for Promoting Aged Bone Regeneration | Small | 10.1002/smll.202304433 | Senescence-targeted NAD+-dependent SIRT1-activating nanoplatform | Tissue: Bone; model: Age-related Bone regenerative impairment |
| FS0356 | Chen Y. | 2025 | A NADH Oxidase Nanozyme Restores Redox Homeostasis to Ameliorate Multi-Organ Aging and Ischemic Cardiomyopathy | Research | 10.34133/research.0973 | NADH-oxidase nanozyme | Tissue: Cardiac tissue; model: Age-related Cardiac tissue regenerative impairment |
| FS0358 | Si C. | 2026 | Bioinspired Microgel Assembly of Liver-Derived Decellularized Extracellular Matrix Enhances Dentin-Pulp Regeneration via Stemness Awakening | Advanced Healthcare Materials | 10.1002/adhm.202503543 | Bioinspired Microgel Assembly of Liver-Derived Decellularized Extracellular Matrix | Tissue: Periodontal tissue; model: Dentin-pulp defect/injury |
| FS0361 | Li J. | 2025 | Functionalized hydrocaffeic acid-chitosan/EGTA hydrogel rescues mitochondrial dysfunction for immunomodulation and joint repair in rheumatoid arthritis | Materials Today Bio | 10.1016/j.mtbio.2025.102605 | Hydrocaffeic-acid–chitosan/EGTA hydrogel | Tissue: Articular cartilage; model: Cartilage/osteochondral defect |
| FS0362 | Bi J. | 2026 | Inflammation-responsive biomimetic hybrid nanovesicles reverse stem cell senescence by up-regulating SIRT1 to treat periodontitis | Journal of Nanobiotechnology | 10.1186/s12951-026-04047-4 | Inflammation-responsive biomimetic hybrid nanovesicles | Tissue: Bone; model: Periodontitis |
| FS0365 | Nie S. | 2026 | Bone-targeted mitochondrial delivery via magnetic-temperature responsive artificial cells for repairing age-related fractures | Frontiers in Pharmacology | 10.3389/fphar.2025.1725973 | Fmito@ACs hydrogel | Tissue: Bone; model: Age-related tissue degeneration |
| FS0367 | Wang Y. | 2025 | Adhesive and injectable hydrogel microspheres for NRF2-mediated periodontal bone regeneration | International Journal of Oral Science | 10.1038/s41368-024-00340-w | Adhesive injectable NRF2-modulating hydrogel microspheres | Tissue: Bone; model: Bone defect/injury; Periodontitis |
| FS0371 | Feng Y. | 2025 | Ultra-small quercetin-based nanotherapeutics ameliorate acute liver failure by combatting inflammation/cellular senescence cycle | Theranostics | 10.7150/thno.103746 | Ultra-small quercetin nanotherapeutics | Tissue: Liver; model: Acute liver failure |
| FS0374 | Pan Z. | 2025 | Engineered Anti-Senescence Trachea With Post-Transplanted Regenerative Homeostasis | Advanced Science | 10.1002/advs.202507186 | Engineered anti-senescence tracheal graft | Tissue: Articular cartilage; model: Age-related tissue degeneration |
| FS0376 | Wang X. | 2025 | Multi-modal microcarriers reprogram mitochondrial metabolism and activate efferocytosis in macrophages for osteoporotic bone repair | Biomaterials | 10.1016/j.biomaterials.2025.123384 | Mitochondrial-metabolism/efferocytosis-modulating microcarriers | Tissue: Bone; model: Osteoporosis/osteoporotic defect |
| FS0380 | Li Y. | 2025 | Synergistic reversal of inflammation-mediated degeneration in intervertebral discs: Phenylboric acid-grafted hyaluronic acid hydrogel as an anti-oxidative vehicle for Timp-3 delivery and promotion of extracellular matrix synthesis | Acta Biomaterialia | 10.1016/j.actbio.2025.06.011 | Phenylboronic-acid-grafted hyaluronic-acid/TIMP-3 hydrogel | Intervertebral disc; Liver |
| FS0382 | Hu C. | 2026 | Nanozyme-Integrated 3D-Printed Gradient Scaffold Rescues Redox Homeostasis for Enhanced Osteochondral Repair | Small Structures | 10.1002/sstr.202500413 | Nanozyme-Integrated 3D-Printed Gradient Scaffold | Tissue: Articular cartilage; model: Cartilage/osteochondral defect |
| FS0384 | Xu G. | 2025 |  | Biomedical Materials (Bristol) | 10.1088/1748-605x/adae6d | α-Arbutin-crosslinked chitosan/gelatin film | Tissue: Bone; model: Age-related Bone regenerative impairment |
| FS0385 | Hu J. | 2025 | Mechanistic study on the alleviation of oxidative stress-induced senescence in nucleus pulposus cells by CRT and the application of its ER-targeted derivative materials | International Journal of Biological Macromolecules | 10.1016/j.ijbiomac.2025.146282 | ER-targeted CRT derivative material | Intervertebral disc |
| FS0386 | Wang Z. | 2025 | Translating m6A-Glycolysis Discovery into Therapy: GOQD-Based Multifunctional Bioactive Scaffolds Rejuvenates Bone Repair in Senescence | Small | 10.1002/smll.202507315 | GOQD-based multifunctional bioactive scaffold | Bone |
| FS0389 | Dong Z. | 2025 | Inhibition and Rescue of Hyperglycemia-Induced Cellular Senescence by Mitochondrial Transfer from Enucleated Mesenchymal Stem Cell-Derived Microvesicles for Chronic Wound Healing | Advanced Science | 10.1002/advs.202501612 | Enucleated-MSC-derived mitochondria-transfer microvesicles | Tissue: Skin/wound; model: Chronic wound |
| FS0391 | He Z. | 2026 |  | Advanced Science | 10.1002/advs.202521619 | rhBMP-2/rapamycin co-treatment without an engineered carrier | Tissue: Bone; model: Age-related Bone regenerative impairment |
| FS0392 | Wu Y. | 2026 | Breaking Age-Impaired Bone Healing Challenge: Smart Hydrogel-Coated Implant Reprograms Aging Bone Microenvironments via Senolytic and Pro-Angiogenic Dual Therapy | Advanced Healthcare Materials | 10.1002/adhm.202501928 | Senolytic/proangiogenic smart hydrogel-coated implant | Tissue: Bone; model: Age-related Bone regenerative impairment |
| FS0393 | Li Q. | 2025 | Inhibiting Cell Inspection Points Intervention Via Injectable Short Fibers for Reversing Neural Cell Senescence | Advanced Fiber Materials | 10.1007/s42765-025-00513-0 | Injectable anti-senescence short fibers | Tissue: Nervous tissue; model: Spinal cord injury |
| FS0394 | Mei X. | 2026 | Sustained release of quercetin through homogeneous poly (lactic-co-glycolic acid) microspheres to enhance MSCs biofunctions for regenerative therapy | Regenerative Therapy | 10.1016/j.reth.2026.101085 | Quercetin-loaded homogeneous PLGA microspheres | Bone |
| FS0395 | Jiang H. | 2026 | Biomimetic Bamboo-Inspired Bone Repair Scaffold: Reversing Degraded States and Promoting Mesenchymal Stem Cell Differentiation | ACS Applied Bio Materials | 10.1021/acsabm.6c00387 | Bamboo-inspired hierarchical bone-repair scaffold | Tissue: Bone; model: Bone defect/injury |
| FS0397 | Wang Y. | 2025 | Cordycepin-Loaded Dental Pulp Stem Cell-Derived Exosomes Promote Aged Bone Repair by Rejuvenating Senescent Mesenchymal Stem Cells and Endothelial Cells | Advanced Healthcare Materials | 10.1002/adhm.202402909 | Cordycepin-Loaded Dental Pulp Stem Cell-Derived Exosomes | Tissue: Bone; model: Age-related Bone regenerative impairment |
| FS0399 | Wang Z. | 2025 | ROS-responsive nanoplatform reverses periodontal senescence via dual-targeted efferocytosis remodeling and mitochondrial homeostasis restoration | Chemical Engineering Journal | 10.1016/j.cej.2025.170411 | FMAG nanoplatform | Bone |
| FS0400 | Sun X. | 2026 | A multimodal ROS logic-gated therapeutic platform disrupts the vicious cycle of senescence to promote aged bone defect repair | Bioactive Materials | 10.1016/j.bioactmat.2026.02.002 | Multimodal ROS-logic-gated therapeutic platform | Tissue: Bone; model: Bone defect/injury |
| FS0401 | Qi L. | 2025 | Engineered extracellular vesicles with sequential cell recruitment and osteogenic functions to effectively promote senescent bone repair | Journal of Nanobiotechnology | 10.1186/s12951-025-03168-6 | Engineered-extracellular-vesicle-loaded GelMA scaffold | Tissue: Bone; model: Bone defect/injury |
| FS0402 | Feng J. | 2026 | An NIR-responsive PDA@RES core-shell nanoplatform accelerates chronic wound healing by remodeling mitochondrial homeostasis and reversing cellular senescence | Nano Research | 10.26599/nr.2026.94908573 | PDA@RES NIR-responsive core–shell nanoplatform | Tissue: Skin/wound; model: Chronic wound |
| FS0404 | Kong K. | 2025 | Delivery of FGF18 using mRNA-LNP protects the cartilage against degeneration via alleviating chondrocyte senescence | Journal of Nanobiotechnology | 10.1186/s12951-025-03103-9 | synFGF18 mRNA lipid nanoparticles | Articular cartilage |
| FS0406 | Lo W.-Y. | 2025 | Topical Application of miR-200b-3p by Poloxamer 407-Based Hydrogel Accelerates Diabetic Wound Healing | Diabetes, Metabolic Syndrome and Obesity | 10.2147/dmso.s538346 | miR-200b-3p-loaded Poloxamer-407 hydrogel | Tissue: Skin/wound; model: Diabetic wound |
| FS0409 | He Y. | 2025 | Curcumin-loaded milk-derived sEVs fused with platelet membrane attenuate endothelial senescence and promote spinal cord injury recovery in diabetic mice | Materials Today Bio | 10.1016/j.mtbio.2025.102036 | Curcumin-loaded milk-sEV/platelet-membrane hybrid vesicles | Tissue: Nervous tissue; model: Spinal cord injury |
| FS0414 | Li W. | 2025 | Injectable hydrogel microspheres that enhance autophagy can improve cartilage microenvironment and resist chondrocyte senescence | Chinese Journal of Tissue Engineering Research | 10.12307/2025.439 | Autophagy-enhancing injectable hydrogel microspheres | Tissue: Articular cartilage; model: No disease/injury model reported |
| FS0418 | Yang H. | 2025 | Multifunctional hydrogel targeting senescence to accelerate diabetic wound healing through promoting angiogenesis | Journal of Nanobiotechnology | 10.1186/s12951-025-03274-5 | GelMA/PNS/Alg@IGF-1 hydrogel | Tissue: Skin/wound; model: Diabetic wound |
| FS0419 | Fan W. | 2025 | A Study of Skeletal Stem Cell Dynamics and Its Potential Applications in the Design of a Titanium Implant for Senile Osteoporosis | Advanced Science | 10.1002/advs.202506982 | Senile-osteoporosis titanium implant | Tissue: Bone; model: Osteoporosis/osteoporotic defect |
| FS0424 | Lu Z. | 2026 | Voronoi curvature scaffolds rejuvenate senescent stem cells and enhance aged bone regeneration | Chemical Engineering Journal | 10.1016/j.cej.2026.178077 | Voronoi-curvature scaffolds | Tissue: Bone; model: Age-related Bone regenerative impairment |
| FS0428 | Cai S. | 2024 | Self-healing hydrogels loaded with Spatholobi Caulis alleviate disc degeneration by promoting autophagy in nucelus pulposus | Materials Today Bio | 10.1016/j.mtbio.2024.101323 | Self-healing hydrogels loaded with Spatholobi Caulis | Tissue: Articular cartilage; Intervertebral disc; Nervous tissue; model: Intervertebral disc degeneration |
| FS0430 | Li K. | 2025 | Nucleus pulposus cell-mimicking nanoparticles for cell-specific HIF1A editing to modulate SASP-mediated disc inflammation via autophagy activation | Acta Biomaterialia | 10.1016/j.actbio.2025.02.060 | HIF1A@NNP nanoplatform | Tissue: Intervertebral disc; model: Bone fracture |
| FS0433 | Song W. | 2025 | Remodeling the Senescent Microenvironment for Promoting Osteoporotic Tendon-to-Bone Healing via Synergizing Senolytic Quercetin and Aligned Nanowire-Structured Hydrogels | ACS Nano | 10.1021/acsnano.5c01332 | Quercetin-loaded aligned-nanowire hydrogel | Tissue: Bone; model: Osteoporosis/osteoporotic defect |
| FS0436 | Wang Z. | 2024 | Multifunctional CeO2 nanozymes for mitigating high-glucose induced senescence and enhancing bone regeneration in type 2 diabetes mellitus | Chemical Engineering Journal | 10.1016/j.cej.2024.149842 | CeO2 nanozymes | Bone |
| FS0438 | Yu Y. | 2025 | Optimized Lipid Nanoparticle-Mediated mRNA Co-Delivery of SOX5/SOX9 Enables Synergistic Senescence Reversal for Osteoarthritis Therapy | International Journal of Nanomedicine | 10.2147/ijn.s544168 | SOX5/SOX9 mRNA lipid nanoparticles | Tissue: Articular cartilage; model: Osteoarthritis |
| FS0439 | Liu H. | 2025 | Fiber scaffolds augment aged bone regeneration by modulating energy metabolism, immunity and angiogenesis | Journal of Controlled Release | 10.1016/j.jconrel.2025.114393 | TA-PFC/PCK fiber scaffold | Tissue: Bone; model: Age-related Bone regenerative impairment |
| FS0440 | Dong Y. | 2026 | Targeting injury-triggered grancalcin from senescent macrophages enhances aged bone healing | Acta Biomaterialia | 10.1016/j.actbio.2025.12.031 | GCA-NAb-loaded GelMA hydrogel | Tissue: Bone; model: Bone injury model |
| FS0446 | Li B. | 2026 | Biomimetic hydrogel delivers engineered exosomes to attenuate cell senescence by AKG-mediated mitochondrial function restoration for osteoarthritis | Materials and Design | 10.1016/j.matdes.2026.116383 | Biomimetic hydrogel delivers engineered exosomes | Tissue: Articular cartilage; model: Osteoarthritis |
| FS0449 | Zhuang Y. | 2024 | Energy metabolism as therapeutic target for aged wound repair by engineered extracellular vesicle | Science Advances | 10.1126/sciadv.adl0372 | Engineered extracellular vesicles | Tissue: Skin/wound; model: Age-related tissue degeneration |
| FS0450 | Zhang Y. | 2024 | Tailoring surface stiffness to modulate senescent macrophage immunomodulation: Implications for osteo-/angio-genesis in aged bone regeneration | Biomaterials Advances | 10.1016/j.bioadv.2024.214010 | Collagen-coated polyacrylamide hydrogels | Tissue: Bone; model: Age-related Bone regenerative impairment |
| FS0453 | Chung S. | 2025 | Nanoparticles for the Delivery of Pro-regenerative Cardiac Progenitor Secretory Proteins Targeting Cellular Senescence and Vasculogenesis | ACS Applied Bio Materials | 10.1021/acsabm.4c01361 | Pro-regenerative cardiac-progenitor-secretome nanoparticles | Cardiac tissue |
| FS0454 | Gao H. | 2026 | A sono-piezoelectric scaffold prompts disc regeneration by activating Ca2+/CaMKII/Parkin-mediated mitophagy | Biomaterials | 10.1016/j.biomaterials.2026.124316 | GBC@PNA sono-piezoelectric scaffold |  |
| FS0458 | Wang J. | 2024 | Regulating chondrocyte senescence through substrate stiffness modulation | Journal of Applied Polymer Science | 10.1002/app.56032 | Tunable-stiffness PDMS substrates | Tissue: Articular cartilage; model: No disease/injury model reported |
| FS0459 | Pang Q. | 2025 | Cartilage-anchoring ECM-functionalized microgels targeting miMOMP to suppress senescence and restore cartilage in osteoarthritis | Chemical Engineering Journal | 10.1016/j.cej.2025.170093 | MG@IFN-γ-ECM cartilage-anchoring microgels | Tissue: Articular cartilage; model: Osteoarthritis |
| FS0460 | Qi L. | 2024 | DNA Tetrahedron Delivering miR-21-5p Promotes Senescent Bone Defects Repair through Synergistic Regulation of Osteogenesis and Angiogenesis | Advanced Healthcare Materials | 10.1002/adhm.202401275 | TDN-miR-21-5p DNA tetrahedron nanocomplex | Tissue: Bone; model: Bone defect/injury |
| FS0461 | Han Y. | 2024 | Active Magnesium Boride/Alginate Hydrogels Rejuvenate Senescent Cells | ACS Nano | 10.1021/acsnano.4c07833 | Active Magnesium Boride/Alginate Hydrogels | Intervertebral disc |
| FS0462 | Zheng Z. | 2026 | On-demand mild photothermal cascade platform reprogramming mitochondrial immunity for tendon rejuvenation | Bioactive Materials | 10.1016/j.bioactmat.2026.01.004 | LT-NPs nanoplatform | Tissue: Tendon/ligament; model: Tendon/ligament injury |
| FS0465 | Wei Q. | 2024 | MiR-17-5p-engineered sEVs Encapsulated in GelMA Hydrogel Facilitated Diabetic Wound Healing by Targeting PTEN and p21 | Advanced Science | 10.1002/advs.202307761 | miR-17-5p-engineered-sEV/GelMA hydrogel | Tissue: Skin/wound; model: Diabetic wound |
| FS0469 | Chen G. | 2025 | Quaternized chitosan-based injectable self-healing hydrogel for improving wound management in aging populations | Colloids and Surfaces B: Biointerfaces | 10.1016/j.colsurfb.2025.114721 | Quaternized-chitosan injectable self-healing hydrogel | Tissue: Skin/wound; model: Age-related Skin/wound regenerative impairment |
| FS0470 |  | 2025 | Development and Characterization of Magnetic Nanoemulsion-Based Senolytic Peptides for Osteoarthritis Treatment | International Journal of Molecular Sciences | 10.3390/ijms26031292 | NE:TUB1-MNP magnetic nanoemulsion | Tissue: Articular cartilage; model: Osteoarthritis |
| FS0471 | Si Y. | 2026 | Bimetallic MOF-Incorporated Hydrogel Scaffolds for Astaxanthin Delivery: Remodeling Bone Microenvironment and Accelerating Bone Repair | Advanced Functional Materials | 10.1002/adfm.202527217 | Astaxanthin-loaded bimetallic-MOF hydrogel scaffold | Bone |
| FS0472 | Zhang S. | 2025 | HydroWrap for T2DM-Related Fractures: A smart H2S-delivery controller modulating Macrophage senescence | Bioactive Materials | 10.1016/j.bioactmat.2025.05.007 | HydroWrap hydrogen-sulfide-delivery hydrogel | Tissue: Bone; model: Bone fracture |
| FS0478 | Wang P. | 2025 | Cell-free osteoarthritis treatment with dual-engineered chondrocyte-targeted extracellular vesicles derived from mechanical loading primed mesenchymal stem cells | Journal of Tissue Engineering | 10.1177/20417314241312563 | Dual-engineered chondrocyte-targeted extracellular vesicles | Tissue: Articular cartilage; model: Osteoarthritis |
| FS0482 | Lv Z. | 2026 | Engineered Exosomal miR-146a-5p Reprograms BMSC Fate and Restores Mitochondrial Homeostasis in Glucocorticoid-Induced Osteonecrosis of Femoral Head | Advanced Science | 10.1002/advs.75897 | miR-146a-5p-engineered exosomes | Tissue: Bone; model: Osteonecrosis |
| FS0487 | Wang C. | 2026 | 3D-printed titanium scaffolds coated with a multifunctional photothermal-responsive hydrogel promote osteoporotic bone defect repair | Materials Today Bio | 10.1016/j.mtbio.2026.102879 | 3D-printed titanium scaffolds coated with a multifunctional photothermal-responsive hydrogel | Tissue: Bone; model: Osteoporosis/osteoporotic defect; Bone defect/injury |
| FS0490 | Liu Y. | 2026 | Dual-responsive injectable hydrogel loaded with DPSCs accelerates diabetic wound healing by promoting mitochondrial transfer to reduce senescent cells | Chemical Engineering Journal | 10.1016/j.cej.2026.177852 | EGCG@MAB hydrogel | Tissue: Skin/wound; model: Diabetic wound |
| FS0492 | Wu T. | 2025 | Bioelectrically Reprogramming Hydrogels Rejuvenate Vascularized Bone Regeneration in Senescence | Advanced Healthcare Materials | 10.1002/adhm.202403837 | Bioelectrically reprogramming hydrogel | Tissue: Bone; model: Bone defect/injury |
| FS0498 | Yu D. | 2026 | Synergistic Piezoelectricity and Lubrication in an Injectable Hydrogel for Osteochondral Regeneration | Advanced Functional Materials | 10.1002/adfm.75561 | G-P-M piezoelectric lubricating injectable hydrogel | Tissue: Articular cartilage; model: Cartilage/osteochondral defect |
| FS0506 | Karim A. | 2024 | Nanoparticle-delivered quercetin exhibits enhanced efficacy in eliminating iron-overloaded senescent chondrocytes | Nanomedicine | 10.1080/17435889.2024.2393074 | Quercetin-loaded nanoparticles | Articular cartilage |
| FS0507 | Wang N. | 2022 | Calcium alginate/silk fibroin peptide/Bletilla striata polysaccharide blended microspheres loaded with tannic acid for rapid wound healing | International Journal of Biological Macromolecules | 10.1016/j.ijbiomac.2022.09.123 | Tannic-acid-loaded calcium-alginate/silk-fibroin-peptide/Bletilla-polysaccharide microspheres | Tissue: Skin/wound; model: Skin wound/injury |
| FS0513 | Abbasi A. | 2022 | Mesenchymal Stem Cell Behavior on Soft Hydrogels with Aligned Surface Topographies | ACS Applied Bio Materials | 10.1021/acsabm.1c01260 | Aligned-topography soft hydrogel substrates | No disease/injury model reported |
| FS0515 | Liu S. | 2014 | Three-dimensional hydrogel scaffolds facilitate in vitro self-renewal of human skin-derived precursors | Acta Biomaterialia | 10.1016/j.actbio.2014.03.018 | Three-dimensional hydrogel scaffolds | Tissue: Skin/wound; model: No disease/injury model reported |
| FS0516 | Qi L. | 2023 | Compromised osteogenic effect of exosomes internalized by senescent bone marrow stem cells via endocytoses involving clathrin, macropinocytosis and caveolae | Frontiers in Bioengineering and Biotechnology | 10.3389/fbioe.2022.1090914 | Extracellular vesicles without an engineered carrier | Tissue: Bone; model: Bone defect/injury |
| FS0522 | Tsai C.-W. | 2018 |  | Artificial Cells, Nanomedicine and Biotechnology | 10.1080/21691401.2017.1394873 | Chitosan-coated culture surfaces | No disease/injury model reported |
| FS0526 | Ahmadi S. | 2023 | Long-term proliferation and delayed senescence of bone marrow-derived human mesenchymal stem cells on metformin co-embedded HA/Gel electrospun composite nanofibers | Journal of Drug Delivery Science and Technology | 10.1016/j.jddst.2022.104071 | Metformin-loaded HA/gelatin electrospun nanofibers | Tissue: Bone; model: Osteoarthritis |
| FS0534 | Wei X. | 2023 | Enzyme-responsive nanospheres target senescent cells for diabetic wound healing by employing chemodynamic therapy | Acta Biomaterialia | 10.1016/j.actbio.2023.10.015 | F@GP galactose-modified Fe3O4-loaded PLGA nanoparticles | Tissue: Skin/wound; model: Diabetic wound |
| FS0535 | Zar?-D?nceanu C.-M. | 2023 | Magnetic Nanoparticle Coating Decreases the Senescence and Increases the Targeting Potential of Fibroblasts and Adipose-Derived Mesenchymal Stem Cells | ACS Omega | 10.1021/acsomega.3c02449 | Magnetic-nanoparticle-coated fibroblast/ADSC constructs | Tissue: Skin/wound; model: Skin wound/injury |
| FS0536 | Dadashpour M. | 2023 | Sustained in vitro delivery of metformin-loaded mesoporous silica nanoparticles for delayed senescence and stemness preservation of adipose-derived stem cells | Journal of Drug Delivery Science and Technology | 10.1016/j.jddst.2023.104769 | Metformin-loaded mesoporous silica nanoparticles | Tissue: Liver; model: No disease/injury model reported |
| FS0538 | Xu Z. | 2023 | Antisenescence ZIF-8/Resveratrol Nanoformulation with Potential for Enhancement of Bone Fracture Healing in the Elderly | ACS Biomaterials Science and Engineering | 10.1021/acsbiomaterials.3c00090 | ZIF-8/resveratrol nanoformulation | Tissue: Bone; model: Bone fracture |
| FS0539 | Mashayekhi S. | 2020 | Curcumin-loaded mesoporous silica nanoparticles/nanofiber composites for supporting long-term proliferation and stemness preservation of adipose-derived stem cells | International Journal of Pharmaceutics | 10.1016/j.ijpharm.2020.119656 | CUR@MSNs nanoplatform | No disease/injury model reported |
| FS0542 | Su X. | 2020 | A bone matrix-simulating scaffold to alleviate replicative senescence of mesenchymal stem cells during long-term expansion | Journal of Biomedical Materials Research - Part A | 10.1002/jbm.a.36958 | nHA/chitosan/PLGA bone-matrix-simulating scaffold | Bone |
| FS0552 | Park H. | 2022 | p16INK4a-siRNA nanoparticles attenuate cartilage degeneration in osteoarthritis by inhibiting inflammation in fibroblast-like synoviocytes | Biomaterials Science | 10.1039/d1bm01941d | p16INK4a-siRNA nanoparticles | Tissue: Articular cartilage; model: Osteoarthritis |
| FS0553 | Wang C. | 2022 |  | Molecular Therapy Nucleic Acids | 10.1016/j.omtn.2021.12.026 | IKKβ-siRNA-loaded gold nanoclusters | Tissue: Tendon/ligament; model: Age-related Tendon/ligament regenerative impairment |
| FS0554 | Ogle M.E. | 2020 | Hydrogel culture surface stiffness modulates mesenchymal stromal cell secretome and alters senescence | Tissue Engineering - Part A | 10.1089/ten.tea.2020.0030 | Stiffness-tunable hydrogel culture surfaces | No disease/injury model reported |
| FS0559 | Younis L.T. | 2018 | 3D TECA hydrogel reduces cellular senescence and enhances fibroblasts migration in wound healing | Asian Journal of Pharmaceutical Sciences | 10.1016/j.ajps.2017.12.003 | Three-dimensional TECA hydrogel | Tissue: Skin/wound; model: Skin wound/injury |
| FS0560 | Yin H. | 2020 | Three-dimensional self-assembling nanofiber matrix rejuvenates aged/degenerative human tendon stem/progenitor cells | Biomaterials | 10.1016/j.biomaterials.2020.119802 | Three-dimensional self-assembling nanofiber matrix | Tissue: Tendon/ligament; model: Age-related Tendon/ligament regenerative impairment |
| FS0563 | Ramalho L. | 2020 | Fibronectin/thermo-responsive polymer scaffold as a dynamic ex vivo niche for mesenchymal stem cells | Journal of Materials Science: Materials in Medicine | 10.1007/s10856-020-06461-y | Fibronectin/thermoresponsive-polymer scaffold | Bone |
| FS0571 | Chen W.-Y. | 2016 | Self-Assembled Heterojunction Carbon Nanotubes Synergizing with Photoimmobilized IGF-1 Inhibit Cellular Senescence | Advanced Healthcare Materials | 10.1002/adhm.201600359 | Heterojunction carbon nanotubes with photoimmobilized IGF-1 | Bone |
| FS0580 | Zhou W. | 2022 | Constructing Tissue-Engineered Dressing Membranes with Adipose-Derived Stem Cells and Acellular Dermal Matrix for Diabetic Wound Healing: A Comparative Study of Hypoxia-or Normoxia-Culture Modes | Stem Cells International | 10.1155/2022/2976185 | ADSC/acellular-dermal-matrix tissue-engineered membrane | Tissue: Skin/wound; model: Diabetic wound |
| FS0595 | Wang X. | 2023 |  | Stem Cell Research and Therapy | 10.1186/s13287-023-03514-1 | Microcarrier–bioreactor culture system | Tissue: Articular cartilage; model: No disease/injury model reported |
| FS0598 |  | 2021 |  | Journal of Personalized Medicine | 10.3390/jpm11010004 | α-Klotho gene-activated scaffold | Tissue: Skin/wound; model: Diabetic wound |
| FS0602 | Yang R. | 2016 |  | ACS Applied Materials and Interfaces | 10.1021/acsami.6b10377 | PAAm–IGF-1/TNF-α biomaterial | Tissue: Liver; model: No disease/injury model reported |
| FS0624 | Guo Z. | 2023 | Carbon Dots from Lycium barbarum Attenuate Radiation-Induced Bone Injury by Inhibiting Senescence via METTL3/Clip3 in an m6A-Dependent Manner | ACS Applied Materials and Interfaces | 10.1021/acsami.3c01322 | Lycium barbarum-derived carbon dots | Tissue: Bone; model: Bone defect/injury |
| FS0628 | Kornicka K. | 2016 |  | RSC Advances | 10.1039/c6ra00029k | Vitamin-E-doped sol–gel silica coating | Tissue: Articular cartilage; Bone; model: No disease/injury model reported |
| FS0630 | Kim S.H. | 2012 | Modulation of the heterogeneous senescence of human mesenchymal stem cells on chemically-modified surfaces | Colloids and Surfaces B: Biointerfaces | 10.1016/j.colsurfb.2011.09.035 | Chemically modified culture surfaces | Tissue: Articular cartilage; Bone; model: No disease/injury model reported |
| FS0637 | Xu X. | 2020 | Fine-tuning of Rat Mesenchymal Stem Cell Senescence via Microtopography of Polymeric Substrates | MRS Advances | 10.1557/adv.2019.446 | Microtopographic polymer substrates | No disease/injury model reported |
| FS0642 | Lu Z. | 2020 | Baghdadite Ceramics Prevent Senescence in Human Osteoblasts and Promote Bone Regeneration in Aged Rats | ACS Biomaterials Science and Engineering | 10.1021/acsbiomaterials.0c01120 | Baghdadite Ceramics | Tissue: Bone; model: Age-related Bone regenerative impairment |
| FS0644 | Hodge J.G. | 2023 | Novel hydrogel system eliminates subculturing and improves retention of nonsenescent mesenchymal stem cell populations | Regenerative Medicine | 10.2217/rme-2022-0140 | Nonsenescent-MSC-retaining hydrogel system | Tissue: Articular cartilage; model: Cartilage/osteochondral defect |
| FS0649 | Xing X. | 2023 | Bone-targeted delivery of senolytics to eliminate senescent cells increases bone formation in senile osteoporosis | Acta Biomaterialia | 10.1016/j.actbio.2022.11.056 | Bone-targeted senolytic delivery system | Tissue: Bone; model: Osteoporosis/osteoporotic defect |
| FS0651 | Lu J. | 2023 | Rejuvenation of tendon stem/progenitor cells for functional tendon regeneration through platelet-derived exosomes loaded with recombinant Yap1 | Acta Biomaterialia | 10.1016/j.actbio.2023.02.018 | Recombinant-Yap1-loaded platelet-derived exosomes | Tissue: Tendon/ligament; model: Tendon/ligament injury |
| FS0655 | Tsai C.-W. | 2015 | Chitosan treatment delays the induction of senescence in human foreskin fibroblast strains | PLoS ONE | 10.1371/journal.pone.0140747 | Chitosan-coated culture surfaces | Tissue: Skin/wound; model: No disease/injury model reported |
| FS0657 | Xing X. | 2022 | Local Elimination of Senescent Cells Promotes Bone Defect Repair during Aging | ACS Applied Materials and Interfaces | 10.1021/acsami.1c22138 | Quercetin/HA-loaded TG-18 hydrogel | Tissue: Bone; model: Bone defect/injury |
| FS0672 | Jin S. | 2023 | Young Exosome Bio-Nanoparticles Restore Aging-Impaired Tendon Stem/Progenitor Cell Function and Reparative Capacity | Advanced Materials | 10.1002/adma.202211602 | Young-donor extracellular-vesicle nanoparticles | Tissue: Tendon/ligament; model: Age-related Tendon/ligament regenerative impairment |
| FS0675 | Deng W. | 2023 | A Senomorphic-Conjugated Scaffold for Application of Senescent Cells in Regenerative Medicine | Advanced Therapeutics | 10.1002/adtp.202200276 | Senomorphic-conjugated scaffold | Tissue: Bone; model: No disease/injury model reported |
| FS0678 | Kornicka K. | 2018 | Released from ZrO2/SiO2 coating resveratrol inhibits senescence and oxidative stress of human adipose-derived stem cells (ASC): Resveratrol-doped biomaterial rejuvenates ASC | Open Chemistry | 10.1515/chem-2018-0039 | Released from ZrO2/SiO2 coating resveratrol | Tissue: Bone; model: No disease/injury model reported |
| FS0687 | Chen S. | 2023 | Local H2 release remodels senescence microenvironment for improved repair of injured bone | Nature Communications | 10.1038/s41467-023-43618-z | CaSi2 nanoparticle-coated mesoporous bioactive-glass scaffold | Bone |
| FS0696 | Zhu J. | 2022 |  | Science Advances | 10.1126/sciadv.abk0011 | miR-29b-5p-loaded stem-cell-homing hydrogel | Tissue: Articular cartilage; model: Osteoarthritis |
| FS0701 | Wei F. | 2021 | Cerium oxide nanoparticles protect against irradiation-induced cellular damage while augmenting osteogenesis | Materials Science and Engineering C | 10.1016/j.msec.2021.112145 | Cerium oxide nanoparticles | Tissue: Bone; model: No disease/injury model reported |
| FS0704 | Guan M. | 2023 | Exosome-laden injectable self-healing hydrogel based on quaternized chitosan and oxidized starch attenuates disc degeneration by suppressing nucleus pulposus senescence | International Journal of Biological Macromolecules | 10.1016/j.ijbiomac.2023.123479 | QCS-OST@Exos self-healing hydrogel | Tissue: Intervertebral disc; model: Intervertebral disc degeneration |
| FS0705 | Wang S. | 2022 | Energy-Supporting Enzyme-Mimic Nanoscaffold Facilitates Tendon Regeneration Based on a Mitochondrial Protection and Microenvironment Remodeling Strategy | Advanced Science | 10.1002/advs.202202542 | CeNP enzyme-mimic nanofiber-bundle scaffold | Tissue: Tendon/ligament; model: Tendon/ligament injury |
| FS0709 | Ren X. | 2023 | Cerium oxide nanoparticles-carrying human umbilical cord mesenchymal stem cells counteract oxidative damage and facilitate tendon regeneration | Journal of Nanobiotechnology | 10.1186/s12951-023-02125-5 | Cerium-oxide-nanoparticle-carrying hUCMSCs | Tissue: Tendon/ligament; model: Tendon/ligament injury |
| FS0720 | Choi S.M. | 2018 | Enhanced articular cartilage regeneration with SIRT1-activated MSCs using gelatin-based hydrogel | Cell Death and Disease | 10.1038/s41419-018-0914-1 | SIRT1-activated-MSC gelatin hydrogel | Tissue: Articular cartilage; model: Cartilage/osteochondral defect |
| FS0726 | Serati-Nouri H. | 2021 | Sustained delivery efficiency of curcumin through ZSM-5 nanozeolites/electrospun nanofibers for counteracting senescence of human adipose-derived stem cells | Journal of Drug Delivery Science and Technology | 10.1016/j.jddst.2021.102902 | Curcumin/ZSM-5 electrospun nanofibers | Tissue: Liver; model: No disease/injury model reported |
| FS0729 | Acevedo C.A. | 2009 | Senescent cultures of human dermal fibroblasts modified phenotype when immobilized in fibrin polymer | Journal of Biomaterials Science, Polymer Edition | 10.1163/156856208x394418 | Fibrin polymer matrix | Tissue: Skin/wound; model: No disease/injury model reported |
| FS0731 | Arcuri S. | 2023 | 3D ECM-Based Scaffolds Boost Young Cell Secretome-Derived EV Rejuvenating Effects in Senescent Cells | International Journal of Molecular Sciences | 10.3390/ijms24098285 | Young-cell-secretome-EV-loaded three-dimensional ECM scaffold |  |
| FS0738 | Liu H. | 2023 | Self-assembly of gelatin microcarrier-based MSC microtissues for spinal cord injury repair | Chemical Engineering Journal | 10.1016/j.cej.2022.138806 | Gelatin-microcarrier-based MSC microtissues | Tissue: Nervous tissue; model: Spinal cord injury |
| FS0761 | Pennarossa G. | 2023 | Synergistic Effect of miR-200 and Young Extracellular Matrix-based Bio-scaffolds to Reduce Signs of Aging in Senescent Fibroblasts | Stem Cell Reviews and Reports | 10.1007/s12015-022-10438-5 | miR-200/young-ECM bioscaffold | No disease/injury model reported |
| FS0765 | Wang X. | 2023 | Conversion of senescent cartilage into a pro-chondrogenic microenvironment with antibody-functionalized copper sulfate nanoparticles for efficient osteoarthritis therapy | Journal of Nanobiotechnology | 10.1186/s12951-023-02036-5 | Antibody-functionalized copper-sulfate nanoparticles | Tissue: Articular cartilage; model: Osteoarthritis |
| FS0770 | Li K. | 2023 | Targeting ROS-induced osteoblast senescence and RANKL production by Prussian blue nanozyme based gene editing platform to reverse osteoporosis | Nano Today | 10.1016/j.nantod.2023.101839 | Prussian-Blue-nanozyme gene-editing platform | Tissue: Bone; model: Osteoporosis/osteoporotic defect |
| FS0776 | Pourpirali R. | 2021 | Prolonged proliferation and delayed senescence of the adipose-derived stem cells grown on the electrospun composite nanofiber co-encapsulated with TiO2 nanoparticles and metformin-loaded mesoporous silica nanoparticles | International Journal of Pharmaceutics | 10.1016/j.ijpharm.2021.120733 | PCL/gelatin nanofibers containing TiO2 nanoparticles and MET@MSNs |  |
| FS0783 | Li B. | 2020 | Regenerative and protective effects of calcium silicate on senescent fibroblasts induced by high glucose | Wound Repair and Regeneration | 10.1111/wrr.12794 | Calcium-silicate ion-releasing biomaterial | Tissue: Skin/wound; model: Diabetic wound |
| FS0788 | Liu S. | 2023 | Tissue Engineering of JAK Inhibitor-Loaded Hierarchically Biomimetic Nanostructural Scaffold Targeting Cellular Senescence for Aged Bone Defect Repair and Bone Remolding | Advanced Healthcare Materials | 10.1002/adhm.202301798 | Ruxolitinib-loaded hierarchically biomimetic nanostructural scaffold | Tissue: Bone; model: Bone defect/injury |
| FS0795 | Cho K.-H. | 2011 | Enhanced delivery of rapamycin by V156K-apoA-I high-density lipoprotein inhibits cellular proatherogenic effects and senescence and promotes tissue regeneration | Journals of Gerontology - Series A Biological Sciences and Medical Sciences | 10.1093/gerona/glr169 | V156K-rapa-rHDL nanoparticles | Tissue: Liver; model: Skin wound/injury |
| FS0796 | Kim Y.H. | 2021 | Anti-senescence ion-delivering nanocarrier for recovering therapeutic properties of long-term-cultured human adipose-derived stem cells | Journal of Nanobiotechnology | 10.1186/s12951-021-01098-7 | Iron–gold anti-senescence ion nanocarrier (AIN) | Liver |
| FS0797 | Zhou L. | 2018 | SIRT1-dependent anti-senescence effects of cell-deposited matrix on human umbilical cord mesenchymal stem cells | Journal of Tissue Engineering and Regenerative Medicine | 10.1002/term.2422 | Cell-deposited extracellular matrix | Tissue: Articular cartilage; model: No disease/injury model reported |
| FS0809 | Katoh S. | 2021 | Reversal of senescence-associated beta-galactosidase expression during in vitro three-dimensional tissue-engineering of human chondrocytes in a polymer scaffold | Scientific Reports | 10.1038/s41598-021-93607-9 | Human-chondrocyte/polymer-scaffold tissue-engineered construct | Tissue: Articular cartilage; model: Age-related Articular cartilage regenerative impairment |
| FS0810 | Yuan T. | 2023 | All-in-one smart dressing for simultaneous angiogenesis and neural regeneration | Journal of Nanobiotechnology | 10.1186/s12951-023-01787-5 | ASD all-in-one smart hydrogel dressing | Skin/wound |
| FS0816 | Cao H. | 2023 | Cell-Free Osteoarthritis Treatment with Sustained-Release of Chondrocyte-Targeting Exosomes from Umbilical Cord-Derived Mesenchymal Stem Cells to Rejuvenate Aging Chondrocytes | ACS Nano | 10.1021/acsnano.3c01612 | Chondrocyte-targeting exosome sustained-release platform | Tissue: Articular cartilage; model: Osteoarthritis |
| FS0821 | Zhao R. | 2022 | Precise Diabetic Wound Therapy: PLS Nanospheres Eliminate Senescent Cells via DPP4 Targeting and PARP1 Activation | Advanced Science | 10.1002/advs.202104128 | PARP1@PLS-PT100 targeted nanospheres | Tissue: Skin/wound; model: Diabetic wound |
| FS0834 | Chen M. | 2022 | Nanocatalytic Biofunctional MOF Coating on Titanium Implants Promotes Osteoporotic Bone Regeneration through Cooperative Pro-osteoblastogenesis MSC Reprogramming | ACS Nano | 10.1021/acsnano.2c07200 | Nanocatalytic MOF-coated titanium implant | Tissue: Bone; model: Osteoporosis/osteoporotic defect |
| FS0838 | Song L. | 2022 | Development of scaffold-free micro-tissues to accelerate soft and hard tissue regeneration via delaying cellular senescence and regulating inflammation | Applied Materials Today | 10.1016/j.apmt.2022.101370 | Scaffold-free microtissues | Tissue: Skin/wound; model: Skin wound/injury |
| FS0842 | Kornicka K. | 2017 | Polyurethane-polylactide-based material doped with resveratrol decreases senescence and oxidative stress of adipose-derived mesenchymal stromal stem cell (ASCs) | RSC Advances | 10.1039/c7ra02334k | Resveratrol-doped polyurethane–polylactide material | Bone |
| FS0843 | Peng Y. | 2023 | Extracellular Vesicle-Conjugated Functional Matrix Hydrogels Prevent Senescence by Exosomal miR-3594-5p-Targeted HIPK2/p53 Pathway for Disc Regeneration | Small | 10.1002/smll.202206888 | Extracellular-vesicle-conjugated functional-matrix hydrogel |  |
| FS0866 | Honda Y. | 2020 | Augmentation of bone regeneration by depletion of stress-induced senescent cells using catechin and senolytics | International Journal of Molecular Sciences | 10.3390/ijms21124213 | LPS-releasing gelatin sponges with catechin/senolytic treatment | Tissue: Bone; model: Bone defect/injury |
| FS0879 | Tian Y. | 2023 |  | International Journal of Molecular Sciences | 10.3390/ijms24119766 | ZIM1/hydrogel culture system |  |
| FS0882 | Choi D.H. | 2021 | Tonsil-derived mesenchymal stem cells incorporated in reactive oxygen species-releasing hydrogel promote bone formation by increasing the translocation of cell surface GRP78 | Biomaterials | 10.1016/j.biomaterials.2021.121156 | Tonsil-derived mesenchymal stem cells incorporated in reactive oxygen species-releasing hydrogel | Bone |
| FS0885 | Lee H. | 2020 | Derivation of cell-engineered nanovesicles from human induced pluripotent stem cells and their protective effect on the senescence of dermal fibroblasts | International Journal of Molecular Sciences | 10.3390/ijms21010343 | Human-iPSC-derived cell-engineered nanovesicles | Tissue: Skin/wound; model: Skin wound/injury |
| FS0887 | Dhanabalan K.M. | 2020 | Rapamycin-PLGA microparticles prevent senescence, sustain cartilage matrix production under stress and exhibit prolonged retention in mouse joints | Biomaterials Science | 10.1039/d0bm00596g | Rapamycin-PLGA microparticles | Articular cartilage |
| FS0905 | Serati-Nouri H. | 2021 | In vitro expansion of human adipose-derived stem cells with delayed senescence through dual stage release of curcumin from mesoporous silica nanoparticles/electrospun nanofibers | Life Sciences | 10.1016/j.lfs.2021.119947 | Curcumin-loaded mesoporous-silica/PCL-gelatin electrospun nanofiber scaffold | No disease/injury model reported |
| FS0912 | Ashiba K. | 2023 | Senescence recovering by dual drug-encapsulated liposomal nanoparticles for large-scale human cell expansion | Journal of Artificial Organs | 10.1007/s10047-022-01356-x | Dual-drug-encapsulated liposomal nanoparticles | No disease/injury model reported |
| FS0913 | Liu C. | 2023 | A Redox Homeostasis Modulatory Hydrogel with GLRX3+ Extracellular Vesicles Attenuates Disc Degeneration by Suppressing Nucleus Pulposus Cell Senescence | ACS Nano | 10.1021/acsnano.3c01713 | GLRX3+ extracellular-vesicle redox-modulatory hydrogel | Tissue: Intervertebral disc; model: Intervertebral disc degeneration |
| FS0916 | Das A. | 2020 | Honey-incorporated nanofibre reduces replicative senescence of umbilical cordderived mesenchymal stem cells | IET Nanobiotechnology | 10.1049/iet-nbt.2019.0288 | Honey-incorporated electrospun nanofiber membrane | Skin/wound |
| FS0927 | Chang D.F. | 2024 | Telomerase mRNA Enhances Human Skin Engraftment for Wound Healing | Advanced Healthcare Materials | 10.1002/adhm.202302029 | hTERT mRNA lipid nanoparticles | Tissue: Skin/wound; model: Skin wound/injury |
| FS0928 | Xu L. | 2021 | Metformin Hydrochloride Encapsulation by Alginate Strontium Hydrogel for Cartilage Regeneration by Reliving Cellular Senescence | Biomacromolecules | 10.1021/acs.biomac.0c01488 | Alginate–strontium/metformin-hydrochloride hydrogel beads | Articular cartilage |
| FS0932 | Ke S. | 2018 | Molybdenum Disulfide Nanoparticles Resist Oxidative Stress-Mediated Impairment of Autophagic Flux and Mitigate Endothelial Cell Senescence and Angiogenic Dysfunctions | ACS Biomaterials Science and Engineering | 10.1021/acsbiomaterials.7b00714 | Molybdenum disulfide nanoparticles | No disease/injury model reported |
| FS0956 | Mukhopadhyay A. | 2021 | Improved Mesenchymal Stem Cell Proliferation, Differentiation, Epithelial Transition, and Restrained Senescence on Hierarchically Patterned Porous Honey Silk Fibroin Scaffolds | ACS Applied Bio Materials | 10.1021/acsabm.1c00115 | Hierarchically patterned porous honey/silk-fibroin scaffold | Tissue: Articular cartilage; Bone; model: No disease/injury model reported |
| FS0970 | Dong S. | 2023 | Sandblasted/Acid-Etched Titanium Surface Modified with Calcium Phytate Enhances Bone Regeneration in a High-Glucose Microenvironment by Regulating Reactive Oxygen Species and Cell Senescence | ACS Biomaterials Science and Engineering | 10.1021/acsbiomaterials.3c00385 | Sandblasted/Acid-Etched Titanium Surface Modified with Calcium Phytate | Bone |
| FS1020 | Su, ZX Shi, YQ Lu, ZY Li, RL Wang, XL Ning, BB Duan, JL Hao, LS Duan, JH Li, Y Zhu, Y Hao, CN Wang, R | 2018 | Comparative study of nanostructured carriers of calcium phosphate and magnesium phosphate loaded with SRT1720 for the protection of H2O2-induced senescent endothelium | AMERICAN JOURNAL OF TRANSLATIONAL RESEARCH |  | SRT1720-loaded calcium-phosphate/magnesium-phosphate nanocarriers | No disease/injury model reported |
| FS1021 | Rao, VV Vu, MK Ma, H Killaars, AR Anseth, KS | 2019 | Rescuing mesenchymal stem cell regenerative properties on hydrogel substrates post serial expansion | BIOENGINEERING & TRANSLATIONAL MEDICINE | 10.1002/btm2.10104 | Hydrogel substrates for serially expanded MSCs | Tissue: Articular cartilage; model: Cartilage/osteochondral defect |
| FS1040 | Yang, Yusheng | 2026 | Hairyvein agrimony-derived nanovesicles in microneedles eradicate intracellular bacteria and reverse senescence to heal diabetic foot osteomyelitis. | Journal of controlled release : official journal of the Controlled Release Society | 10.1016/j.jconrel.2026.115175 | Hairyvein-agrimony-nanovesicle-loaded microneedles | Tissue: Bone; model: Diabetic foot osteomyelitis |
| FS1052 | Wang, Kelei | 2026 | PCET-Inspired Nanoparticles Enable Paired Proton-Electron Delivery to Restore Mitochondrial Electron Transport and Regenerate Bone During Aging. | Advanced materials (Deerfield Beach, Fla.) | 10.1002/adma.74133 | EMHS HxMoO3-loaded bone-targeting lipid nanoparticles | Tissue: Bone; model: Age-related Bone regenerative impairment |
| FS1054 | Wang, Yifan | 2026 | An ECM-mimetic hydrogel for disc repair: reconstituting hypoxia and alleviating NPC senescence to halt intervertebral disc degeneration. | Journal of nanobiotechnology | 10.1186/s12951-026-04779-3 | HPTC ECM-mimetic injectable hydrogel | Tissue: Intervertebral disc; model: Intervertebral disc degeneration |

Note: Rows follow the frozen Record ID order. Blank cells indicate that a reliable value was not represented in the frozen source fields.

**Table S3. Ontology entity types and canonical node counts in the frozen SenescenceBioKG**

| **Entity type** | **Operational definition** | **Instantiated** | **Canonical node count** | **Example or note** |
| --- | --- | --- | --- | --- |
| Study | A source article identified by Record ID and DOI. | No | 0 | Ontology-defined; no canonical nodes in the frozen graph. |
| MaterialPlatform | A material, formulation, carrier or engineered construct that is the source node of validated edges. | Yes | 281 | Example: (ZIF-8@CeO2)@GOx-loaded sprayable GelMA hydrogel |
| MaterialComposition | A verified constituent of a material platform. | Yes | 180 | Example: ABT-263 |
| FabricationStrategy | A verified material manufacturing or processing operation. | Yes | 44 | Example: 3D printing/bioprinting |
| DesignMode | A material/platform design class used by a validated study system. | Yes | 15 | Example: Biomimetic ECM/cell construct |
| Stimulus | A stimulus directly causing a validated material behavior change. | Yes | 16 | Example: Acidic pH |
| ResponsiveBehavior | A directly observed material response behavior; retained as an ontology type even when not the edge target. | No | 0 | Ontology-defined; no canonical nodes in the frozen graph. |
| TherapeuticCargo | A therapeutic molecule, protein, nucleic acid, organelle, cell product or ion actually delivered/released by a platform. | Yes | 62 | Example: AAV-Sirt3 |
| TargetCell | A cell type directly tested as an intervention target. | Yes | 16 | Example: Cardiomyocytes/cardiac progenitors |
| SenescenceEvidence | A senescence marker or induction evidence class; retained as an ontology type for future extension. | No | 0 | Ontology-defined; no canonical nodes in the frozen graph. |
| Mechanism | A mechanism, pathway or molecular node directly supported by study evidence. | Yes | 22 | Example: AMPK–mTOR–sirtuin signaling |
| DiseaseModel | An experimental disease/injury model; retained as an ontology type for future extension. | No | 0 | Ontology-defined; no canonical nodes in the frozen graph. |
| RegenerativeEndpoint | A directly reported repair or regenerative endpoint. | Yes | 12 | Example: Bone regeneration/osteogenesis |
| ValidationStage | The actual experimental validation level. | Yes | 6 | Example: Cell |

Note: Study, ResponsiveBehavior, SenescenceEvidence, and DiseaseModel were ontology-defined but had no canonical node instances in the frozen graph.

**Table S4. Final relation-level adjudication in SenescenceBioKG**

| **Relation** | **Final adjudicated universe** | **SUPPORTED** | **REJECTED** | **UNCLEAR** | **Support rate (%)** |
| --- | --- | --- | --- | --- | --- |
| ACTIVATES | 0 | 0 | 0 | 0 |  |
| COMPOSED OF | 489 | 213 | 236 | 40 | 43.6 |
| DELIVERS | 222 | 71 | 135 | 16 | 32.0 |
| FABRICATED BY | 337 | 162 | 86 | 89 | 48.1 |
| MODULATES | 271 | 88 | 167 | 16 | 32.5 |
| PROMOTES | 284 | 107 | 169 | 8 | 37.7 |
| RELEASES | 207 | 61 | 130 | 16 | 29.5 |
| RESPONDS TO | 71 | 64 | 7 | 0 | 90.1 |
| SUPPRESSES | 0 | 0 | 0 | 0 |  |
| TARGETS | 496 | 313 | 112 | 71 | 63.1 |
| USES | 335 | 222 | 36 | 77 | 66.3 |
| VALIDATED AT | 691 | 567 | 124 | 0 | 82.1 |

Note: SUPPORTED + REJECTED + UNCLEAR equals the final adjudicated universe in every row. Support rate is not calculated where the universe is zero.

**Table S5. Operational definitions and study coverage of validation categories**

| **ValidationStage** | **Operational definition** | **Included setting** | **Key exclusion** | **Unique studies** |
| --- | --- | --- | --- | --- |
| Cell | Validation performed in cultured cells without a higher-order tissue model. | Two-dimensional or three-dimensional cell experiments in vitro. | Ex vivo tissue, organoid/chip, animal in vivo, or human-participant intervention. | 280 |
| Ex vivo | Validation performed in tissue or organ material maintained outside a living organism. | Explants, tissue slices, or isolated organs assessed ex vivo. | Cell culture alone and implantation in a living animal. | 6 |
| Organoid/chip | Validation performed in an organoid or engineered organ-on-chip system. | Organoids, assembloids, or microphysiological chip models. | Standard cell culture, excised tissue alone, and animal in vivo studies. | 6 |
| Small Animal | The material intervention is evaluated in vivo in a small-animal model. | In vivo mouse, rat, rabbit, or other study-defined small-animal intervention. | Small-animal-derived material without in vivo intervention. | 217 |
| Large Animal | The material intervention is evaluated in vivo in a large-animal model. | In vivo pig, sheep, goat, dog, or other study-defined large-animal intervention. | Large-animal-derived biomaterial without in vivo intervention. | 4 |
| Human-derived | Evidence is generated from cells, tissues, or samples obtained from humans. | Patient- or donor-derived cells, tissues, specimens, or human datasets. | Human-derived evidence is not Clinical validation. | 54 |
| Clinical | The material intervention is administered to human participants with reported outcomes. | Prospective or interventional use in human participants with outcome reporting. | Patient-derived samples, retrospective data, and non-interventional human evidence. | 0 |

Note: Human-derived evidence is distinct from Clinical validation. A large-animal-derived biomaterial does not constitute Large Animal validation without an in vivo intervention.

**Table S6. Full set of explicitly stimulus-responsive material systems**

| **Record ID** | **MaterialPlatform** | **Stimulus** | **RESPONDS TO Evidence ID** | **RELEASES** | **TARGETS** | **MODULATES** | **PROMOTES** | **ValidationStage** |
| --- | --- | --- | --- | --- | --- | --- | --- | --- |
| FS0013 | PAZ hydrogel containing CZpz-MOF artificial antioxidase | Mechanical force | EV000030 | No | No | No | No | Cell; Small Animal |
| FS0036 | CCM@CS-HG hydrogel | Glucose | EV000086 | Yes | Yes | Yes | Yes | Cell; Small Animal |
| FS0036 | CCM@CS-HG hydrogel | ROS | EV000087 | Yes | Yes | Yes | Yes | Cell; Small Animal |
| FS0067 | Asp10SAC4A supramolecular senolytic nanoplatform | Hypoxia | EV000165 | Yes | Yes | No | No | Cell; Small Animal |
| FS0142 | HPQ@K superlubricative drug-delivery nanoparticles | ROS | EV000367 | No | Yes | Yes | Yes | Cell; Human-derived; Small Animal |
| FS0147 | TT@P-Gel hydrogel | Ultrasound | EV000375 | Yes | Yes | Yes | No | Cell; Small Animal |
| FS0173 | Hydrogel-coated micro/nanostructured titanium implant | ROS | EV000440 | No | Yes | Yes | No | Cell; Small Animal |
| FS0178 | D-EV-loaded thermosensitive injectable hydrogel | ROS | EV000446 | No | Yes | Yes | No | Cell; Small Animal |
| FS0183 | ICAM1-directed MnO2@ZIF8/Tanshinone-IIA nanoplatform | ROS | EV000454 | No | Yes | No | No | Cell; Organoid/chip; Small Animal |
| FS0223 | Fisetin-functionalized photoresponsive DNA nanocage | Light | EV000561 | Yes | Yes | No | No | Cell; Human-derived; Small Animal |
| FS0247 | PCC1/PHCF-Gel senolytic hydrogel | ROS | EV000637 | Yes | Yes | No | No | Cell; Small Animal |
| FS0252 | Immunomodulating regenerating hydrogel | ROS | EV000656 | No | Yes | Yes | No | Cell; Small Animal |
| FS0308 | OSK-protein-delivery and inflammatory-niche-remodeling platform | Glutathione | EV000765 | No | Yes | Yes | No | Cell; Small Animal |
| FS0400 | Multimodal ROS-logic-gated therapeutic platform | ROS | EV001036 | Yes | Yes | Yes | Yes | Cell; Small Animal |
| FS0419 | Senile-osteoporosis titanium implant | ROS | EV001076 | No | Yes | No | No | Cell |
| FS0454 | GBC@PNA sono-piezoelectric scaffold | Ultrasound | EV001163 | No | Yes | No | Yes | Cell; Human-derived; Small Animal |
| FS0462 | LT-NPs nanoplatform | ROS | EV001193 | Yes | Yes | No | No | Cell; Small Animal |
| FS0469 | Quaternized-chitosan injectable self-healing hydrogel | Mechanical strain | EV001206 | No | No | No | No | Cell; Small Animal |
| FS0490 | EGCG@MAB hydrogel | ROS | EV001260 | No | Yes | Yes | Yes | Cell; Small Animal |
| FS0534 | F@GP galactose-modified Fe3O4-loaded PLGA nanoparticles | Magnetic field | EV001319 | Yes | Yes | No | No | Cell; Small Animal |
| FS0704 | QCS-OST@Exos self-healing hydrogel | Mechanical force | EV001472 | No | Yes | No | Yes | Cell; Small Animal |
| FS0009 | MRRH@UB multilayer ROS-responsive hydrogel | ROS | EV001715 | No | Yes | No | Yes | Cell; Small Animal |
| FS0020 | MPN/BMP@HA hydrogel | Acidic pH | EV001719 | Yes | No | No | No | Cell; Small Animal |
| FS0038 | Iron oxide nanoparticles-driven mitochondrial renewal | Acidic pH | EV001723 | No | Yes | No | No | Cell; Small Animal |
| FS0056 | Polyphenol-armored miR-140-5p nanogene-loaded bioadhesive hydrogel | Mechanical force | EV001730 | No | Yes | No | No | Cell; Ex vivo; Small Animal |
| FS0073 | Self-powered triboelectric-responsive EV-releasing microneedles | Electrical stimulation | EV001733 | No | Yes | No | No | Cell; Small Animal |
| FS0105 | Locally activated TGF-β3/MgSi immunoregulatory nanofiber patch | Acidic pH | EV001740 | Yes | Yes | No | No | Cell; Small Animal |
| FS0133 | OGPQ/G-NZ quercetin–greigite nanozyme hydrogel | Acidic pH | EV001744 | Yes | Yes | Yes | No | Cell; Small Animal |
| FS0133 | OGPQ/G-NZ quercetin–greigite nanozyme hydrogel | ROS | EV001745 | Yes | Yes | Yes | No | Cell; Small Animal |
| FS0142 | HPQ@K superlubricative drug-delivery nanoparticles | Acidic pH | EV001747 | No | Yes | Yes | Yes | Cell; Human-derived; Small Animal |
| FS0148 | Se/PRP-OGel hydrogel | Acidic pH | EV001749 | No | Yes | Yes | No | Cell; Small Animal |
| FS0166 | IL37OI@MS responsive hydrogel microspheres | Acidic pH | EV001753 | No | Yes | Yes | No | Cell; Small Animal |
| FS0183 | ICAM1-directed MnO2@ZIF8/Tanshinone-IIA nanoplatform | Acidic pH | EV001755 | No | Yes | No | No | Cell; Organoid/chip; Small Animal |
| FS0232 | GPQ pH-responsive anti-senescence hydrogel | Acidic pH | EV001763 | Yes | Yes | No | No | Cell; Small Animal |
| FS0248 | Smart spatiotemporal drug-delivery hydrogels | Acidic pH | EV001767 | No | Yes | Yes | Yes | Cell; Human-derived; Small Animal |
| FS0296 | MMZ@Si–P53 hydrogel | Acidic pH | EV001773 | No | Yes | Yes | Yes | Cell; Small Animal |
| FS0346 | Multifunctional pH-responsive microneedles | Acidic pH | EV001783 | No | Yes | Yes | Yes | Cell; Human-derived; Small Animal |
| FS0356 | NADH-oxidase nanozyme | Acidic pH | EV001786 | No | Yes | No | Yes | Cell; Small Animal |
| FS0392 | Senolytic/proangiogenic smart hydrogel-coated implant | ROS | EV001789 | No | Yes | No | No | Cell; Small Animal |
| FS0399 | FMAG nanoplatform | ROS | EV001791 | Yes | Yes | No | No | Cell; Small Animal |
| FS0402 | PDA@RES NIR-responsive core–shell nanoplatform | NIR light | EV001795 | No | Yes | Yes | Yes | Cell; Small Animal |
| FS0406 | miR-200b-3p-loaded Poloxamer-407 hydrogel | Temperature | EV001797 | No | No | No | No | Small Animal |
| FS0428 | Self-healing hydrogels loaded with Spatholobi Caulis | Mechanical strain | EV001800 | Yes | Yes | Yes | No | Cell; Small Animal |
| FS0472 | HydroWrap hydrogen-sulfide-delivery hydrogel | NIR light | EV001811 | Yes | Yes | Yes | Yes | Cell; Small Animal |
| FS0487 | 3D-printed titanium scaffolds coated with a multifunctional photothermal-responsive hydrogel | NIR light | EV001813 | No | Yes | Yes | Yes | Cell; Small Animal |
| FS0490 | EGCG@MAB hydrogel | Acidic pH | EV001814 | No | Yes | Yes | Yes | Cell; Small Animal |
| FS0538 | ZIF-8/resveratrol nanoformulation | Acidic pH | EV001817 | No | Yes | No | Yes | Cell; Human-derived |
| FS0657 | Quercetin/HA-loaded TG-18 hydrogel | Senescence-associated esterase/SASP-associated enzymatic milieu | EV001828 | Yes | Yes | No | Yes | Cell; Small Animal |
| FS0810 | ASD all-in-one smart hydrogel dressing | Acidic pH | EV001836 | Yes | Yes | No | Yes | Cell; Small Animal |
| FS0834 | Nanocatalytic MOF-coated titanium implant | Acidic pH | EV001841 | No | Yes | No | Yes | Cell; Small Animal |
| FS1052 | EMHS HxMoO3-loaded bone-targeting lipid nanoparticles | Acidic pH | EV001844 | No | Yes | Yes | Yes | Cell; Human-derived; Small Animal |
| FS0018 | PSeR multihierarchy ROS-regulated hydrogel | ROS | EV001847 | No | No | No | No | Cell; Small Animal |
| FS0112 | YDC-Gel-Zn pseudo-mussel-foot-protein hydrogel adhesive | Acidic pH | EV001851 | No | Yes | No | Yes | Cell; Small Animal |
| FS0135 | Electrostatically co-assembled extracellular-vesicle-infused hydrogel | Mechanical force | EV001853 | No | Yes | No | Yes | Cell; Small Animal |
| FS0248 | Smart spatiotemporal drug-delivery hydrogels | ROS | EV001863 | No | Yes | Yes | Yes | Cell; Human-derived; Small Animal |
| FS0270 | PTHrP supramolecular-peptide-loaded dual-network hydrogel | Mechanical force | EV001865 | No | Yes | No | Yes | Cell; Small Animal |
| FS0312 | HG-QNT nanocomposite hydrogel | Mechanical force | EV001872 | No | Yes | Yes | No | Cell |
| FS0361 | Hydrocaffeic-acid–chitosan/EGTA hydrogel | Enzyme | EV001877 | No | No | No | No | Cell; Small Animal |
| FS0402 | PDA@RES NIR-responsive core–shell nanoplatform | Acidic pH | EV001881 | No | Yes | Yes | Yes | Cell; Small Animal |
| FS0461 | Active Magnesium Boride/Alginate Hydrogels | Acidic pH | EV001887 | No | Yes | Yes | No | Cell; Small Animal |
| FS0534 | F@GP galactose-modified Fe3O4-loaded PLGA nanoparticles | Acidic pH | EV001890 | Yes | Yes | No | No | Cell; Small Animal |
| FS0534 | F@GP galactose-modified Fe3O4-loaded PLGA nanoparticles | β-galactosidase | EV001891 | Yes | Yes | No | No | Cell; Small Animal |
| FS0913 | GLRX3+ extracellular-vesicle redox-modulatory hydrogel | ROS | EV001904 | No | Yes | Yes | Yes | Cell; Small Animal |
| FS1054 | HPTC ECM-mimetic injectable hydrogel | Mechanical force | EV001906 | No | Yes | No | Yes | Cell; Ex vivo; Small Animal |

Note: Fifty-five studies and 55 material platforms contributed 64 RESPONDS TO edges because some materials responded to more than one stimulus.

**Table S7. Same-record target-cell, mechanism, and regeneration modules**

| **Record ID** | **MaterialPlatform** | **TargetCell** | **Mechanism** | **RegenerativeEndpoint** | **ValidationStage** | **TARGETS Evidence ID** | **MODULATES Evidence ID** | **PROMOTES Evidence ID** |
| --- | --- | --- | --- | --- | --- | --- | --- | --- |
| FS0036 | CCM@CS-HG hydrogel | Fibroblasts/keratinocytes/skin cells | ROS/oxidative-stress control | Wound healing/skin regeneration | Cell; Small Animal | EV000088 | EV000083 | EV000084 |
| FS0043 | PAA–EDA-grafted porous PEEK scaffold | Mesenchymal stem/stromal cells | βPIX–ITGB1–RAC1–NOX1 signaling | Bone regeneration/osteogenesis | Cell; Small Animal | EV000110 | EV000108 | EV000109 |
| FS0082 | 3D-printed PCL/nHA/SA/COL loaded with M2 macrophage-derived exosomes | Macrophages/innate immune cells | Paracrine/EV-mediated signaling | Bone regeneration/osteogenesis | Cell; Small Animal | EV000203 | EV000200 | EV000201 |
| FS0082 | 3D-printed PCL/nHA/SA/COL loaded with M2 macrophage-derived exosomes | Mesenchymal stem/stromal cells | Paracrine/EV-mediated signaling | Bone regeneration/osteogenesis | Cell; Small Animal | EV000204 | EV000200 | EV000201 |
| FS0122 | GM-Gel glycyrrhizic-acid-microsphere-loaded GEP/chitosan hydrogel | Macrophages/innate immune cells | Immune modulation/macrophage polarization | Wound healing/skin regeneration | Cell; Small Animal | EV000307 | EV000305 | EV000306 |
| FS0136 | A20-CRISPRa injectable microspheres | Mesenchymal stem/stromal cells | NF-κB/SASP/inflammatory signaling | Bone regeneration/osteogenesis | Cell; Small Animal | EV000360 | EV000358 | EV000359 |
| FS0142 | HPQ@K superlubricative drug-delivery nanoparticles | Chondrocytes/cartilage progenitors | ROS/oxidative-stress control | Cartilage regeneration/chondroprotection | Cell; Human-derived; Small Animal | EV000368 | EV000365 | EV000366 |
| FS0207 | ABT-263-loaded asymmetric composite wound dressing | Endothelial/vascular cells | Senolytic cell clearance | Wound healing/skin regeneration | Cell; Small Animal | EV000517 | EV000515 | EV000516 |
| FS0207 | ABT-263-loaded asymmetric composite wound dressing | Fibroblasts/keratinocytes/skin cells | Senolytic cell clearance | Wound healing/skin regeneration | Cell; Small Animal | EV000518 | EV000515 | EV000516 |
| FS0207 | ABT-263-loaded asymmetric composite wound dressing | Senescent cells | Senolytic cell clearance | Wound healing/skin regeneration | Cell; Small Animal | EV000519 | EV000515 | EV000516 |
| FS0222 | Zn@MOF-gel dual-polyphenol ECM-mimetic hydrogel | Nucleus pulposus/annulus fibrosus cells | NF-κB/SASP/inflammatory signaling | Intervertebral-disc regeneration | Cell; Large Animal; Small Animal | EV000550 | EV000548 | EV000549 |
| FS0226 | YBEV-loaded GelMA hydrogel | Senescent cells | Mitochondrial homeostasis/mitophagy | Wound healing/skin regeneration | Cell; Small Animal | EV000568 | EV000566 | EV000567 |
| FS0238 | PEG-HS/TiO2 nanocomposite | Endothelial/vascular cells | ROS/oxidative-stress control | Wound healing/skin regeneration | Cell; Human-derived; Small Animal | EV000604 | EV000602 | EV000603 |
| FS0238 | PEG-HS/TiO2 nanocomposite | Macrophages/innate immune cells | ROS/oxidative-stress control | Wound healing/skin regeneration | Cell; Human-derived; Small Animal | EV000605 | EV000602 | EV000603 |
| FS0248 | Smart spatiotemporal drug-delivery hydrogels | Endothelial/vascular cells | ROS/oxidative-stress control | Bone regeneration/osteogenesis | Cell; Human-derived; Small Animal | EV000646 | EV000644 | EV000645 |
| FS0248 | Smart spatiotemporal drug-delivery hydrogels | Mesenchymal stem/stromal cells | ROS/oxidative-stress control | Bone regeneration/osteogenesis | Cell; Human-derived; Small Animal | EV000647 | EV000644 | EV000645 |
| FS0279 | Lesion-localized hydrogels functionalized with engineered extracellular matrix | Chondrocytes/cartilage progenitors | Mitochondrial homeostasis/mitophagy | Cartilage regeneration/chondroprotection | Cell; Small Animal | EV000693 | EV000691 | EV000692 |
| FS0282 | Nanoceria-infused PCL nanofibrous scaffold | Senescent cells | ROS/oxidative-stress control | Bone regeneration/osteogenesis | Cell | EV000702 | EV000700 | EV000701 |
| FS0283 | Orbital hybridization-mediated nanozymes | Endothelial/vascular cells | ROS/oxidative-stress control | Bone regeneration/osteogenesis | Cell; Small Animal | EV000710 | EV000708 | EV000709 |
| FS0283 | Orbital hybridization-mediated nanozymes | Mesenchymal stem/stromal cells | ROS/oxidative-stress control | Bone regeneration/osteogenesis | Cell; Small Animal | EV000711 | EV000708 | EV000709 |
| FS0296 | MMZ@Si–P53 hydrogel | Mesenchymal stem/stromal cells | p16/p21/p53–Rb cell-cycle arrest | Bone regeneration/osteogenesis | Cell; Small Animal | EV000737 | EV000735 | EV000736 |
| FS0296 | MMZ@Si–P53 hydrogel | Neural stem cells/neurons/glia | p16/p21/p53–Rb cell-cycle arrest | Bone regeneration/osteogenesis | Cell; Small Animal | EV000738 | EV000735 | EV000736 |
| FS0322 | CAP-CD56+CD271+ BMSC-exosome-loaded PVA/SA hydrogel | Chondrocytes/cartilage progenitors | p16/p21/p53–Rb cell-cycle arrest | Cartilage regeneration/chondroprotection | Cell; Small Animal | EV000804 | EV000801 | EV000802 |
| FS0322 | CAP-CD56+CD271+ BMSC-exosome-loaded PVA/SA hydrogel | Mesenchymal stem/stromal cells | p16/p21/p53–Rb cell-cycle arrest | Cartilage regeneration/chondroprotection | Cell; Small Animal | EV000805 | EV000801 | EV000802 |
| FS0338 | MOTS-c-modified functional self-assembly peptide hydrogels | Mesenchymal stem/stromal cells | AMPK–mTOR–sirtuin signaling | Intervertebral-disc regeneration | Cell; Human-derived; Small Animal | EV000849 | EV000847 | EV000848 |
| FS0338 | MOTS-c-modified functional self-assembly peptide hydrogels | Nucleus pulposus/annulus fibrosus cells | AMPK–mTOR–sirtuin signaling | Intervertebral-disc regeneration | Cell; Human-derived; Small Animal | EV000850 | EV000847 | EV000848 |
| FS0346 | Multifunctional pH-responsive microneedles | Periodontal/dental stem cells | Angiogenic/HIF–VEGF signaling | Bone regeneration/osteogenesis | Cell; Human-derived; Small Animal | EV000869 | EV000867 | EV000868 |
| FS0350 | Senescence-targeted NAD+-dependent SIRT1-activating nanoplatform | Mesenchymal stem/stromal cells | AMPK–mTOR–sirtuin signaling | Bone regeneration/osteogenesis | Cell; Ex vivo; Small Animal | EV000881 | EV000879 | EV000880 |
| FS0384 | α-Arbutin-crosslinked chitosan/gelatin film | Osteoblasts/osteocytes/osteoclasts | ROS/oxidative-stress control | Bone regeneration/osteogenesis | Cell; Small Animal | EV000965 | EV000963 | EV000964 |
| FS0384 | α-Arbutin-crosslinked chitosan/gelatin film | Senescent cells | ROS/oxidative-stress control | Bone regeneration/osteogenesis | Cell; Small Animal | EV000966 | EV000963 | EV000964 |
| FS0400 | Multimodal ROS-logic-gated therapeutic platform | Senescent cells | ROS/oxidative-stress control | Bone regeneration/osteogenesis | Cell; Small Animal | EV001037 | EV001033 | EV001034 |
| FS0402 | PDA@RES NIR-responsive core–shell nanoplatform | Endothelial/vascular cells | Mitochondrial homeostasis/mitophagy | Wound healing/skin regeneration | Cell; Small Animal | EV001045 | EV001043 | EV001044 |
| FS0436 | CeO2 nanozymes | Mesenchymal stem/stromal cells | ROS/oxidative-stress control | Bone regeneration/osteogenesis | Cell; Small Animal | EV001111 | EV001109 | EV001110 |
| FS0436 | CeO2 nanozymes | Osteoblasts/osteocytes/osteoclasts | ROS/oxidative-stress control | Bone regeneration/osteogenesis | Cell; Small Animal | EV001112 | EV001109 | EV001110 |
| FS0472 | HydroWrap hydrogen-sulfide-delivery hydrogel | Macrophages/innate immune cells | NF-κB/SASP/inflammatory signaling | Bone regeneration/osteogenesis | Cell; Small Animal | EV001226 | EV001223 | EV001224 |
| FS0487 | 3D-printed titanium scaffolds coated with a multifunctional photothermal-responsive hydrogel | Macrophages/innate immune cells | ROS/oxidative-stress control | Bone regeneration/osteogenesis | Cell; Small Animal | EV001245 | EV001243 | EV001244 |
| FS0487 | 3D-printed titanium scaffolds coated with a multifunctional photothermal-responsive hydrogel | Mesenchymal stem/stromal cells | ROS/oxidative-stress control | Bone regeneration/osteogenesis | Cell; Small Animal | EV001246 | EV001243 | EV001244 |
| FS0490 | EGCG@MAB hydrogel | Senescent cells | Mitochondrial homeostasis/mitophagy | Wound healing/skin regeneration | Cell; Small Animal | EV001261 | EV001258 | EV001259 |
| FS0598 | α-Klotho gene-activated scaffold | Endothelial/vascular cells | Angiogenic/HIF–VEGF signaling | Wound healing/skin regeneration | Cell | EV001387 | EV001385 | EV001386 |
| FS0598 | α-Klotho gene-activated scaffold | Mesenchymal stem/stromal cells | Angiogenic/HIF–VEGF signaling | Wound healing/skin regeneration | Cell | EV001388 | EV001385 | EV001386 |
| FS0624 | Lycium barbarum-derived carbon dots | Mesenchymal stem/stromal cells | Apoptosis/cell-survival regulation | Bone regeneration/osteogenesis | Cell; Small Animal | EV001396 | EV001394 | EV001395 |
| FS0696 | miR-29b-5p-loaded stem-cell-homing hydrogel | Chondrocytes/cartilage progenitors | Epigenetic/chromatin regulation | Cartilage regeneration/chondroprotection | Cell; Small Animal | EV001463 | EV001461 | EV001462 |
| FS0709 | Cerium-oxide-nanoparticle-carrying hUCMSCs | Mesenchymal stem/stromal cells | ROS/oxidative-stress control | Tendon/ligament regeneration | Cell; Small Animal | EV001483 | EV001481 | EV001482 |
| FS0738 | Gelatin-microcarrier-based MSC microtissues | Mesenchymal stem/stromal cells | Apoptosis/cell-survival regulation | Neural regeneration/function | Cell; Small Animal | EV001510 | EV001508 | EV001509 |
| FS0765 | Antibody-functionalized copper-sulfate nanoparticles | Chondrocytes/cartilage progenitors | Apoptosis/cell-survival regulation | Cartilage regeneration/chondroprotection | Cell; Small Animal | EV001520 | EV001518 | EV001519 |
| FS0765 | Antibody-functionalized copper-sulfate nanoparticles | Senescent cells | Apoptosis/cell-survival regulation | Cartilage regeneration/chondroprotection | Cell; Small Animal | EV001521 | EV001518 | EV001519 |
| FS0783 | Calcium-silicate ion-releasing biomaterial | Fibroblasts/keratinocytes/skin cells | p16/p21/p53–Rb cell-cycle arrest | Wound healing/skin regeneration | Cell; Small Animal | EV001535 | EV001533 | EV001534 |
| FS0913 | GLRX3+ extracellular-vesicle redox-modulatory hydrogel | Mesenchymal stem/stromal cells | ROS/oxidative-stress control | Intervertebral-disc regeneration | Cell; Small Animal | EV001636 | EV001634 | EV001635 |
| FS0913 | GLRX3+ extracellular-vesicle redox-modulatory hydrogel | Nucleus pulposus/annulus fibrosus cells | ROS/oxidative-stress control | Intervertebral-disc regeneration | Cell; Small Animal | EV001637 | EV001634 | EV001635 |
| FS1052 | EMHS HxMoO3-loaded bone-targeting lipid nanoparticles | Endothelial/vascular cells | Mitochondrial homeostasis/mitophagy | Bone regeneration/osteogenesis | Cell; Human-derived; Small Animal | EV001700 | EV001698 | EV001699 |
| FS1052 | EMHS HxMoO3-loaded bone-targeting lipid nanoparticles | Mesenchymal stem/stromal cells | Mitochondrial homeostasis/mitophagy | Bone regeneration/osteogenesis | Cell; Human-derived; Small Animal | EV001701 | EV001698 | EV001699 |

Note: All paths are same-record co-supported configurations and do not establish causal mediation.

**Table S8. Validated-core missing-link records**

| **Gap type** | **Record ID** | **MaterialPlatform** | **Supported relation configuration** | **Missing SUPPORTED relation** |
| --- | --- | --- | --- | --- |
| TYPE A | FS0038 | Iron oxide nanoparticles-driven mitochondrial renewal | RESPONDS TO + TARGETS | PROMOTES |
| TYPE A | FS0056 | Polyphenol-armored miR-140-5p nanogene-loaded bioadhesive hydrogel | RESPONDS TO + TARGETS | PROMOTES |
| TYPE A | FS0067 | Asp10SAC4A supramolecular senolytic nanoplatform | RESPONDS TO + TARGETS | PROMOTES |
| TYPE A | FS0073 | Self-powered triboelectric-responsive EV-releasing microneedles | RESPONDS TO + TARGETS | PROMOTES |
| TYPE A | FS0105 | Locally activated TGF-β3/MgSi immunoregulatory nanofiber patch | RESPONDS TO + TARGETS | PROMOTES |
| TYPE A | FS0133 | OGPQ/G-NZ quercetin–greigite nanozyme hydrogel | RESPONDS TO + TARGETS | PROMOTES |
| TYPE A | FS0147 | TT@P-Gel hydrogel | RESPONDS TO + TARGETS | PROMOTES |
| TYPE A | FS0148 | Se/PRP-OGel hydrogel | RESPONDS TO + TARGETS | PROMOTES |
| TYPE A | FS0166 | IL37OI@MS responsive hydrogel microspheres | RESPONDS TO + TARGETS | PROMOTES |
| TYPE A | FS0173 | Hydrogel-coated micro/nanostructured titanium implant | RESPONDS TO + TARGETS | PROMOTES |
| TYPE A | FS0178 | D-EV-loaded thermosensitive injectable hydrogel | RESPONDS TO + TARGETS | PROMOTES |
| TYPE A | FS0183 | ICAM1-directed MnO2@ZIF8/Tanshinone-IIA nanoplatform | RESPONDS TO + TARGETS | PROMOTES |
| TYPE A | FS0223 | Fisetin-functionalized photoresponsive DNA nanocage | RESPONDS TO + TARGETS | PROMOTES |
| TYPE A | FS0232 | GPQ pH-responsive anti-senescence hydrogel | RESPONDS TO + TARGETS | PROMOTES |
| TYPE A | FS0247 | PCC1/PHCF-Gel senolytic hydrogel | RESPONDS TO + TARGETS | PROMOTES |
| TYPE A | FS0252 | Immunomodulating regenerating hydrogel | RESPONDS TO + TARGETS | PROMOTES |
| TYPE A | FS0308 | OSK-protein-delivery and inflammatory-niche-remodeling platform | RESPONDS TO + TARGETS | PROMOTES |
| TYPE A | FS0312 | HG-QNT nanocomposite hydrogel | RESPONDS TO + TARGETS | PROMOTES |
| TYPE A | FS0392 | Senolytic/proangiogenic smart hydrogel-coated implant | RESPONDS TO + TARGETS | PROMOTES |
| TYPE A | FS0399 | FMAG nanoplatform | RESPONDS TO + TARGETS | PROMOTES |
| TYPE A | FS0419 | Senile-osteoporosis titanium implant | RESPONDS TO + TARGETS | PROMOTES |
| TYPE A | FS0428 | Self-healing hydrogels loaded with Spatholobi Caulis | RESPONDS TO + TARGETS | PROMOTES |
| TYPE A | FS0461 | Active Magnesium Boride/Alginate Hydrogels | RESPONDS TO + TARGETS | PROMOTES |
| TYPE A | FS0462 | LT-NPs nanoplatform | RESPONDS TO + TARGETS | PROMOTES |
| TYPE A | FS0534 | F@GP galactose-modified Fe3O4-loaded PLGA nanoparticles | RESPONDS TO + TARGETS | PROMOTES |
| TYPE B | FS0062 | A-Lipo/PAHM lesion-localized hydrogel microspheres | TARGETS + MODULATES | PROMOTES |
| TYPE B | FS0090 | TSH-Q quercetin-loaded temperature-sensitive hydrogel | TARGETS + MODULATES | PROMOTES |
| TYPE B | FS0102 | Porcine small-intestinal-submucosa extracellular-matrix scaffold | TARGETS + MODULATES | PROMOTES |
| TYPE B | FS0111 | Retinoic-acid-loaded cartilage organoids | TARGETS + MODULATES | PROMOTES |
| TYPE B | FS0114 | NH@K nanozyme-reinforced hydrogel | TARGETS + MODULATES | PROMOTES |
| TYPE B | FS0124 | Curcumin-loaded liposomes | TARGETS + MODULATES | PROMOTES |
| TYPE B | FS0133 | OGPQ/G-NZ quercetin–greigite nanozyme hydrogel | TARGETS + MODULATES | PROMOTES |
| TYPE B | FS0147 | TT@P-Gel hydrogel | TARGETS + MODULATES | PROMOTES |
| TYPE B | FS0148 | Se/PRP-OGel hydrogel | TARGETS + MODULATES | PROMOTES |
| TYPE B | FS0166 | IL37OI@MS responsive hydrogel microspheres | TARGETS + MODULATES | PROMOTES |
| TYPE B | FS0168 | SM@ME exosome-loaded methacrylated-silk-fibroin hydrogel | TARGETS + MODULATES | PROMOTES |
| TYPE B | FS0173 | Hydrogel-coated micro/nanostructured titanium implant | TARGETS + MODULATES | PROMOTES |
| TYPE B | FS0178 | D-EV-loaded thermosensitive injectable hydrogel | TARGETS + MODULATES | PROMOTES |
| TYPE B | FS0187 | Mn3O4-nanoparticle photothermal hydrogel | TARGETS + MODULATES | PROMOTES |
| TYPE B | FS0196 | Polyacrylamide/alginate viscoelastic hydrogel | TARGETS + MODULATES | PROMOTES |
| TYPE B | FS0212 | Prussian Blue nanoparticles | TARGETS + MODULATES | PROMOTES |
| TYPE B | FS0234 | Surface-elastic StG hydrogels | TARGETS + MODULATES | PROMOTES |
| TYPE B | FS0239 | FN9-10ELP extracellular-matrix-mimetic fusion protein | TARGETS + MODULATES | PROMOTES |
| TYPE B | FS0244 | Quercetin-treated human-auricular-chondrocyte/GelMA construct | TARGETS + MODULATES | PROMOTES |
| TYPE B | FS0252 | Immunomodulating regenerating hydrogel | TARGETS + MODULATES | PROMOTES |
| TYPE B | FS0278 | Synovium-targeting senolytic liposomes plus M2-macrophage exosomes | TARGETS + MODULATES | PROMOTES |
| TYPE B | FS0306 | MSC-educated tendon (METn) construct | TARGETS + MODULATES | PROMOTES |
| TYPE B | FS0308 | OSK-protein-delivery and inflammatory-niche-remodeling platform | TARGETS + MODULATES | PROMOTES |
| TYPE B | FS0312 | HG-QNT nanocomposite hydrogel | TARGETS + MODULATES | PROMOTES |
| TYPE B | FS0326 | AAV-loaded extracellular-vesicle biomimetic gene-delivery system | TARGETS + MODULATES | PROMOTES |
| TYPE B | FS0382 | Nanozyme-Integrated 3D-Printed Gradient Scaffold | TARGETS + MODULATES | PROMOTES |
| TYPE B | FS0404 | synFGF18 mRNA lipid nanoparticles | TARGETS + MODULATES | PROMOTES |
| TYPE B | FS0428 | Self-healing hydrogels loaded with Spatholobi Caulis | TARGETS + MODULATES | PROMOTES |
| TYPE B | FS0430 | HIF1A@NNP nanoplatform | TARGETS + MODULATES | PROMOTES |
| TYPE B | FS0439 | TA-PFC/PCK fiber scaffold | TARGETS + MODULATES | PROMOTES |
| TYPE B | FS0446 | Biomimetic hydrogel delivers engineered exosomes | TARGETS + MODULATES | PROMOTES |
| TYPE B | FS0450 | Collagen-coated polyacrylamide hydrogels | TARGETS + MODULATES | PROMOTES |
| TYPE B | FS0461 | Active Magnesium Boride/Alginate Hydrogels | TARGETS + MODULATES | PROMOTES |
| TYPE B | FS0482 | miR-146a-5p-engineered exosomes | TARGETS + MODULATES | PROMOTES |
| TYPE B | FS0637 | Microtopographic polymer substrates | TARGETS + MODULATES | PROMOTES |
| TYPE B | FS0642 | Baghdadite Ceramics | TARGETS + MODULATES | PROMOTES |
| TYPE B | FS0788 | Ruxolitinib-loaded hierarchically biomimetic nanostructural scaffold | TARGETS + MODULATES | PROMOTES |
| TYPE B | FS0843 | Extracellular-vesicle-conjugated functional-matrix hydrogel | TARGETS + MODULATES | PROMOTES |
| TYPE B | FS0882 | Tonsil-derived mesenchymal stem cells incorporated in reactive oxygen species-releasing hydrogel | TARGETS + MODULATES | PROMOTES |
| TYPE B | FS0912 | Dual-drug-encapsulated liposomal nanoparticles | TARGETS + MODULATES | PROMOTES |
| TYPE B | FS0916 | Honey-incorporated electrospun nanofiber membrane | TARGETS + MODULATES | PROMOTES |
| TYPE B | FS1020 | SRT1720-loaded calcium-phosphate/magnesium-phosphate nanocarriers | TARGETS + MODULATES | PROMOTES |
| TYPE C | FS0044 | Lubricated hydrogel with STING-inhibiting EXOs | DELIVERS + PROMOTES | MODULATES |
| TYPE C | FS0117 | Senomorphic Small Extracellular Vesicles Delivered by a Tissue-Adhesive α-Lipoic-Acid Hydrogel | DELIVERS + PROMOTES | MODULATES |
| TYPE C | FS0134 | Electrospun scaffold–bioreactor system | DELIVERS + PROMOTES | MODULATES |
| TYPE C | FS0184 | GEA/NGel senolysis-targeting bioenergetic nanoplatform | DELIVERS + PROMOTES | MODULATES |
| TYPE C | FS0186 | (ZIF-8@CeO2)@GOx-loaded sprayable GelMA hydrogel | DELIVERS + PROMOTES | MODULATES |
| TYPE C | FS0213 | FGF-4/IGF-1-loaded cationic thermosensitive polymer scaffold | DELIVERS + PROMOTES | MODULATES |
| TYPE C | FS0295 | GelMA@ZIF-8/PGE microsphere–hydrogel platform | DELIVERS + PROMOTES | MODULATES |
| TYPE C | FS0313 | TPG@ChSMA mitochondrial-targeted hydrogel | DELIVERS + PROMOTES | MODULATES |
| TYPE C | FS0367 | Adhesive injectable NRF2-modulating hydrogel microspheres | DELIVERS + PROMOTES | MODULATES |
| TYPE C | FS0376 | Mitochondrial-metabolism/efferocytosis-modulating microcarriers | DELIVERS + PROMOTES | MODULATES |
| TYPE C | FS0389 | Enucleated-MSC-derived mitochondria-transfer microvesicles | DELIVERS + PROMOTES | MODULATES |
| TYPE C | FS0391 | rhBMP-2/rapamycin co-treatment without an engineered carrier | DELIVERS + PROMOTES | MODULATES |
| TYPE C | FS0394 | Quercetin-loaded homogeneous PLGA microspheres | DELIVERS + PROMOTES | MODULATES |
| TYPE C | FS0397 | Cordycepin-Loaded Dental Pulp Stem Cell-Derived Exosomes | DELIVERS + PROMOTES | MODULATES |
| TYPE C | FS0409 | Curcumin-loaded milk-sEV/platelet-membrane hybrid vesicles | DELIVERS + PROMOTES | MODULATES |
| TYPE C | FS0433 | Quercetin-loaded aligned-nanowire hydrogel | DELIVERS + PROMOTES | MODULATES |
| TYPE C | FS0507 | Tannic-acid-loaded calcium-alginate/silk-fibroin-peptide/Bletilla-polysaccharide microspheres | DELIVERS + PROMOTES | MODULATES |
| TYPE C | FS0538 | ZIF-8/resveratrol nanoformulation | DELIVERS + PROMOTES | MODULATES |
| TYPE C | FS0657 | Quercetin/HA-loaded TG-18 hydrogel | DELIVERS + PROMOTES | MODULATES |
| TYPE C | FS0810 | ASD all-in-one smart hydrogel dressing | DELIVERS + PROMOTES | MODULATES |
| TYPE C | FS1040 | Hairyvein-agrimony-nanovesicle-loaded microneedles | DELIVERS + PROMOTES | MODULATES |

Note: Absence of a SUPPORTED relation from the validated core does not establish that the corresponding experiment was absent from the source publication.

**Table S9. Competency questions and independent query benchmark**

| **CQ ID** | **Short query description** | **Required relation(s)** | **Additional constraint** | **Result count** | **Precision** | **Recall** | **F1** | **Exact-set match** |
| --- | --- | --- | --- | --- | --- | --- | --- | --- |
| CQ01 | Response–target–regeneration | RESPONDS TO; TARGETS; PROMOTES | Same Record ID and material. | 24 | 1.00 | 1.00 | 1.00 | YES |
| CQ02 | Response–release | RESPONDS TO; RELEASES | Same Record ID and material; direct RELEASES edge. | 17 | 1.00 | 1.00 | 1.00 | YES |
| CQ03 | Response–mechanism–regeneration | RESPONDS TO; MODULATES; PROMOTES | Same Record ID and material. | 12 | 1.00 | 1.00 | 1.00 | YES |
| CQ04 | Cargo–target–animal validation | DELIVERS; TARGETS; VALIDATED AT | Same Record ID and material; Small Animal or Large Animal. | 50 | 1.00 | 1.00 | 1.00 | YES |
| CQ05 | Cell–mechanism–regeneration | TARGETS; MODULATES; PROMOTES | Same Record ID and material. | 35 | 1.00 | 1.00 | 1.00 | YES |
| CQ06 | Composition–fabrication–non-cell validation | COMPOSED OF; FABRICATED BY; VALIDATED AT | Same Record ID and material; validation stage is not Cell. | 53 | 1.00 | 1.00 | 1.00 | YES |
| CQ07 | Release–mechanism | RELEASES; MODULATES | Same Record ID and material. | 12 | 1.00 | 1.00 | 1.00 | YES |
| CQ08 | Multi-cargo regeneration | DELIVERS; PROMOTES | At least two distinct cargo nodes and a PROMOTES edge. | 2 | 1.00 | 1.00 | 1.00 | YES |
| CQ09 | Multi-cell mechanism | TARGETS; MODULATES | At least two distinct target-cell nodes and a MODULATES edge. | 30 | 1.00 | 1.00 | 1.00 | YES |
| CQ10 | Mechanism–endpoint–animal validation | MODULATES; PROMOTES; VALIDATED AT | Small Animal or Large Animal. | 37 | 1.00 | 1.00 | 1.00 | YES |
| CQ11 | Response–target validated-core gap | RESPONDS TO; TARGETS; NOT PROMOTES | RESPONDS TO and TARGETS present; no SUPPORTED PROMOTES. | 25 | 1.00 | 1.00 | 1.00 | YES |
| CQ12 | Target–mechanism validated-core gap | TARGETS; MODULATES; NOT PROMOTES | TARGETS and MODULATES present; no SUPPORTED PROMOTES. | 42 | 1.00 | 1.00 | 1.00 | YES |
| CQ13 | Cargo–endpoint validated-core gap | DELIVERS; PROMOTES; NOT MODULATES | DELIVERS and PROMOTES present; no SUPPORTED MODULATES. | 21 | 1.00 | 1.00 | 1.00 | YES |
| CQ14 | Human-derived/Clinical relation coverage | VALIDATED AT; TARGETS/MODULATES/PROMOTES | Human-derived or Clinical plus TARGETS, MODULATES, or PROMOTES. | 49 | 1.00 | 1.00 | 1.00 | YES |
| CQ15 | High evidence-density materials | At least five distinct relation types | At least eight supported edges spanning at least five relation types. | 95 | 1.00 | 1.00 | 1.00 | YES |

Note: Benchmark metrics quantify agreement between two independently implemented query procedures over the frozen graph and do not estimate biomedical ground-truth accuracy.

**Supplementary Table S10. Relation-specific criteria for evidence-strength assignment in SenescenceBioKG**

| **Relation family** | **Included relation(s)** | **High evidence** | **Medium evidence** | **Low evidence** |
| --- | --- | --- | --- | --- |
| **A. Platform design and construction** | USES; COMPOSED_OF; FABRICATED_BY | Assigned High where frozen records retained direct same-record identification of a design mode, material constituent, or fabrication operation; detailed High/Medium thresholds were not separately formalized. | Not separately formalized in the frozen adjudication records; classification followed relation-specific evidence directness and was resolved during semantic review. | No Low edges occur in this family; a separate Low threshold was not formalized in the frozen adjudication records. |
| **B. Cargo delivery and release** | DELIVERS; RELEASES | Assigned High where frozen records directly identified incorporated cargo or documented release; detailed High/Medium thresholds were not separately formalized in the adjudication records. | Not separately formalized in the frozen adjudication records; classification followed relation-specific evidence directness and was resolved during semantic review. | No Low edges occur in this family; a separate Low threshold was not formalized in the frozen adjudication records. |
| **C. Material responsiveness** | RESPONDS_TO | Assigned High where a defined stimulus directly changed measured material or platform behavior; the frozen records did not separately formalize the High/Medium threshold. | A qualifying stimulus-dependent material response was retained during semantic review, but the frozen records did not separately formalize the High/Medium threshold. | No Low RESPONDS_TO edges occur; ROS reduction, antioxidant activity, sustained release, or responsive naming alone were not accepted as supporting evidence. |
| **D. Biological action and regeneration** | TARGETS; MODULATES; PROMOTES | Study-specific evidence supported the tested cell, mechanism, or regenerative endpoint. A verified High MODULATES example used BNIP3 knockdown; family-wide tier thresholds were not separately formalized. | Not separately formalized in the frozen adjudication records; classification followed relation-specific evidence directness and was resolved during semantic review. | Low occurred only for MODULATES (12 edges) and reflected indirect but study-specific mechanistic support that still met the MODULATES criterion; background discussion, cited mechanisms, and correlation alone remained excluded. |
| **E. Validation setting** | VALIDATED_AT | Assigned High where frozen records directly identified the reported experimental setting and satisfied the assigned validation category; detailed High/Medium thresholds were not separately formalized. | Not separately formalized in the frozen adjudication records; classification followed relation-specific evidence directness and was resolved during semantic review. | No Low VALIDATED_AT edges occur in the frozen graph; a separate Low threshold was not formalized in the adjudication records. |

Note. Evidence strength was assigned at the edge level using relation-specific criteria. High, Medium, and Low therefore describe the directness and completeness of evidence within a relation family and should not be interpreted as a common quantitative scale across relation types or as an assessment of overall study quality.

**Representative examples**

• FS0044 | FABRICATED_BY | Click-chemistry crosslinking | EV000118 | High | Epoxy/thiol click chemistry directly fabricated the hydrogel.

• FS0082 | DELIVERS | Exosomes | EV000198 | High | Methods documented exosome loading and full carrier binding.

• FS0402 | RESPONDS_TO | Acidic pH | EV001881 | High | Acidic pH significantly increased the material release rate.

• FS0147 | MODULATES | Mitochondrial homeostasis/mitophagy | EV000373 | High | BNIP3 knockdown suppressed material-induced mitophagy enhancement.

• FS0188 | VALIDATED_AT | Large Animal | EV001858 | High | Regeneration was tested in a rhesus macaque model.
